# Mapping of Some Further Alkylation-Initiated Pathways to Polyheterocyclic Compounds from Indigo and Indirubin

**DOI:** 10.3390/molecules29174242

**Published:** 2024-09-06

**Authors:** Sarfaraz Ali, Patrick M. McCosker, Anthony C. Willis, Stephen G. Pyne, Christopher Richardson, John B. Bremner, Paul A. Keller

**Affiliations:** 1 School of Chemistry and Molecular Bioscience, Molecular Horizons, University of Wollongong, Wollongong, NSW 2522, Australia; sa823@uowmail.edu.au (S.A.); patrickmccosker1992@hotmail.com (P.M.M.); spyne@uow.edu.au (S.G.P.); crichard@uow.edu.au (C.R.); jbremner@uow.edu.au (J.B.B.); 2 Research School of Chemistry, Australian National University, Canberra, ACT 2601, Australia; tonywillis2614@gmail.com

**Keywords:** indigo, indirubin, cascade reaction, spiroimidazole, spirooxazine, biindigoid system

## Abstract

The reaction of indigo with two equivalents of the electrophile ethyl bromoacetate with caesium carbonate as a base result in the formation of structurally complex polyheterocyclics, including a fused spiroimidazole and a spiro[1,3]oxazino derivative, together with a biindigoid-type derivative, through a convenient one-pot reaction. Further assessment of the reaction using five equivalents of the electrophile gave rise to other molecules incorporating the 2-(7,13,14-trioxo-6,7,13,14-tetrahydropyrazino[1,2-*a*:4,3-*a*′]diindol-6-yl) scaffold. The reaction of ethyl bromoacetate with the less reactive indirubin resulted in the synthesis of three derivatives of a new class of polyheterocyclic system via a cascade process, although yields were low. These compounds were derived from the parent indolo[1,2-*b*]pyrrolo[4,3,2-*de*]isoquinoline skeleton. Despite the modest yields of the reactions, they represent quick cascade routes to a variety of heterocycles from cheap starting materials, with these structures otherwise being difficult to synthesise in a traditional stepwise manner. These outcomes also contribute significantly to the detailed understanding of the indigo/indirubin cascade reaction pathways initiated by base-catalysed *N*-alkylation.

## 1. Introduction

**Figure 1:**
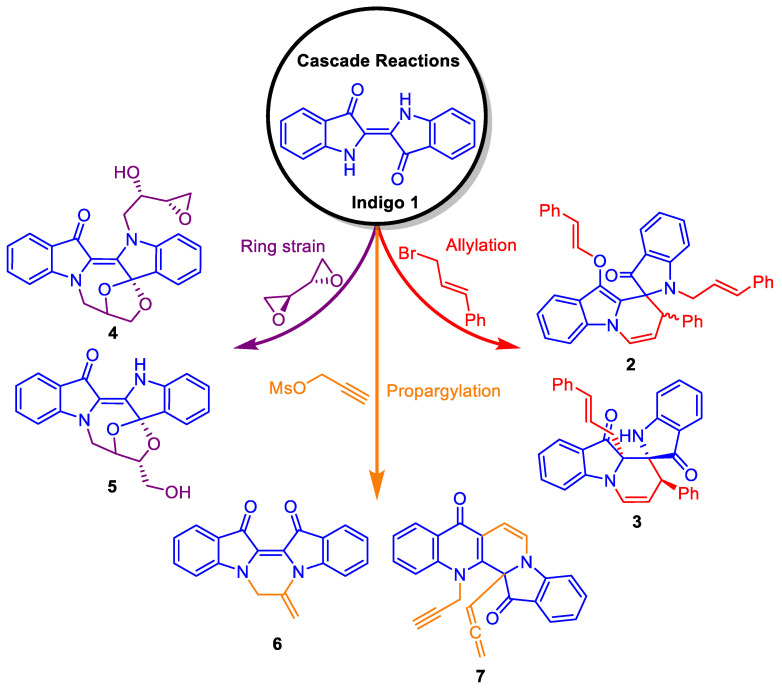
Examples of polyheterocyclic systems arising from cascade reactions of indigo **1**.

Indigo **1** (Figure 1) is a natural dye with a rich history dating back thousands of years [1]. Significant research interest continues in indigo and derivatives not only as colourants or dyes [2] but increasingly in fundamental photophysics [3,4,5] as well as chemical aspects [1,6,7]. Indigo has recently been the subject of great interest in synthetic chemistry due to its aromatic core, which possesses a number of functional groups in close proximity, thus offering a versatile platform for designing cascade reactions. This understanding arises from previous studies that have shown indigo **1** participating in cascade processes with a variety of functionalised electrophiles, yielding unprecedented polyheterocyclic systems [8,9]. For example, the reaction of indigo **1** with allyl halides yielded the 8′*H*-spiro[indoline-2,9′-pyrido[1,2-*a*]indol]-3-one **2** and its Claisen rearranged derivative 8′*H*-spiro[indoline-2,9′-pyrido[1,2-*a*]indole]-3,10′-(9a′*H*)dione **3**. Alternatively, the reaction with bioxirane gave rise to the spiro compounds **4** and **5** (Figure 1) [10].

**Scheme 1:**
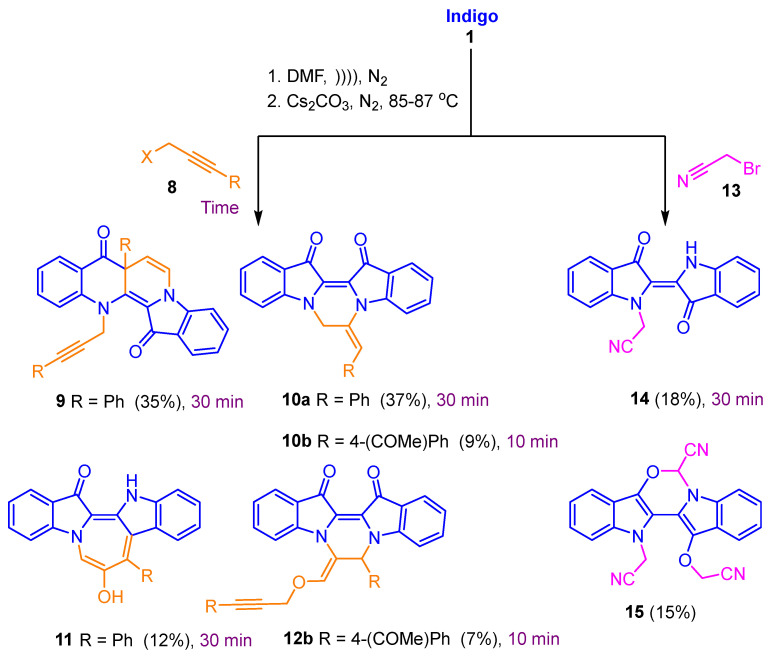
Cascade reaction products from the reaction of indigo with X-CH_2_C≡C-R (X = Br, Cl, OMs or OTs) compared with those from Br-CH_2_CN. Reaction times are presented in purple.

Reactions of indigo **1** with different propargyl derivatives yielded a variety of products, including straightforward addition-cyclisation products, e.g., **6**, as well as cascade-induced products such as the ring-expanded heterocycle **7** (Figure 1) [8]. Further, by altering the leaving group and terminal substitution in the propargyl electrophiles, a variety of diverse outcomes were achieved, with the formation of naphthyridiones (e.g., **9**), pyrazinodiindolodiones (e.g., **10** and **12**), azepinodiindoles (e.g., **11**), and other intriguing heterocyclic systems (Scheme 1) [11]. Further, it was revealed that the reaction of indigo **1** with bromoacetonitrile **13** produced the mono-*N*-alkylated indigo **14** along with the cyclised compound **15**, both in low yield (Scheme 1). This last outcome was interesting as the strongly electron-withdrawing cyano moiety was expected to facilitate anion formation at the adjacent methylene carbon in alkylated intermediates, with subsequent cascade cyclisation reactions producing new polycyclic derivatives.

**Figure 2:**
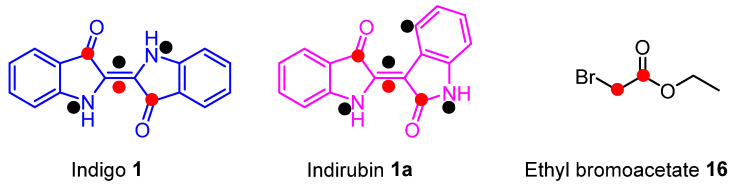
The structures of indigo **1**, indirubin **1a**, and ethyl bromoacetate **16** show electrophilic (●) and nucleophilic sites (●).

The outcomes of these reactions have demonstrated an ability to swiftly access structurally diverse heterocycles, yielding a plethora of new chemical compounds with a possible wide range of applications. They also show that the use of an electrophile containing an electron-withdrawing substituent had a significant effect on the outcome of cascade reactions of indigo **1**. One of the major aspirations of this line of research was to investigate the cascade chemistry of indigo **1** to the point of predictability, thus enabling access to unprecedented polyheterocyclic compounds while exploring diverse molecular space. Therefore, the aim of this current study was to further investigate the *N*-alkylation-initiated chemistry of indigo **1** using electrophiles that contained an electron-withdrawing substituent. Ethyl bromoacetate **16** was chosen as it contained a leaving group to allow for the initial *N*-alkylation of indigo. The presence of the ester-activated methylene group was also anticipated to enable carbanion formation under the basic conditions of the typical cascade reactions, while the ester carbonyl also presents a further electrophilic site (Figure 2), a combination primed to allow cascade reactions to occur. Some potential reactive electrophilic and nucleophilic sites in indigo **1** are highlighted in Figure 2. Given that the reaction of indigo **1** with bromoacetonitrile **13** gave poor yields, we anticipated that the less strongly electron withdrawing effects of the ester functionality versus the cyano functionality may promote better yields of cascade products due to less activated intermediates allowing more controlled cascade processes, although nucleophilic addition-elimination processes at the ester carbonyl might still proceed. Further, we planned to investigate analogous reactions using indirubin **1a** (Figure 2), which is known to undergo cascade reactions [12] and is less reactive than indigo **1**.

## 2. Results

### 2.1. Reaction of Indigo with Ethyl Bromoacetate

**Scheme 2:**
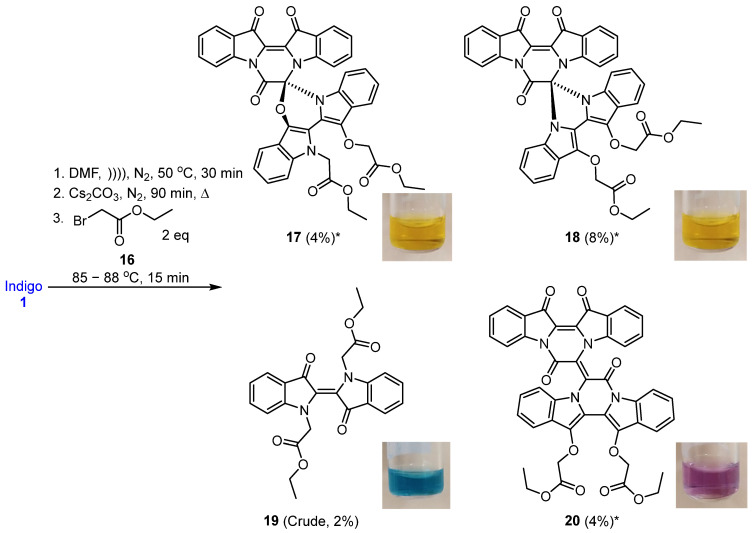
Base-promoted cascade reaction of indigo **1** with ethyl bromoacetate **16** to produce compounds **17**–**20**. Note: Relative configurations are shown for **17** and **18**. * X-ray crystal structures are available (see Figure 3).

In a typical reaction, a suspension of indigo **1** in anhydrous DMF was sonicated under a static nitrogen environment to facilitate maximum dissolution. The resulting suspension was cannulated into a reaction flask containing pre-dried caesium carbonate and 4 Å molecular sieves and was stirred under a nitrogen environment at 85–88 °C for 90 min. Ethyl bromoacetate **16** was added, and after 15 min the reaction was quenched, and the new heterocyclic systems **17**, **18,** and **20** were recovered, together with the *N*,*Nʹ*-disubstituted indigo derivative **19**, in low yields after silica gel column chromatography (Scheme 2).

**Figure 3:**
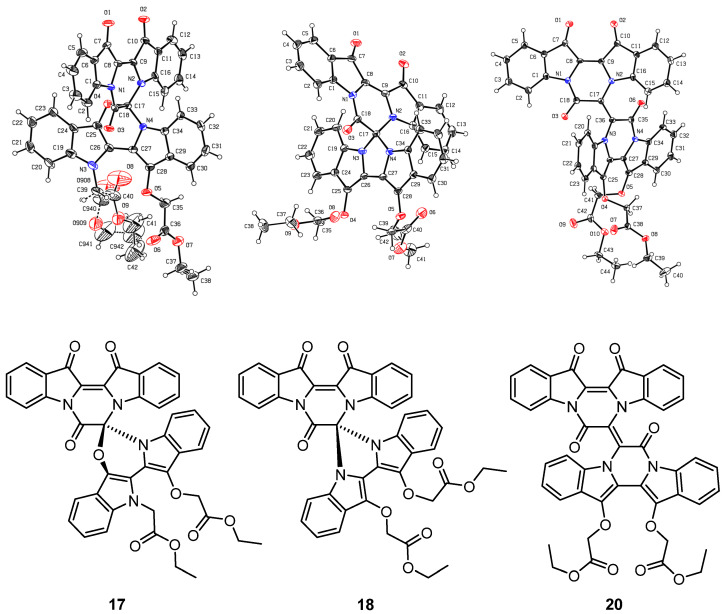
X-ray crystallographic structures of compounds **17**, **18**, and **20**. Note: Atom numbering in the X-ray structures is different from those used in the systematic numbering.

Analysis of the ^1^H NMR spectrum of compound **19** revealed only four aromatic resonances at δ 7.67, 7.63, 7.38, and 7.13, assigned to H4 and H4′, H6 and H6′, H7 and H7′ and H5 and H5′, respectively, which indicated a symmetrical structure. The ^1^H NMR resonances at δ 1.15, 4.08, and 4.92 were assigned to the *N*-ethoxycarbonylmethyl (CH_3_CH_2_OCOCH_2_) moieties as H5′′ and H5′′′, H4′′ and H4′′′ and H1′′ and H1′′′, respectively. The ^1^H NMR spectrum also indicated the presence of some minor impurities, and therefore a 2% crude yield is reported. The HRMS-ESI analysis of **19** revealed an ion peak at *m*/*z* 435.1572, assigned as the [M + H]^+^ ion for the molecular formula C_24_H_23_N_2_O_6_. In contrast, structures of the diindigoids **17**, **18**, and **20** structures were solved by X-ray crystallographic analysis (Figure 3). All three molecules consisted of a pyrazinodiindolinone bonded to a biindole through either the spirocyclic compounds **17** and **18** or a twisted unsaturated core, **20**.

**Figure 4:**
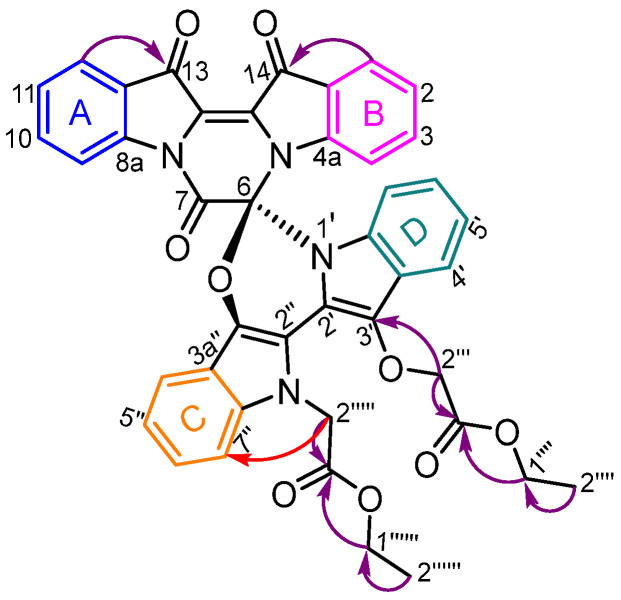
HMBC correlations in **17** annotated as purple and NOESY as red. COSY correlations with the four *ortho*-coupled aromatic systems annotated, as follows: (**A**, blue) 8.27 (H9)—7.61 (H10)—7.40 (H11)—7.99 (H12), (**B**, magenta) 7.91 (H1)—7.14 (H2)—7.31 (H3)—6.87 (H4), (**C**, orange) 7.49 (H4′′)—7.08 (H5′′)—7.25 (H6′′)—7.26 (H7′′) and (**D**, teal) 6.96 (H7′)—7.02 (H6′)—7.11 (H5′)—7.59 (H4′).

Diindigoid **17** was isolated as a yellow solid from which crystals were obtained by slow evaporation from CH_2_Cl_2_/hexane. HRMS-ESI spectrum analysis of the solids showed an ion peak at *m*/*z* 735.2086, assigned [M + H]^+^ for a molecular formula of C_42_H_31_N_4_O_9_. This suggested the presence of two moieties derived from indigo with three additional ethyl bromoacetate-derived units, with one of the units also involving loss of the EtO group. Analysis of the COSY spectrum indicated 4 distinct *ortho*-coupled proton networks, which suggested the presence of two non-symmetrical indigo cores (Figure 4 and Figure S3). Analysis of the ^1^H NMR spectrum revealed resonances at δ 1.29, 1.31, 4.25, 4.31, 4.97, and 5.54/5.75, which corresponded with two ethoxycarbonylmethyl substituents and were assigned as H2′′′′′′, H2′′′′, H1′′′′′′, H1′′′′, H2′′′, and H2′′′′a/H2′′′′b, respectively. Analysis of the HMBC spectrum (Figure S4) revealed correlations of ^1^H NMR resonances δ 7.99 (H12) and 7.91 (H1) with the ^13^C NMR resonances δ 178.6 (C13) and 180.4 (C14), respectively. The ester carbonyls were observed at δ 169.5 (C1′′′′′) and 168.6 (C1′′′), which correlated with a diastereotopic doublet δ 5.65 (H2′′′′′) and a singlet δ 4.97 (H2′′′), respectively. This assignment was supported by HMBC correlations of resonances δ 4.25 (H1′′′′′′) and 4.31 (H1′′′′) with δ 169.5 (C1′′′′′) and 168.6 (C1′′′), respectively. Analysis of the DEPTq_135_ spectrum revealed an additional ^13^C resonance downfield at 155.4 ppm, which corresponded to a quaternary carbon. The HMBC analysis indicated this resonance correlated with no ^1^H NMR resonances and it was therefore assigned to the carbonyl C7. Analysis of the NOESY spectrum indicated the diastereotopic pair of doublets at δ 5.65 correlated with the aromatic δ 7.26 resonance. Given the diastereotopic splitting is typically observed with sterically hindered CH_2_ *N*-substitutions, the 5.65 and 7.26 ppm resonances were assigned to H2′′′′′ and H7′′, respectively. The other CH_2_ resonance at 4.97 ppm was a singlet and therefore possesses a greater degree of freedom, which is typical of an *O*-substitution. By HMBC spectral analysis, the ^1^H resonance at 4.97 ppm correlated with a ^13^C NMR resonance at 133.3 ppm, which in turn correlated with the ^1^H NMR resonance at δ 7.59 and these were assigned to H2′′′, C3′, and H4′, respectively. The indolinones of the pyrazinoindolotrione half of the molecule were distinguished based on differences in chemical shifts. It was proposed the C7-N8 amide would withdraw the free electron pair on N8, reducing its capacity to resonate with the aromatic ring, i.e., resulting in upfield chemical shifts. Subsequently, analysis of the DEPTq_135_ spectrum indicated resonances at δ 144.4 and 148.0, which were assigned to C8a and C4a, respectively.

Diindigoid **18** was isolated as a yellow amorphous solid and eluted with a similar HPLC retention time to **17**. A molecular formula of C_42_H_30_N_4_O_9_Na was assigned to the [M + Na]^+^ ion peak at *m*/*z* 757.1927^+^ by analysis of the HRMS-ESI spectrum. Analysis of the ^1^H NMR spectrum revealed 16 aromatic protons with 4 resonances that integrated to 2, which indicated one indigo-derived unit was symmetrical. Only one set of CH_2_CO_2_C_2_H_5_ group resonances were observed with the quartet at δ 4.31 (H1′′′′) and triplet at δ 1.31 (H2′′′′), which integrated for 10 H in agreement with a symmetrical indigo moiety being present. Analysis of the DEPTq_135_ spectrum indicated a ^13^C NMR resonance at 155.1 ppm, which had no HMBC correlations, and this was assigned to C7, similar to the assignment of diindigoid **17** (Figure S7). The presence of the diastereotopic pair of doublets at δ 5.20 (H2′′′) suggested the ethyl acetate moieties were *N*-linked to the indigo core; however, the HMBC spectrum analysis indicated a single correlation with the ^13^C NMR resonance at δ 132.9 (C3′), which correlated with the ^1^H NMR resonance δ 7.72 (H4′). Therefore, the ethyl acetate moieties were proposed to be attached via *O*-alkylation. This was confirmed by observation of the correlation between δ 7.72 and 5.20 in the NOESY spectrum, which were assigned H4′ and H2′′, respectively. Therefore, structure **18** was proposed as a structural isomer of **17** with the indigoids linked via an imidazole-derived ring.

The purple colour of diindigoid **20** suggested extended conjugation was present in the molecule. The HRMS-ESI analysis indicated an ion peak at *m*/*z* 797.1859^+^ assigned to [M + Na]^+^ for a molecular formula of C_44_H_30_N_4_O_10_Na, which corresponded to the presence of two indigo units with two CH_2_CO_2_C_2_H_5_ moieties plus an additional C_4_O_2_ unit. Analysis of the ^1^H NMR spectrum revealed resonances of two ethyl moieties at δ 1.29, 1.32 and 4.24, which were assigned to H2′′′′′, H2′′′ and H1′′′′′/H1′′′, respectively. Two diastereotopic doublets were observed at δ 4.93 and 4.83 and were assigned H2′′ and H2′′′′, respectively. Analysis of the HMBC spectrum revealed the ester carbonyls at δ 169.1 (C1′′ and C1′′′′) correlated with the diastereotopic doublets at δ 4.93 and 4.83 (H2′′/H2′′′′), which themselves correlated with ^13^C resonances at δ 139.4 and 139.2, respectively. These ^13^C resonances also correlated with the ^1^H resonances at δ 7.89 and 7.76 and were therefore assigned to C14′, C13′, H1′ and H12′, respectively. Analysis of the DEPTq135 indicated two quaternary carbons at δ 153.6 and 154.8, which were assigned to C7′ and C7, respectively. The presence of these two carbonyls and no additional protons suggested the formation of two pyrazinone rings, which were fused via a tetrasubstituted double bond. No heteronuclear correlations were observed to enable confirmation of the stereochemistry of the double bond in view of the nature of the substituents present. The *E*-stereochemistry was established from X-ray crystallography (Figure 3) in which unfavourable interactions between the two carbonyl groups would be obviated.

### 2.2. Investigation of the Effect of Reaction Conditions on Product Outcomes for the Indigo and Ethyl Bromoacetate Reaction

**Scheme 3:**
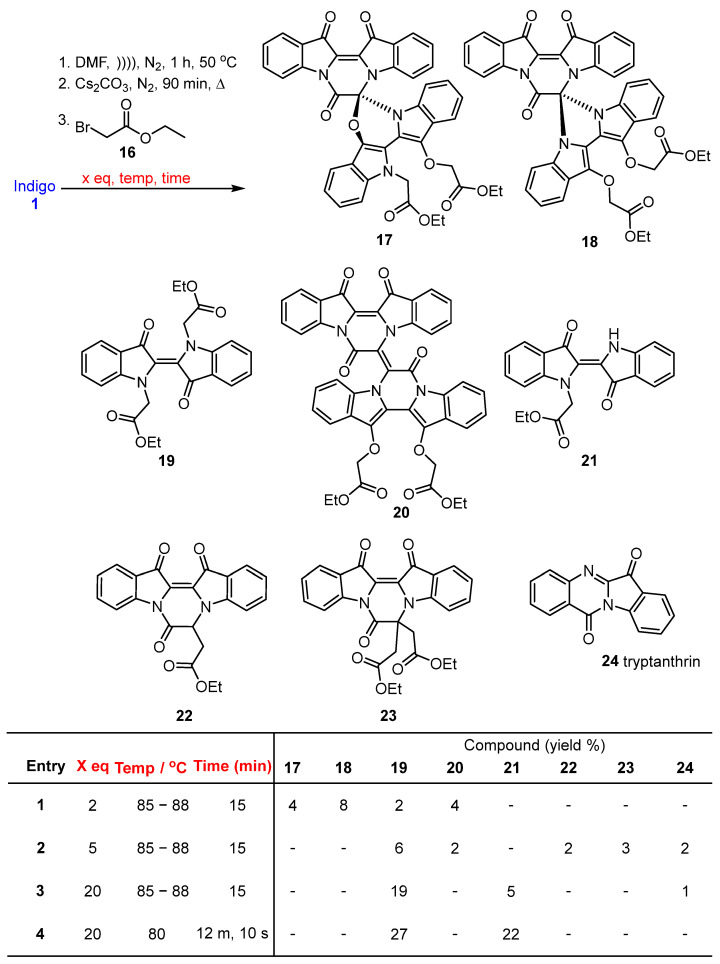
Optimisation of the reaction of indigo **1** with ethyl bromoacetate **16**.

The reaction of indigo **1** with ethyl bromoacetate **16** resulted in the formation of the structurally notable compounds **17**, **18**, and **20**, plus **19**, albeit in low yield (Scheme 3, entry 1). This outcome strongly indicated that ethyl bromoacetate **16** is highly reactive, resulting in cascade pathways incorporating alkylation, cyclization, and coupling reactions. It was thought that the poor yield was mainly due to the low amount of electrophile (2 eq) used in the initial reaction studies, which activated the reactive -CH_2_ group quickly, leading to the diverse array of pathways in the reaction. Therefore, the reaction of indigo **1** with ethyl bromoacetate **16** was re-investigated, changing the number of equivalents of electrophile **16** used, the reaction temperature, and the reaction time (Scheme 3, entries 2–4). Thus, to a stirred mixture of indigo **1**, anhydrous caesium carbonate, and 4 Å molecular sieves in DMF under a nitrogen atmosphere, ethyl bromoacetate **16** (either 5 or 20 equivalents) was added to the reaction mixture for 15 or near 12 min of reaction time. Subsequent workup and multiple rounds of silica gel column chromatography afforded a number of products, including the *N*,*N*′-dialkylated product **19** and the coupled product **20**, although not compounds **17** or **18**. (Scheme 3, entries 2–4). The addition of 5 equivalents of **16** for 15 min of reaction time yielded *N*,*N*-dialkylated indigo **19** (6%), biindigoid **20** (2%), cyclised compounds **22** (2%), and **23** (3%) along with the known product tryptanthrin **24** [13,14] (2%) and a non-separable mixture of other compounds (Scheme 3, entry 2). The fused pyrazino derivatives **22** and **23** had not been isolated previously. Compound **19** was also isolated pure (6%), in contrast to previous attempts. Analysis of the ^1^H NMR spectrum of compound **22** revealed eight aromatic resonances δ 8.49, 7.94, 7.85, 7.69, 7.60, 7.38, 7.15, and 7.04, assigned to H4, H1, H12, H3, H10, H2, H11, and H9, respectively. This indicated the presence of the indigo core within the molecule. Additionally, in the upfield region of the spectrum, four resonances at δ 5.17, 4.01, 3.31, and 1.06 were assigned to H6, H1′′, H2′, and H2′′′. HMBC spectral analysis showed a resonance at 5.17 ppm correlated to 160.6, 168.8, 126.1, and was assigned to C7, C1′ and C13b, respectively (Figure S29). ^13^C NMR analysis further supported these findings by revealing four characteristic resonances at 180.3, 178.4, 168.8, and 160.6 ppm, which were assigned to C13, C14, C1′, and C7, respectively. HRMS-ESI spectral analysis of compound **22** revealed an ion peak at *m*/*z* 411.0959^+^, which was assigned to the [M + Na]^+^ ion for the molecular formula C_22_H_16_N_2_O_5_Na. The HRMS-ESI spectrum of compound **23** gave an ion peak at *m*/*z* 475.1505, which was assigned to [M + H]^+^ ion for the molecular formula of C_26_H_23_N_2_O_7_, consistent with the presence of the indigo core and two additional ethyl acetate-derived moieties.

Analysis of the ^1^H NMR spectrum of compound **23** showed eight aromatic resonances and two sets of ethyl acetate resonances with cumulative integrations of 14 protons. Among these, a quartet at δ 3.92 in ^1^H NMR spectrum was assigned as H1′′ and H1′′′′, which correlated to the resonances at δ 167.9 (C1′ and C1′′′), and 13.8 (C2′′ and C2′′′′), respectively, in the HMBC spectrum. A doublet of doublets at δ 3.57 in the ^1^H NMR spectrum was assigned to H2′ and H2′′′, correlating to the resonances at δ 163.8 (C7), 167.9 (C1′ and C1′′′), 61.5 (C1′′ and C1′′′′). and 40.8 (C2′ and C2′′′), respectively, in the HMBC spectrum. A triplet δ 0.97 assigned to H2′′ and H2′′′′, correlated to a resonance at δ 13.8 and ^13^C were attributed as C2′′ and C2′′′′.

The biindigoid **20** was isolated in 2% yield, and trypanthrin **24** (2%) was identified as a reaction disintegration product, with NMR data in agreement with that previously reported [13].

Scheme 3, entry 3, with the addition of 20 equivalents of electrophile **16** in the reaction mixture for 15 min at 85–88 °C, gave *N*,*N*′-dialkylated indigo **19** (19%), *N*-alkylated indigo **21** (5%), and tryptanthrin **24** (1%) along with a mixture of minor products and baseline material. With 20 equivalents of electrophile **16**, a reaction temperature slightly reduced (from 88 to 80 °C) and a reaction time shortened to just over 12 min, two major products were isolated, two with improved yields: *N*,*N*′-dialkylated indigo **19** (22%), and *N*-alkylated indigo **21** (27%) (Scheme 3, entry 4). Furthermore, there was evidence of baseline polymeric material, likely arising from the formation of undesired minor products or disintegration of the starting materials. It is of note here that previous work by others on the reaction of indigo with *tert*-butyl bromoacetate (in DMF with Cs_2_CO_3_ as a base in a sealed vial) at 23 °C over 24 h gave a moderately good yield (66%) of the corresponding (*E*)-*N*,*N*′-dialkylation product, *N*,*N*′-*bis*(*tert*-butyloxycarbonylmethyl)indigo, and no other reaction products were reported [15]. The same low-temperature method was used again more recently to make this dialkylation product [16].

### 2.3. Reaction of Indirubin with Ethyl Bromoacetate

Indirubin **1a** (Figure 2), a naturally occurring regioisomer of indigo **1**, is renowned for its range of biological activities and has been employed in traditional Chinese medicine for anticancer treatment [17,18,19,20,21]. Many derivatives of indirubin have been made in connection with probing their anti-tumour properties, including the development of the first hybrid dual inhibitors [22] of glutathione peroxidase 4 (GPX4) and cyclin-dependent kinase (CDK), as well as molecules inducing damage to DNA and targeting the poly (ADP-ribose) polymerases (PARPs) involved in DNA repair [23]. Beyond its significance in the realm of biology and medicine, indirubin **1a** possesses interesting chemical properties [12,24,25]. Notably, in our previous work [12], it was found that indirubim is less reactive than indigo, and it was reasoned that this might impart greater control in reactions with ethyl bromoacetate. Therefore, indirubin **1a** was selected for further investigation with ethyl bromoacetate **16** in an attempt to invoke polyheterocycle formation via cascade reactions in a controlled manner.

**Scheme 4:**
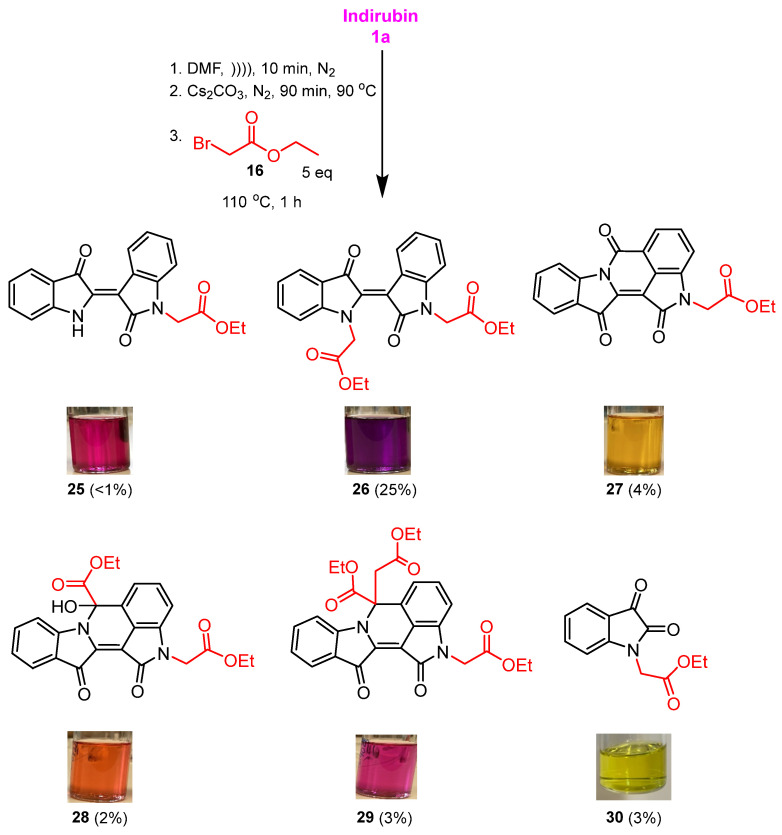
Base-promoted cascade reaction of indirubin **1a** with **16** to produce polyheterocyles **25**–**29**, plus the *N*-substituted isatin **30**.

In this context, indirubin **1a** was reacted with **16** using conditions that were previously optimised when allyl bromide was used as the electrophile [12]. Therefore, indirubin **1a** was dissolved in DMF and subjected to sonication for 10 min under a static nitrogen environment. The resulting suspension was cannulated into a reaction flask containing pre-dried caesium carbonate and stirred vigorously for 90 min at 90 °C. Subsequently, the temperature was reduced to 80 °C, and ethyl bromoacetate **16** was added over 30 min at this reaction temperature. Upon workup and subsequent purification by silica gel column chromatography, the monosubstituted *N*-alkylated indirubin **25** (48%), and the disubstituted *N*,*N*′-dialkylated indirubin **26** (20%) were isolated as dark pink and dark purple fluffy solids, respectively, along with minor products. In an attempt to invoke the cyclisation of these molecules, subsequent reactions were performed at a higher temperature (110 °C) and the addition of the electrophile **16** over an extended reaction time of 1 h. This afforded sets of polyheterocyles: *N*-alkylated indirubin **25** (<1%), *N*,*N*′-dialkylated indirubin **26** (25%), compound **27** (4%), compound **28** (2%), compound **29** (3%), and the degradation product **30** (3%) (Scheme 4). Compounds **27**, **28,** and **29** are derivatives of a previously undescribed fused indirubin skeleton, the indolo[1,2-*b*]pyrrolo[4,3,2-*de*]isoqinoline system.

Analysis of the HRMS-ESI spectrum of compound **27** revealed an ion peak at *m*/*z* 397.0815, which was assigned as the [M + Na]^+^ ion for the molecular formula of C_22_H_10_N_6_O_5_Na and corresponded to the presence of the indirubin core and one CH_2_CO_2_C_2_H_5_ moiety. Analysis of the ^1^H NMR spectrum showed seven resonances at δ 8.81, 7.92, 7.87, 7.75, 7.57, 7.40, and 6.99 integrating to seven protons and assigned as H8, H11, H5, H9, H4, H10, and H13, respectively. The *N*-ethyl acetate moiety resonances were observed at δ 4.62, 4.25, and 1.29 and assigned to H1′, H4′, and H5′, respectively. Analysis of the ^13^C NMR spectrum of **27** showed significant resonances at δ 180.8, and 162.2, assigned C12, and C1 carbonyls, whereas the ^13^C resonances at δ 159.0, and 167.4, were assigned to C6, and C2′, corresponding to the amide and ester carbonyls, respectively. Analysis of the HMBC spectrum further supported these correlations by showing the resonances of H11 at δ 180.8 (C12), H1′ at δ 162.2, and 167.4 (C1, C2′), and H5 at δ 159.0 (C6), respectively.

Analysis of the HRMS-ESI spectrum of compound **28** revealed an ion peak at *m*/*z* 471.1180, which was assigned as [M + Na]^+^ ion for the molecular formula of C_25_H_16_N_6_O_3_Na, and indicated the presence of the indirubin core and two ethyl acetate moieties with loss of a CH_2_ group but with the addition of an OH group. Analysis of the ^1^H NMR spectrum of compound **28** displayed seven aromatic resonances at δ 7.67, 7.52, 7.33, 7.22, 7.11, 7.08, and 6.99, assigned to H11, H9, H4, H8, H5, H10, and H3, respectively. The *N*-ethyl acetate moiety resonances were observed at δ 4.31, 4.28–4.15, and 1.08 in the ^1^H NMR spectrum were assigned as H2′, H1′′, and H2′′. Analysis of the ^1^H NMR spectrum further showed other resonances which could be assigned as follows: at δ 5.39 (OH), 4.43 (H1′′′) and 1.26 (H2′′′). Analysis of the ^13^C NMR spectrum revealed resonances at δ 181.8, and 162.9, assigned to carbonyls C12, and C1, and two ^13^C resonances at δ 169.2, and 167.8, assigned to the ester carbonyls as C1′′′, and C1′, respectively. Analysis of the HMBC spectrum displayed the correlations of the OH to a resonance at δ 169.2 (C1′′′), H11 to δ 181.8 (C12), H2′ to δ 162.8 (C1) and 167.8 (C1′), and H5 to δ 66.2 (C6). In contrast to compound **28**, analysis of the ^1^H NMR spectrum of compound **29** displayed a similar trend with the exception of an additional ethyl ester moiety in the upfield region of the spectrum. Interestingly, the absence of a resonance for the OH moiety in the ^1^H NMR spectrum of **29** suggested that the additional ethoxycarbonylmethyl moiety was replaced with an OH group at the C-6 position. This conclusion was supported by the HMBC analysis, which showed the correlations between the additional moiety’s diastereotopic proton H2′′′′′_ab_ to a ^13^C resonances at δ 169.0, 168.0 and 126.0 ppm, corresponding to the carbons as C1′′′, C1′′′′′, and C5a respectively. Furthermore, the HMBC analysis indicated that the quartet at δ 3.81, assigned to H1′′′′′′ correlated to the resonances at δ 168.0 and 13.6 ppm, leading to the assignments C1′′′′′, and C2′′′′′′, respectively (Figure S59). The HRMS-ESI spectrum of compound **29** revealed an ion peak at *m*/*z* 519.1767, which was assigned as [M + Na]^+^ ion for the molecular formula of C_28_H_26_N_2_O_8_Na (Figures S56–S60).

**Scheme 5:**
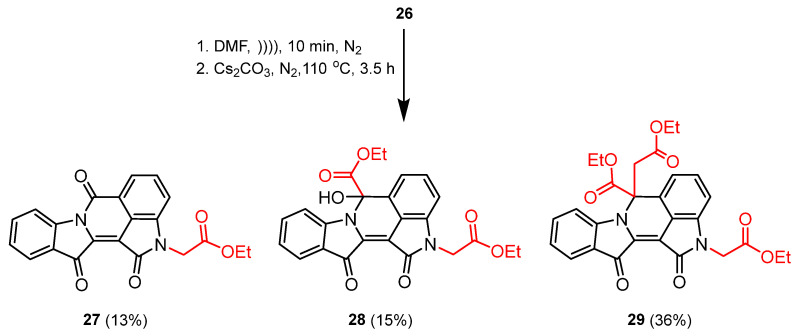
Reaction of *N*,*N*′-dialkylated indirubin **26** in the presence of a base to yield compounds **27**–**29**.

Based on the cascade reaction of indirubin **1a**, the product outcomes indicated that *N*,*N*′-dialkylated indirubin **26** could potentially serve as the starting material for the formation of cascade products **27**–**29**. To understand the reaction mechanism and improve the product yields, the study was extended to include some control mechanism experiments. Therefore, *N*,*N*′-dialkylated indirubin **26** was synthesised according to previously optimised reaction conditions. This compound **26** was then dissolved in DMF with ultrasonication, two equivalents of anhydrous caesium carbonate were added, and the mixture was stirred vigorously at 110 °C under a nitrogen environment. Notably, no ester **16** was added to the reaction mixture. After a reaction time of 3.5 h, crushed ice was added, and subsequent work-up, including multiple rounds of preparative TLC, yielded the compounds **27**–**29** with improved yields (Scheme 5).

### 2.4. Mechanistic Discussion of the Indigo-Ethyl Bromoacetate Reactions

**Scheme 6:**
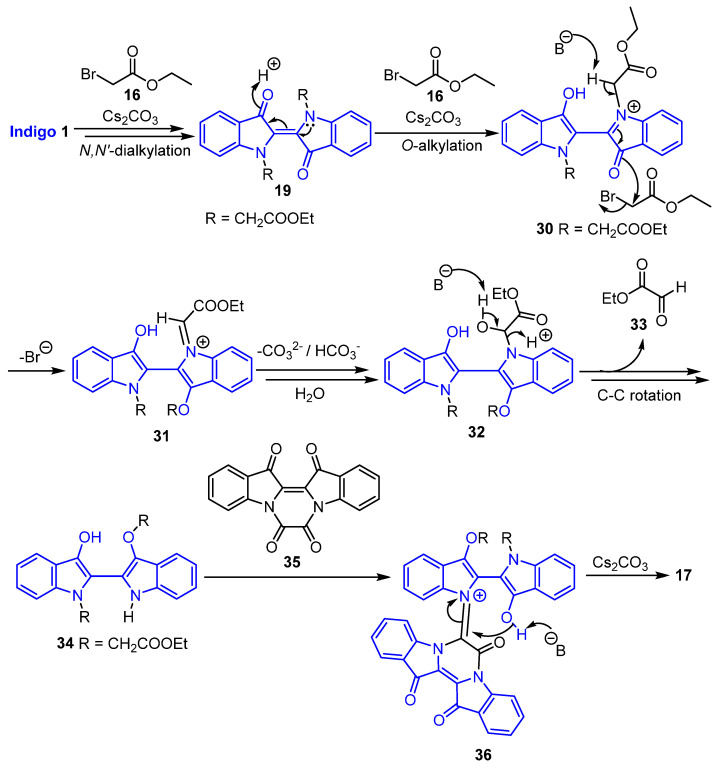
Proposed mechanism for the formation of the spirocycle **17**.

**Scheme 7:**
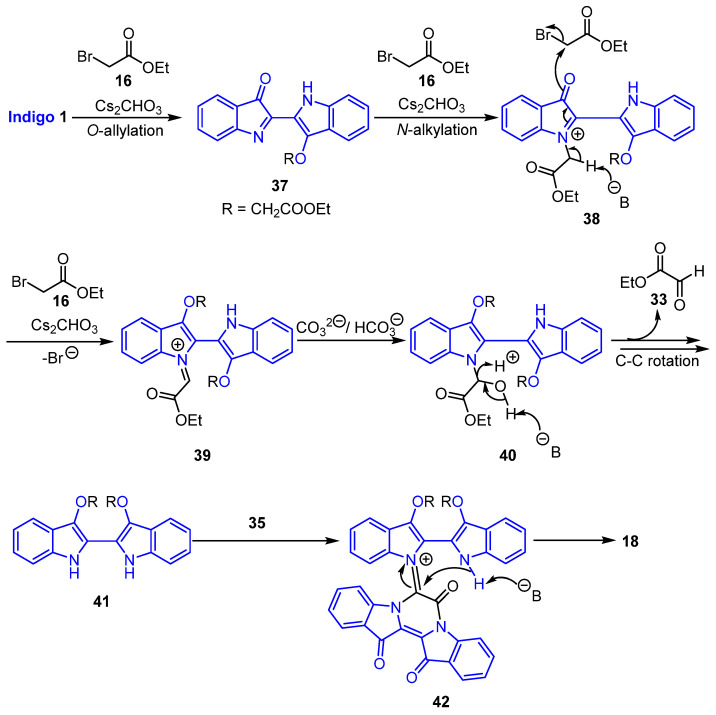
Proposed mechanism for the formation of the spirocyclic aminal **18**.

**Scheme 8:**
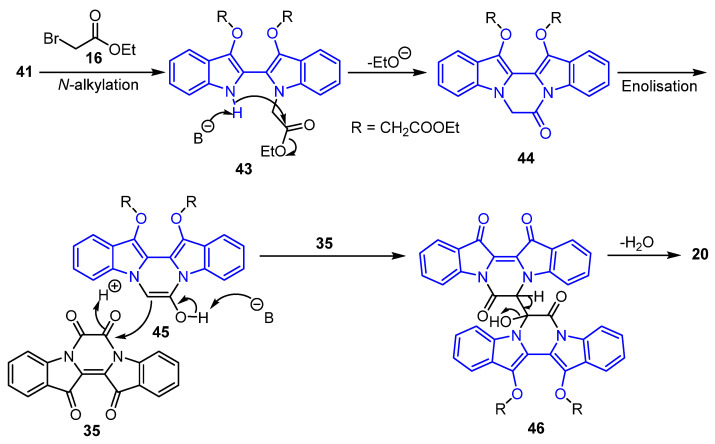
Proposed reaction mechanism for the formation of the double bond-linked compound **20**.

**Scheme 9:**
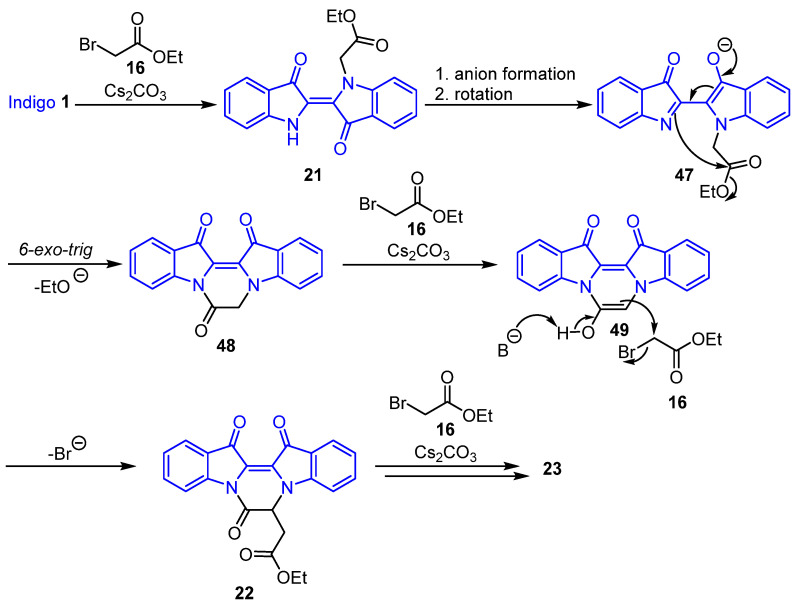
Proposed reaction mechanism for the formation of compounds **22**–**23** via *N*-alkylation of indigo **1**.

**Scheme 10:**
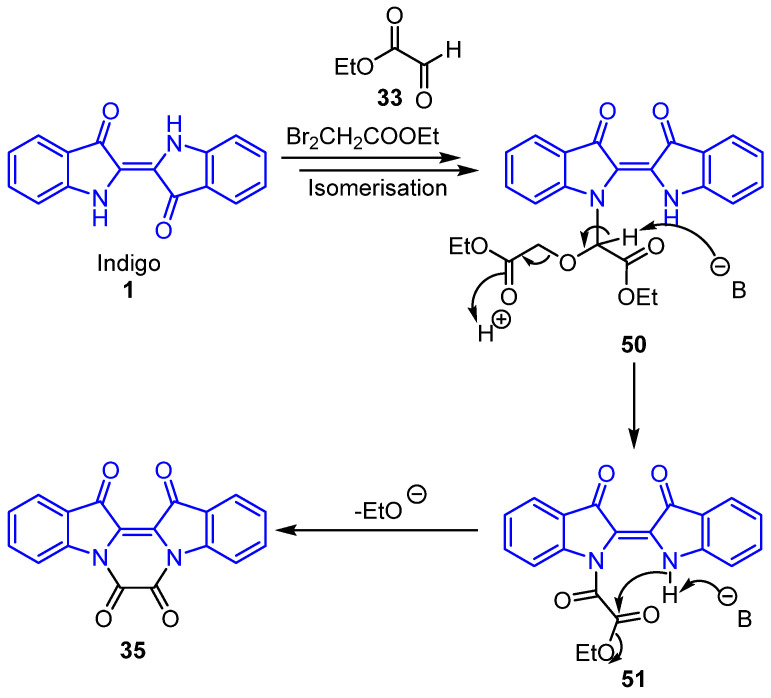
Formation of proposed intermediate **35**. Ethyl glyoxylate **33** may be generated in situ as noted in Scheme 6 and Scheme 7, or possibly from a carbonate ester intermediate (see Supplementary Materials, Scheme S1).

The proposed reaction mechanisms for the formation of the cascade products **17**, **18**, and **20**–**23** are summarised in Scheme 6, Scheme 7, Scheme 8 and Scheme 9. For the synthesis of **17**, the first step is the *N*,*N*′-dialkylation of indigo **1** leading to the formation of intermediate **19** (Scheme 6). Subsequent *O*-alkylation could then give rise to intermediate **30**. Base removal of the acidic methylene hydrogen would then give rise to iminium-based intermediate **31**. In the presence of bicarbonate and water, intermediate **31** could be converted to **32**. The base-induced removal of glyoxylate **33** would then furnish *O*,*O*′-dialkylindigo **34** after C-C bond rotation. The condensation reaction of *O*,*O*′-dialkylindigo **34** with oxalylindigo **35** produced in situ (see Scheme 10 for a proposed route to this known [26] compound under the reaction conditions) would then afford access to intermediate **36**. The subsequent 6-*endo*-*trig* cyclisation of intermediate **36** in the presence of base would afford compound **17** (Scheme 6).

The proposed mechanism of formation of compound **18** is described in Scheme 7 and begins with the reaction of indigo **1** with **16** in the presence of a base to generate *O*-alkylindigo **37**. Subsequent *N*-alkylation could then lead to intermediate **38** with subsequent base removal of an acidic methylene hydrogen giving rise to the iminium-based intermediate **39**. In the presence of bicarbonate, intermediate **39** could be converted to **40**, followed by base-initiated removal of glyoxylate **33** and C-C bond rotation to access *O*,*O*′-dialkylindigo **41** with bicarbonate also acting as a proton source. The reaction of **41** with **35** could give an intermediate **42**. Base removal of the acidic methylene proton of **42** and subsequent cyclisation would then result in compound **18** (Scheme 7).

The proposed mechanism for the formation of compound **20** is suggested, to begin with the *N*-alkylation of branch point intermediate **41** in the presence of base and ethyl bromoacetate **16** to produce **43,** which undergoes cyclisation in the presence of a base to generate lactam intermediate **44**. The lactam **44** could undergo enolization, and a subsequent reaction with intermediate **35** in the presence of base would furnish intermediate **46**. Finally, dehydration of intermediate **46** could give compound **20** (Scheme 8).

The proposed mechanism of formation for the cascade products **22** and **23** arises from the *N*-alkylation of indigo **1** producing **21**, with subsequent anion formation and rotation giving rise to the intermediate **47**. After a *6-exo-trig* cyclisation step followed by the elimination of ethoxide, the reaction would furnish lactam **48**. Alkylation of the carbonyl α-position with **16** as the electrophile produces the product **22**. Further C-alkylation would afford **23** (Scheme 9).

The reaction between indigo **1** and ethyl bromoacetate **16** resulted in the formation of a wide range of polyheterocycles **17**–**23**. Notable key intermediates, e.g., oxalylindigo **35** and lactam **48**, are critical for the construction of the more complex systems and derivatives and could be formed in situ during the reaction but were not isolated during the optimisation process. The suggested reaction mechanism for the formation of **48** is detailed in Scheme 9, while the proposed mechanism for the formation of intermediate **35** is illustrated in Scheme 10. This involves the reaction of indigo **1** with ethyl bromoacetate **16** in the presence of ethyl glyoxylate **33** and base to produce intermediate **50** after isomerisation about the C=C bond (Scheme 10). Subsequent base removal of the acidic proton at the ester carbonyl α-position may follow, which would then allow for the elimination of ethyl acetate and the formation of intermediate **51**. Base removal of the proton of the *-NH* group of **51** and subsequent cyclisation at the adjacent carbonyl with ethoxide ion loss would result in oxalylindigo **35** (Scheme 10).

### 2.5. Mechanistic Discussion of the Indirubin-Ethyl Bromoacetate Reaction

**Scheme 11:**
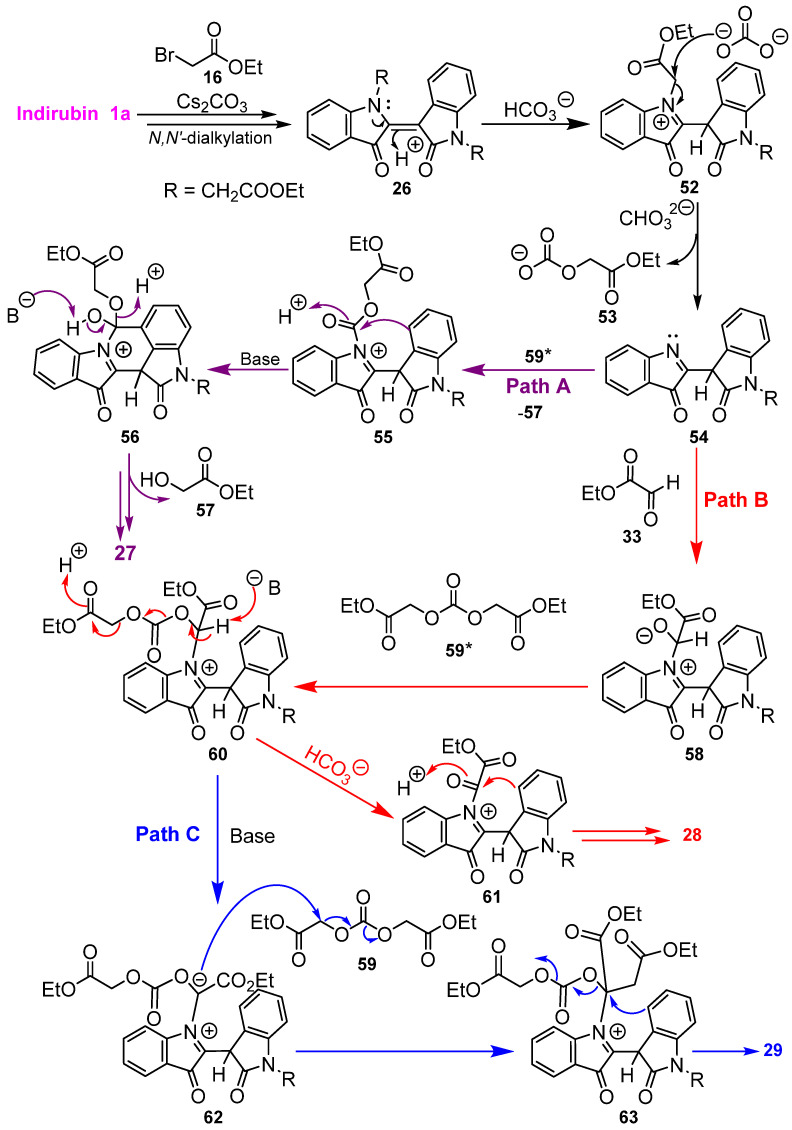
Proposed reaction mechanisms for the formation of compounds **26**–**29** from cascade reaction of indirubin **1a** with ethyl bromoacetate **16**. * For the proposed synthesis in situ synthesis of **59**, see the Supplementary Materials (Scheme S1).

The proposed mechanism for the formation of compounds **25**–**29** is illustrated in Scheme 11. The initial steps involve indirubin *N*,*N*′-dialkylation in the presence of Cs_2_CO_3_ with ethyl bromoacetate **16**, resulting in the production of *N*,*N*′-dialkylated indirubin **26,** probably via the *N*-alkylated compound **25**. Nucleophilic attack by the carbonate ion at the electron-deficient α-position to the ester carbonyl with loss of the carbonate **53** (an in situ precursor of *bis*(ethoxycarbonylmethyl)carbonate **59**; see Scheme S1) would then provide access to the key imine intermediate **54**. Some **54** might also result more directly from the *N*-monosubstituted indirubin **25**. The imine **54** could then serve as a point of divergence in pathways. In **Path A**, intermediate **54** could react with the *bis*-carbonate **59** to generate **55**. Subsequent aromatic electrophilic substitution of **55** to give intermediate **56**, followed by elimination of ethyl glycolate **57**, would ultimately yield the isolated derivative **27**.

In **Path B**, the imine intermediate **54** could add to the aldehyde group in ethyl glyoxylate **33** to produce intermediate **58**, which in turn could participate in a nucleophilic substitution reaction with the *bis*-carbonate **59** giving rise to **60**. Intermediate **60** could then proceed to the isolated cyclised compound **28** by subsequent steps, first involving conversion to **61** (with the elimination of ethyl acetate and carbon dioxide) and then intramolecular electrophilic aromatic substitution. The formation of the unsymmetrically *gem*-disubstituted derivative **29** may also possibly arise from the intermediate **60** (**Path C**) in an alternative pathway via deprotonation to produce **62,** which could react with the carbonate **59** or possibly with ethyl bromoacetate **16**, leading to the formation of **63**. Finally, intramolecular aromatic electrophilic substitution and proton loss steps in the presence of base would afford compound **29**. The fact that the disubstituted compound **26** could be converted independently to the cyclized products **27**–**29** on heating at a higher temperature with Cs_2_CO_3_ in DMF is consistent with the proposed mechanistic pathways A–C with the plausible presumption that both the other reactants **59** and **33** could still be generated in situ. The carbonate ion **53** could possibly compete as a nucleophile in the step from **52** to **54** in Scheme 11, thus giving rise also to **59,** which in turn could act as a precursor of glyoxylic ester **33** (Scheme S1).

## 3. Materials and Methods

### 3.1. Chemistry

Anhydrous dimethylformamide (DMF) was purchased from Merck (Sigma-Aldrich, St. Louis, MO, USA), and used without further purification. High-performance liquid chromatography (HPLC) grade dichloromethane (CH_2_Cl_2_) was used, and all other solvents were purchased reagent grade and used without further purification unless otherwise stated. Indigo was purchased from Merck (St. Louis, MO, USA) and AK Scientific (Union City, CA, USA) and used without further purification. Indirubin was synthesised as reported [27]. Ethyl bromoacetate was purchased from Sigma-Aldrich. Deionised (reverse osmosis; RO) water was used for extractions and preparation of aqueous solutions and was obtained from a Millipore purification system. Salt solutions such as brine or NaHCO_3_ were prepared from the commercially available salt and are saturated aqueous solutions unless specified otherwise. Nitrogen used in reactions was passed through a 20 cm tube filled with a silica-based drying agent (blue→yellow indicator) or anhydrous calcium chloride. Cs_2_CO_3_ was stored in a desiccator and pre-dried by heating at 85–87 °C for an hour under a high vacuum. Molecular sieves (4 Å) were activated in a furnace at 300 °C overnight before use. Melting point temperatures are expressed in degrees Celsius (°C) and are uncorrected. ^1^H and ^13^C NMR spectra (CDCl_3_) were recorded either at 500 and 125 MHz (Bruker Avance 500), or 400 and 100 MHz (Bruker Avance 400), respectively, with chemical shifts (δ) reported as parts per million relative to TMS (δ = 0.00 ppm) or CDCl_3_ (δ = 7.26; 77.0 ppm); the numbering used for resonance assignments may be different from the systematic numbering. Coupling constants (*J*) are reported in Hertz (Hz). Multiplicities are reported as singlets (s), doublets (d), triplets (t), doublet of doublets (dd), quartets (q), sextets (s), heptets (hept), or multiplets (m). Electrospray (ESI single quadrupole, Shimadzu LCMS-2020) mass spectra are reported as ion mass to charge values (*m*/*z*), with the relative abundances as a percentage in parentheses. Infrared (IR) spectra were recorded neatly on a Bruker Vertex 70 FTIR (Bruker Optik GmbH, Ettlingen, Germany). UV−visible absorption spectra were recorded in dichloromethane solutions at room temperature using a double-beam spectrophotometer. Thin-layer chromatography (TLC) was performed using silica gel F254 coated with aluminium sheets. Preparative thin-layer chromatography (PTLC) was performed. Column chromatography was performed using silica gel 60 (0.063−0.200 mm). Eluents are reported in volume-to-volume (*v*/*v*) ratios. Solvent extracts and chromatographic fractions were concentrated by rotary evaporation in vacuo. PS 40–60 refers to petroleum spirit, bp range 40–60 °C.

### 3.2. Reactions of Indigo with Ethyl Bromoacetate

#### 3.2.1. Method 1

Anhydrous DMF (40 mL) was added via cannula to a flask containing indigo (263.1 mg, 1.003 mmol) under nitrogen flow. The resulting suspension was sonicated for 30 min at 50 °C under a static N_2_ atmosphere. The solution was transferred via cannula to a flask containing pre-dried Cs_2_CO_3_ (1.321 g, 4.055 mmol) and stirred at 85–88 °C under a static N_2_ atmosphere for 90 min. Ethyl bromoacetate **16** (0.22 mL, 2.0 mmol) was added via syringe, and the reaction was left to stir for 15 min at 85–88 °C and then quenched in ice water. The organic material was extracted with EtOAc (1 × 40 mL, 3 × 20 mL) and then washed with water (3 × 50 mL) and brine (1 × 50 mL). The organic layer was dried (MgSO_4_), concentrated in vacuo, and the residue was subjected to silica gel column chromatography (40 g, 20% P.S/CH_2_Cl_2_ to 2% MeOH/EtOAc), which afforded a major yellow band (98.1 mg), a major purple band (32.5 mg), and significant decomposition (195.8 mg). The yellow band was subjected to further silica gel column chromatography (16 g silica, 30% EtOAc/PS 40–60 to 40% EtOAc/PS 40–60), which yielded a mixture of two yellow compounds (48.9 mg) that could be separated using preparative HPLC (15 mL.min^−1^, 70% MeCN/H_2_O with 0.1% TFA) to afford the compounds **17**–**18**. The major purple band was purified by silica gel column chromatography (13 g silica, 30% to 40% EtOAc/PS 40–60) to yield compound **19** (9.6 mg, crude yield 2%) and a major purple fraction that was further purified by recrystallization (slow evaporation from 1:1 CH_2_Cl_2_/hexane) to yield compound **20** (18.9 mg, 0.024 mmol, 4%).

#### 3.2.2. Method 2

Indigo (262.27 mg, 1.00 mmol) was dissolved in anhydrous DMF (40 mL), and the resulting suspension was sonicated for 1 h at 50 °C under a static N_2_ atmosphere. The solution was transferred to a flask containing pre-dried Cs_2_CO_3_ (1.303 g, 4.00 mmol) with activated 4 Å molecular sieves and stirred at 85–88 °C under a static N_2_ atmosphere for 90 min. Ethyl bromoacetate **16** (0.55 mL, 5 mmol) was added via syringe. The reaction mixture was stirred vigorously for 15 min at 85–88 °C under a static N_2_ atmosphere. The reaction mixture was then quenched by pouring into crushed ice (200 mL) and transferred to a separatory funnel. The aqueous layer was extracted with ethyl acetate (3 × 40 mL). The combined organic layers were washed with H_2_O (1 × 30 mL), brine (1 × 30 mL), dried (MgSO_4_) and concentrated in vacuo. The mixture was adsorbed on silica and loaded to a silica gel (100 g) column and elution with (100% hexane → 10% EtOAc/hexane → 2% MeOH/DCM) resulted in three major fractions (F_1_, F_2_, and F_3_), including minor fractions (F_4_ and F_5_), and baseline decomposition. Fraction F_1_ was identified as a disintegration product tryptanthrine **24** as a yellow waxy solid. Fraction F_2_ was subjected to silica gel (30 g) column chromatography, and the mixture was eluted with (100% hexane → EtOAc 10% to 20%) to give a partially pure compound **19** as a dark blue waxy solid. Further purification by multiple rounds of PTLC (1:9 EtOAc:hexane) gave a pure form of compound **19** as a dark blue waxy solid. Compound **22** was isolated as dark pink powder by fractional recrystallization (1:5 EtOAc:hexane) of fraction F_3_. The remaining aliquot of the recrystallisation was dried and adsorbed on PTLC using 3% MeCN in DCM, yielding compound **23** as a red, pink waxy solid. Fractions F_4_ and F_5_ were attempted to be purified either with silica gel column chromatography or multiple rounds of PTLC, resulting in the collection of six minor fractions, from which compound **20** was isolated as a dark purple waxy solid as a partially pure compound.

#### 3.2.3. Method 3

Indigo (262.27 mg, 1.00 mmol) was dissolved in anhydrous DMF (40 mL), and the resulting suspension was sonicated for 1 h at 50 °C under a static N_2_ atmosphere. The solution was transferred to a flask containing pre-dried Cs_2_CO_3_ (1.303 g, 4.00 mmol) with activated 4 Å molecular sieves and stirred at 90 °C under a static N_2_ atmosphere for 90 min. Ethyl bromoacetate **16** (2.22 mL, 20 mmol) was added via syringe. The reaction mixture was stirred vigorously for 12 min 10 s at 80 °C under a static N_2_ atmosphere. The reaction mixture was then quenched by pouring into crushed ice (200 mL) and transferred to a separatory funnel. The aqueous layer was extracted with ethyl acetate (3 × 40 mL). The combined organic layers were washed with H_2_O (1 × 30 mL), brine (1 × 30 mL), dried (MgSO_4_) and concentrated in vacuo. The mixture was adsorbed on silica and loaded to a silica gel (100 g) column, and elution with (100% hexane → 10% EtOAc/hexane → 2% MeOH/DCM) resulted in three major fractions (F_1_, F_2_, and F_3_) including baseline decomposition. Fraction F_1_ contained an intense blue papery solid **19** and fraction F_2_ was subjected to silica gel (30 g) column chromatography, and the mixture was eluted with (100% hexane → EtOAc 10% to 20%) to afford a partially pure compound. Further purification by recrystallisation in 1:9 EtOAc:hexane gave compound **21** in pure form as a dark blue waxy solid.

### 3.3. Reactions of Indirubin with Ethyl Bromoacetate

#### 3.3.1. Alkylation Reaction of Indirubin **1a** with Ethyl Bromoacetate **16**

Indirubin **1a** (131.16 mg, 0.50 mmol) was dissolved in DMF (20 mL) and sonicated for 10 min under a nitrogen atmosphere for further dissolution. The resulting suspension was transferred via syringe to a flask containing pre-dried Cs_2_CO_3_ (390.98 mg, 1.20 mmol) and continued stirring at 90 °C. After 90 min, the reaction temperature was reduced to 70 °C, and ethyl bromoacetate **16** (0.27 mL, 2.50 mmol) was added via syringe and continued stirring vigorously under a nitrogen environment. After 1 h of reaction time, the mixture was poured into crushed ice (100 mL). When the ice melted, the resulting suspension was transferred to the separating funnel and the aqueous layer was partitioned with ethyl acetate (3 × 30 mL). The combined organic layers were washed with H_2_O (1 × 30 mL), with brine (1 × 20 mL), dried (MgSO_4_), and concentrated in vacuo. The residue was purified by silica gel column chromatography and eluted with 5% EtOAc/hexane to 100% EtOAc yielding *N*-alkylated indirubin **25** (82.97 mg, 48%) and *N*,*N*′-dialkylated indirubin **26** (44.50 mg, 20%) as a dark pink and dark purple fluffy solids.

#### 3.3.2. Cascade Reaction of Indirubin **1a** with Ethyl Bromoacetate **16**

Anhydrous DMF (20 mL) was added via syringe to a flask containing indirubin **1a** (131.13 mg, 0.50 mmol) under a nitrogen atmosphere. The resulting mixture was sonicated for 10 min at 50 °C under a static N_2_ atmosphere. The solution was transferred via cannula to a flask containing pre-dried Cs_2_CO_3_ (684.22 mg, 4.1 mmol), and the mixture was heated at 90 °C for 90 min. Ethyl bromoacetate **16** (0.27 mL, 2.5 mmol, 5 eq) was then added via syringe, the nitrogen inlet was removed, and the reaction was stirred vigorously for 1 h at 110 °C. The reaction mixture was quenched in ice water (150 mL), and the organic layer was extracted with ethyl acetate (2 × 40 mL), brine (1 × 20 mL), washed with H_2_O (1 × 30 mL), dried (MgSO_4_), and concentrated in vacuo. The residue was subjected to silica gel (70 g) column chromatography eluting with 10% EtOAc/hexane → 100% EtOAc, which afforded four major fractions (F_1_, F_2_, F_3_, and F_4_). Fraction F_1_ was identified as the disintegration product *N*-alkylated isatin **30** obtained as a yellow waxy solid. The fractions F_2_ and F_3_ were recrystallized (1:5 EtOAc/Hex) by slow evaporation to afford a pink solid **25** and a purple solid **26**. Fraction F_4_ was purified by multiple rounds of PTLC using (2% MeCN/CHCl_3_) developing solvent mixture and gave compounds **27**–**29** as orange-yellow, red-pink, and dark purple solids, respectively.

#### 3.3.3. Mechanistic Investigation of *N*,*N*′-Dialkylated Indirubin **26**

A 50 mL round-bottomed flask containing a stirring bar was charged with anhydrous caesium carbonate (65.16 mg, 0.2 mmol). The solution of *N*,*N*′-dialkylated indirubin **26** (43.41 mg, 0.1 mmol) in anhydrous DMF (15 mL) was added, and the mixture was vigorously stirred at 110 °C under a nitrogen environment. After 3.5 h, the resulting mixture was poured into crushed ice (50 mL), and the organic layer was extracted with ethyl acetate (3 × 20 mL). The combined organic layers were washed with H_2_O (1 × 40 mL), brine (1 × 30 mL), dried (MgSO_4_), and concentrated in vacuo. The crude mixture was purified by multiple rounds of preparative TLC using 1–2% MeCN/CHCl_3_ and afforded compounds **27** (13%), **28** (15%), and **29** (36%) as orange-yellow, red-pink, and dark-purple solids, respectively.


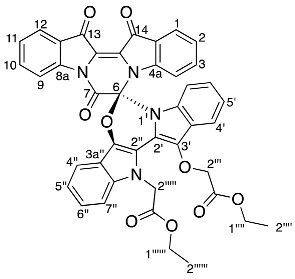

*Ethyl (R)-2-(13′-(2-ethoxy-2-oxoethoxy)-7,13,14-trioxo-13,14-dihydro-7H,12′H-spiro[pyrazino[1,2-a:4,3-a′]diindole-6,6′-[1,3]oxazino[3,4-a:5,6-b′]diindol]-12′-yl)acetate* **17**

Yield (18.7 mg, 4%), as an amorphous yellow solid. ^1^H NMR (500 MHz, CDCl_3_) δ 8.27 (1H, d, *J* = 8.2 Hz, H9), 7.99 (1H, d, *J* = 7.2 Hz, H12), 7.91 (1H, d, *J* = 7.1 Hz, H1), 7.61 (1H, td, *J* = 7.8, 1.2 Hz, H10), 7.59 (1H, d, *J* = 8.0 Hz, H4′), 7.49 (1H, d, *J* = 8.0 Hz, H4′′), 7.40 (1H, t, *J* = 7.3 Hz, H11), 7.31 (1H, td, *J* = 7.8, 1.3 Hz, H3), 7.26 (1H, m, H7′′), 7.25 (1H, m, H6′′), 7.14 (1H, t, *J* = 7.4 Hz, H2), 7.11 (1H, td, *J* = 7.6, 0.8 Hz, H5′), 7.08 (1H, td, *J* = 7.1, 1.6 Hz H5′′), 7.02 (1H, td, *J* = 7.1, 1.0 Hz, H6′), 6.96 (1H, d, *J* = 8.4 Hz, H7′), 6.87 (1H, d, *J* = 8.2 Hz, H4), 5.75 (1H, d, *J* = 18.0 Hz, H2′′′′a), 5.54 (1H, d, *J* = 18.0 Hz, H2′′′′b), 4.97 (2H, s, H2′′′), 4.31 (2H, qd, *J* = 7.2, 1.7 Hz, H1′′′′), 4.25 (2H, q, *J* = 7.2 Hz, H1′′′′′′), 1.31 (3H, t, *J =* 7.2 Hz, H2′′′′), 1.29 (3H, t, *J* = 7.2 Hz, H2′′′′′′). ^13^C NMR (126 MHz, CDCl_3_) 180.4 (C14), 178.6 (C13), 169.5 (C1′′′′′), 168.6 (C1′′′), 155.4 (C7), 148.0 (C4a), 144.4 (C8a), 136.9 (C3), 136.8 (C7a′′), 136.2 (C10), 133.5 (C7a′), 133.3 (C3′), 132.4 (C3′′), 126.8 (C11), 125.6 (C1), 125.5 (C12a), 124.8 (C6′), 124.7 (C12), 124.4 (C2), 124.2 (C7′′), 123.5 (C14a), 122.8 (C3a′), 122.2 (C5′), 120.5 (C5′′), 118.2 (C4′), 117.8 (C4′′), 117.8 (C9), 117.1 (C3a′′), 114.0 (C4), 113.9 (C2′′), 110.3 (C7′), 109.5 (C6′′), 92.6 (C6), 70.3 (C2′′′), 61.5 (C1′′′′′′), 61.4 (C1′′′′), 47.7 (C2′′′′′), 14.2 (C2′′′′′′), 14.1 (C2′′′′). FTIR (neat) ν*_max_* 2919 (s), 2850 (m), 1732 (s), 1595 (s), 1462 (s), 1372 (s), 1157 (s), 1095 (s), 902 (s), 738 (s), 685 (m), 425 (m) cm^−1^. LRMS-ESI (100%) 757 [M + Na]^+^, HRMS-ESI C_42_H_31_N_4_O_9_ calculated 735.2091, found 735.2086.


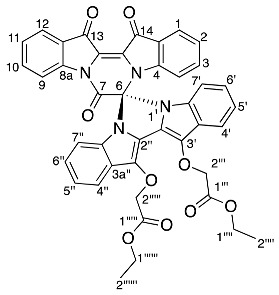

*Diethyl 2,2′-((7′,13′,14′-trioxo-13′,14′-dihydro-7′H-spiro[imidazo[1,5-a:3,4-a′]diindole-6,6′-pyrazino[1,2-a:4,3-a′]diindole]-12,13-diyl)bis(oxy))diacetate* **18**

Yield (30.2 mg, 8%), as an amorphous yellow solid, ^1^H NMR (500 MHz, CDCl_3_) 8.30 (1H, d, *J* = 8.2 Hz, H9), 8.06 (1H, d, *J* = 7.2 Hz, H12), 7.82 (1H, d, *J* = 7.0 Hz, H1), 7.72 (2H, d, *J* = 7.4 Hz, H4′),7.66 (1H, td, *J* = 7.8, 1.3 Hz, H10), 7.47 (1H, t, *J* = 7.1 Hz, H11), 7.24 (1H, td, *J* = 7.8, 1.3 Hz, H3), 7.07 (5H, m, H2, H5′ and H6′), 6.79 (2H, d, *J* = 7.8 Hz, H7′), 6.38 (1H, d, *J* = 8.3 Hz, H4), 5.25 (2H, d, *J* = 16.0 Hz, H2′′′a and H2′′′′′a), 5.15 (2H, d, *J* = 16.0 Hz, H2′′′b and H2′′′′′b), 4.31 (4H, q, *J* = 7.2 Hz, H1′′′′ and H1′′′′′′), 1.31 (6H, t, *J* = 7.2 Hz, H2′′′′ and H2′′′′′′). ^13^C NMR (126 MHz, CDCl_3_) 180.1 (C14), 178.5 (C13), 168.9 (C1′′′), 155.1 (C7), 147.4 (C4a), 143.9 (C8a), 137.2 (C3), 136.4 (C10), 132.0 (C3′), 130.2 (C7a′), 127.4 (C11), 126.2 (C3a′), 125.8 (C1), 125.5 (C12a), 125.0 (C6′), 124.8 (C12), 124.6 (C2), 124.4 (C13b), 123.0 (C14a), 121.6 (C5′), 120.2 (C4′), 117.8 (C9), 117.6 (C2′), 116.5 (C13a), 113.0 (C4), 108.4 (C7′), 82.6 (C6), 70.5 (C2′′′), 61.4 (C1′′′), 14.0 (C2′′′′). FTIR (neat) ν*_max_* 2919 (s), 2850 (m), 1703 (s), 1645 (s), 1462 (s), 1303 (s), 1192 (s), 1120 (s), 1014 (s), 937 (m), 738 (s), 685 (m), 538 (m) cm^−1^. LRMS-ESI (100%) 757 [M + Na]^+^. HRMS-ESI for C_42_H_30_N_4_O_9_Na calculated 757.1910, found 757.1927.


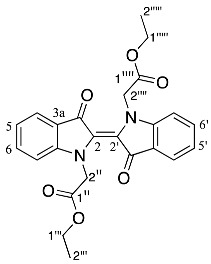

*Diethyl (E)-2,2′-(3,3′-dioxo-[2,2′-biindolinylidene]-1,1′-diyl) diacetate* **19**

Yield (98.2 mg, 22%), as a dark blue waxy solid, R*_f_* (20% EtOAc/hexane) 0.53; mp. 244–245 °C, ^1^H NMR (400 MHz, CDCl_3_) δ 7.74 (1H, d, *J* = 7.7 Hz, H4 and H4′), 7.53 (1H, ddd, *J* = 8.2, 7.3, 1.3 Hz, H6 and H6′), 7.07 (1H, td, *J* = 7.4, 0.8 Hz, H5 and H5′), 7.02 (1H, d, *J* = 8.2, Hz, H7, and H7′), 4.82 (4H, s, H2′′ and H2′′′′), 4.21 (4H, q, *J* = 7.1 Hz, H1′′′ and H1′′′′′), 1.25 (6H, t, *J* = 7.1 Hz, H2′′′ and H2′′′′′). ^13^C NMR (101 MHz, CDCl_3_) δ 186.3 (C3 and C3′), 168.9 (C1′′ and C1′′′′), 153.7 (C7a and C7a′), 135.6 (C6 and C6′), 127.0 (C2 and C2′), 124.4 (C4 and C4′), 122.0 (C3a and C3a′), 121.9 (C5 and C5′), 111.0 (C7 and C7′), 61.4 (C1′′′ and C1′′′′′), 51.0 (C2′′ and C2′′′′), 14.1 (C2′′′ and C2′′′′′). FTIR (neat) ν*_max_* 2982 (s), 2935 (s), 1739 (m), 1650 (s), 1607 (s), 1470 (s), 1373 (s), 1202 (s), 1083 (s), 753 (s) cm^−1^. UV-vis (CH_2_Cl_2_) *λ*_max_/nm (ε M^−1^cm^−1^) 624.00 (2709096). LRMS-ESI (100%) 435 [M + H]^+^, HRMS-ESI for C_24_H_22_N_2_O_6_Na calculated 457.1363, found 457.1376.


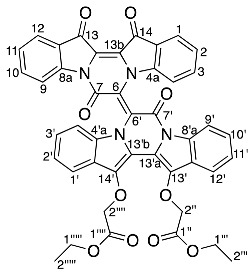

*Diethyl (E)-2,2′-((7,7′,13′,14′-tetraoxo-13′,14′-dihydro-7H,7′H-[6,6′-bipyrazino[1,2-a:4,3-a′]diindolylidene]-13,14-diyl)bis(oxy)) diacetate* **20**

Yield (18.9 mg, 4%), as a purple powder. R*_f_* (30% EtOAc/P.S) 0.33; mp 214–220 °C. ^1^H NMR (500 MHz, CDCl_3_) 8.38 (1H, d, *J* = 8.4 Hz, H9), 8.35 (1H, d, *J* = 7.4 Hz, H9′), 8.04–7.96 (1H, dd, *J* = 0.9, 7.7 Hz, H1), 7.92 (1H, dd, *J* = 0.7, 7.6 Hz, H12), 7.90–7.88 (1H, m, H12′), 7.78–7.71 (1H, m, H1′), 7.59–7.54 (2H, m, H3 and H10), 7.53–7.45 (1H, m, H4′), 7.44–7.38 (1H, m, H4), 7.37–7.27 (6H, m, H2, H11, H2′, H3′, H10′ and H11′), 5.04 (1H, d, *J* = 16.1 Hz, H2′′b) 4.93 (1H, d, *J* = 15.8 Hz, H2′′′′b), 4.82 (1H, d, *J* = 16.1 Hz, H2′′a), 4.74 (1H, d, *J* = 15.8 Hz, H2′′′′a), 4.36–4.12 (4H, m, H1′′′ and H1′′′′′), 1.32 (3H, t, *J* = 7.2 Hz, H2′′′), 1.29 (3H, t, *J* = 7.2 Hz, H1′′′′′). ^13^C NMR (126 MHz, CDCl_3_) 178.6 (C14), 178.4 (C13), 169.1 (C1′′′′), 169.1 (C1′′), 154.8 (C7), 153.6 (C7′), 150.8 (C4a), 145.6 (C8a), 139.4 (C14′), 139.2 (C13′), 135.9 (C10), 135.8 (C3, C8a′), 132.9 (C4a′), 126.6 (C11′), 126.5 (C10′), 125.6 (C12a′), 125.5 (C3′), 125.4 (C1, C11), 125.1 (C2′), 124.9 (C12), 124.4 (C12a), 124.2 (C14a′), 123.4 (C14a), 123.2 (C2), 122.8 (C6), 120.8 (C13b), 119.6 (C12′), 119.0 (C13a), 118.8 (C1′), 118.2 (C9), 116.8 (C9′), 116.6 (C6′), 114.0 (C13b′), 113.4 (C13a′), 113.1 (C4), 112.5 (C4′), 71.4 (C2′′), 71.3 (C2′′′′), 61.3 (C1′′′), 61.2 (C1′′′′′′), 14.2 (C2′′′), 14.1 (C2′′′′′). FTIR (neat) ν*_max_* 1766 (m), 1716 (m), 1462 (m), 1299 (s), 1191 (s), 1063 (s), 755 (s) cm^−1^. LRMS-ESI (100%) 798 [M + Na]^+^, HRMS-ESI for calculated C_44_H_30_N_4_O_10_Na 797.1860, found 797.1859.


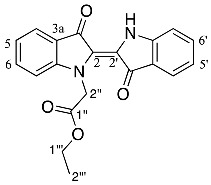

*Ethyl (E)-2-(3,3′-dioxo-[2,2′-biindolinylidene]-1-yl)acetate* **21**

Yield (95.8 mg, 27%), as an intense blue papery solid, R*_f_* (20% EtOAc/hexane) 0.75; mp. 217–220 °C, ^1^H NMR (400 MHz, CDCl_3_) δ 10.58 (1H, s, NH), 7.77 (1H, d, *J* = 7.7 Hz, H4), 7.65 (1H, d, *J* = 7.6 Hz, H4′), 7.54 (1H, ddd, *J* = 8.4, 7.2, 1.3 Hz, H6), 7.46 (1H, ddd, *J* = 8.1, 7.2, 1.3 Hz, H6′), 7.06 (1H, ddd, *J* = 7.8, 7.2, 0.7 Hz, H5), 6.99 (1H, d, *J* = 8.0 Hz, H7′), 6.97 (1H, d, *J* = 8.1 Hz, H7), 6.93 (1H, ddd, *J* = 7.9, 7.3, 0.8 Hz, H5′), 5.34 (2H, s, H2′′), 4.21 (2H, q, *J* = 7.1 Hz, H1′′′), 1.26 (3H, t, *J* = 7.1 Hz, H2′′′). ^13^C NMR (101 MHz, CDCl_3_) δ 189.3 (C3), 187.6 (C3′), 169.0 (C1′′), 152.7 (C7a), 151.8 (C7a′), 136.4 (C6′), 135.9 (C6), 125.7 (C2′), 124.9 (C4′), 124.3 (C4), 123.4 (C2), 121.3 (C5), 121.1 (C3a), 120.7 (C5′), 120.1 (Ca′), 111.9 (C7′), 110.1 (C7), 61.5 (C1′′′), 49.0 (C2′′), 14.2 (C2′′′). FTIR (neat) ν*_max_* 3273 (s), 2926 (s), 2855 (s), 1739 (s), 1692 (s), 1640 (s), 1464 (s), 1430(s), 1209 (s), 1046 (s), 747 (s) cm^−1^. UV-vis (CH_2_Cl_2_) *λ*_max_/nm (ε M^−1^cm^−1^) 617.30 (164854), LRMS-ESI (100%) 349 [M + H]^+^, HRMS-ESI for C_20_H_16_N_2_O_4_Na calculated 371.1003, found 371.1008.


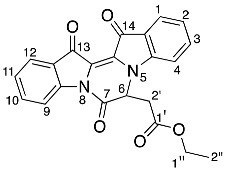

*Ethyl 2-(7,13,14-trioxo-6,7,13,14-tetrahydropyrazino[1,2-a:4,3-a′]diindol-6-yl) acetate* **22**

Yield (8.2 mg, 2%), as a dark pink powder, R*_f_* (40% EtOAc/hexane) 0.61; mp. 210–216 °C, ^1^H NMR (400 MHz, CDCl_3_) δ 8.49 (1H, d, *J* = 8.2 Hz, H4), 7.94 (1H, d, *J* = 7.9 Hz, H1), 7.85 (1H, d, *J* = 8.1 Hz, H12), 7.69 (1H, ddd, *J* = 8.4, 7.3, 1.4 Hz, H3), 7.60 (1H, ddd, *J* = 7.7, 1.3 Hz, H10), 7.38 (1H, td, *J* = 7.6, 0.9 Hz, H2), 7.15 (1H, t, *J* = 7.5 Hz, H11), 7.04 (1H, d, *J* = 8.0 Hz, H9), 5.17 (1H, dd, *J* = 5.2, 4.0 Hz, H6), 4.01 (2H, q, *J* = 7.2, Hz, H1′′), 3.31–3.12 (2H, m, H2′), 1.06 (3H, t, *J* = 7.2 Hz, H2′′). ^13^C NMR (101 MHz, CDCl_3_) δ 180.3 (C13), 178.4 (C14), 168.8 (C1′), 160.6 (C7), 149.1 (C8a), 144.3 (C4a), 137.0 (C13a), 136.3 (C10), 135.6 (C3), 126.2 (C12), 128.0 (C13b), 126.0 (C2), 125.4 (C14a), 124.3 (C1), 122.9 (C11), 122.6 (C12a), 117.2 (C4), 110.0 (C9), 61.7 (C1′′), 54.1 (C6), 35.7 (C2′) 13.9 (C2′′). FTIR (neat) ν*_max_* 2927 (s), 2854 (s), 1728 (s), 1700 (s), 1640 (s), 1604 (s), 1467 (s), 1300 (s), 1208 (s), 1030 (s), 754 (s) cm^−1^. UV-vis (CH_2_Cl_2_) *λ*_max_/nm (ε M^−1^cm^−1^) 526.00 (1134143). LRMS-ESI (100%) 389 [M + H]^+^, HRMS-ESI for C_22_H_16_N_2_O_5_Na calculated 411.0957, found 411.0959.


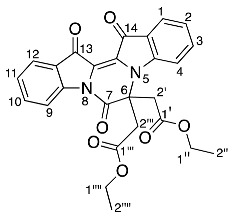

*Diethyl 2,2′-(7,13,14-trioxo-6,7,13,14-tetrahydropyrazino[1,2-a:4,3-a′]diindole-6,6-diyl) diacetate* **23**

Yield (15.4 mg, 3%), as a red waxy solid, R*_f_* (40% EtOAc/hexane) 0.57; ^1^H NMR (400 MHz, CDCl_3_) δ 8.51 (1H, d, *J* = 8.2 Hz, H4), 7.96 (1H, d, *J* = 7.6 Hz, H1), 7.89 (1H, dd, *J* = 7.4, 1.7 Hz, H12), 7.70 (1H, ddd, *J* = 8.3, 7.4, 1.4 Hz, H3), 7.58 (1H, ddd, *J* = 8.4, 7.4, 1.5 Hz, H10), 7.38 (1H, td, *J* = 7.5, 0.9 Hz, H2), 7.17 (1H, d, *J* = 8.2 Hz, H9), 7.15 (1H, t, *J* = 7.5 Hz, H11), 3.92 (4H, q, *J* = 14.21 Hz, H1′′ and H1′′′′), 3.63–3.47 (4H, m, H2′ and H2′′′), 0.93 (6H, t, *J* = 14.26 Hz, H2′′, and H2′′′′). ^13^C NMR (101 MHz, CDCl_3_) δ 180.3 (C13), 178.7 (C14), 167.9 (C1′), 163.8 (C7), 148.3 (C8a), 144.2 (C4a), 135.9 (C10), 135.5 (C3), 131.3 (C13a), 126.3 (C12), 125.8 (C2), 125.4 (C14a), 124.3 (C1), 123.2 (C12a), 122.5 (C11), 117.8 (C13b), 117.1 (C4), 111.6 (C9), 63.5 (C6), 61.5 (C1′′ and C1′′′), 40.8 (C2′ and C2′′′), 13.8 (C2′′ and C2′′′′). FTIR (neat) ν*_max_* 2954 (s), 2925 (s), 2870 (s), 1735 (m), 1638 (s), 1604 (s), 1466 (s), 1197 (m), 1098 (s), 758 (s) cm^−1^. UV-vis (CH_2_Cl_2_) *λ*_max_/nm (ε M^−1^cm^−1^) 523.00 (4959190). LRMS-ESI (100%) 475 [M + H]^+^, HRMS-ESI for C_26_H_22_N_2_O_7_H calculated 475.1504, found 475.1505.


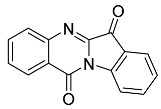

*Tryptanthrin* **24**

Yield 5.4 mg, 2%), yellow waxy solid. ^1^H NMR (400 MHz, CDCl_3_) δ 8.51 (d, *J* = 8.12 Hz, 1H), 8.39 (d, *J* = 1.22 Hz, 1H), 7.99 (d, *J* = 7.64 Hz, 1H), 7.86–7.72 (m, 3H), 7.60 (t, *J* = 7.1 Hz, 1H), 7.36 (t, *J* = 7.0, 0.56 Hz, 1H), LRMS-ESI 249 [M + H]^+^. This was consistent with the data in [13].


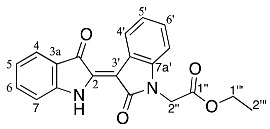

*Ethyl (Z)-2-(2′,3-dioxo-[2,3′-biindolinylidene]-1′-yl)acetate* **25**

Yield (1.8 mg, <1%), as a dark pink fluffy solid, R*_f_* (20% EtOAc/hexane) 0.68; mp. 272–273 °C, ^1^H NMR (400 MHz, CDCl_3_) δ 10.46 (1H, s, NH), 8.92 (1H, d, *J* = 7.3 Hz, H4′), 7.74 (1H, d, *J* = 7.7 Hz, H4), 7.51 (1H, t, *J* = 8.0 Hz, H6), 7.30 (1H, td, *J* = 7.7, 1.2 Hz, H6′), 7.16 (1H, td, *J* = 7.7, 1.1 Hz, H5′), 7.02 (1H, td, *J* = 7.5 Hz, H5), 6.98 (1H, d, *J* = 7.9 Hz, H7), 6.78 (1H, d, *J* = 7.7 Hz, H7′), 4.58 (2H, s, H2′′), 4.24 (2H, q, *J* = 7.1 Hz, H1′′′), 1.27 (3H, t, *J* = 7.1 Hz, H2′′′). ^13^C NMR (101 MHz, CDCl_3_) δ 188.2 (C3), 170.7 (C2′), 167.6 (C1′′), 151.6 (C7a), 140.7 (C7a′), 139.7 (C3a′), 137.0 (C6), 129.2 (C6′), 125.7 (C4′), 125.3 (C4), 123.1 (C5′), 121.8 (C5), 121.2 (C2), 120.1 (C3a), 111.9 (C7), 107.8 (C7′), 106.0 (C3′), 61.7 (C1′′′), 41.3 (C2′′), 14.2 (C2′′′). FTIR (neat) ν*_max_* 3307 (s), 2920 (s), 1739 (s), 1654 (s), 1629 (s), 1469 (s), 1220 (s), 747 (s) cm^−1^. UV-vis (CH_2_Cl_2_) *λ*_max_/nm (ε M^−1^cm^−1^) 534.00 (50950), LRMS-ESI (100%) 349 [M + H]^+^, HRMS-ESI for C_20_H_16_N_2_O_4_Na calculated 371.1011, found 371.1008.


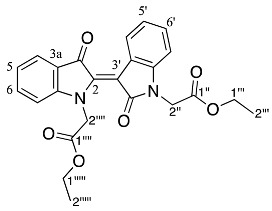

*Diethyl (Z)-2,2′-(2′,3-dioxo-[2,3′-biindolinylidene]-1,1′-diyl) diacetate* **26**

Yield (54.2 mg, 25%), as a dark purple fluffy solid, R*_f_* (20% EtOAc/hexane) 0.48; mp. 151–152 °C, ^1^H NMR (500 MHz, CDCl_3_) δ 8.72 (1H, ddd, *J* = 8.0, 1.2, 0.6 Hz, H4′), 7.75 (1H, ddd, *J* = 7.6, 1.4, 0.6 Hz, H4), 7.56 (1H, ddd, *J* = 8.2, 7.4, 1.3 Hz, H6), 7.27 (1H, td, *J* = 7.7, 1.2 Hz, H6′), 7.12 (1H, td, *J* = 7.5, 0.8 Hz, H5), 7.08 (1H, td, *J* = 7.7, 1.1 Hz, H5′), 6.99 (1H, d, *J* = 8.1 Hz, H7), 6.70 (1H, dt, *J* = 7.8, 0.9 Hz, 7′), 4.86 (2H, s, H2′′′′), 4.53 (2H, s, H2′′), 4.26 (2H, q, *J* = 7.1 Hz, H1′′′), 4.22 (2H, q, *J* = 7.1 Hz, H1′′′′′), 1.27 (3H, t, *J* = 7.1 Hz, H2′′′′′). 1.25 (3H, t, *J* = 7.1 Hz, H2′′′). ^13^C NMR (126 MHz, CDCl_3_) δ 187.8 (C3), 168.9 (C1′′′′), 167.8 (C1′′), 166.9 (C2′), 154.3 (C7a), 143.2 (C2), 141.5 (C7a′), 136.7 (C6), 129.9 (C6′), 126.1 (C4′), 124.9 (C4), 122.9 (C5), 122.3 (C5′), 121.8 (C3a′), 121.4 (C3a), 111.4 (C7), 110.4 (C3′), 107.7 (C7′), 61.7 (C1′′′), 61.5 (C1′′′′′), 51.8 (C2′′′′), 41.5 (C2′′), 14.1 (C2′′′ and C2′′′′). FTIR (neat) ν*_max_* 2957 (s), 2850 (s), 1741 (s), 1680 (s), 1610 (s), 1470 (s), 1337 (s), 1205 (s), 753 (s) cm^−1^. UV-vis (CH_2_Cl_2_) *λ*_max/_nm (ε M^−1^cm^−1^) 555.50 (67336), LRMS-ESI (100%) 435 [M + H]^+^, HRMS-ESI for C_24_H_22_N_2_O_6_Na calculated 457.1377, found 457.1376.


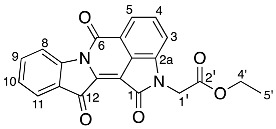

*Ethyl 2-(1,6,12-trioxo-6,12-dihydroindolo[1,2-b]pyrrolo[4,3,2-de]isoquinolin-2(1H)-yl) acetate* **27**

Yield (8.2 mg, 4%), as an orange yellow amorphous solid, R*_f_* (3% MeCN/CHCl_3_) 0.86; ^1^H NMR (400 MHz, CDCl_3_) δ 8.81 (1H, d, *J* = 8.2 Hz, H8), 7.92 (1H, dd, *J* = 7.5, 2.1 Hz, H11), 7.87 (1H, d, *J* = 8.1 Hz, H5), 7.75 (1H, ddd, *J* = 8.3, 7.5, 1.4 Hz, H9), 7.57 (1H, t, *J* = 7.8 Hz, H4), 7.40 (1H, td, *J* = 7.5, 0.9 Hz, H10), 6.99 (1H, d, *J* = 7.6 Hz, H3), 4.62 (2H, s, H1′), 4.25 (2H, q, *J* = 7.1 Hz, H4′), 1.29 (3H, t, *J* = 7.1 Hz, H5′). ^13^C NMR (126 MHz, CDCl_3_) δ 180.8 (C12), 167.4 (C2′), 162.2 (C1), 159.0 (C6), 148.6 (C7a), 141.4 (C2a), 137.3 (C9), 134.9 (C12a), 132.4 (C4), 126.9 (C10), 126.5 (C2b), 125.6 (C5a), 125.3 (C11), 123.4 (C11a), 120.0 (C5), 118.5 (C8), 111.3 (C3), 109.9 (C12b), 62.0 (C4′), 41.8 (C1′), 14.2 (C5). FTIR (neat) ν*_max_* 2953 (s), 2917 (s), 2850 (s), 1731 (m), 1710 (m), 1624 (m), 1461 (s), 1337 (s), 1205 (s), 760 (s) cm^−1^. UV-vis (CH_2_Cl_2_) *λ*_max_/nm (ε M^−1^cm^−1^) 457.50 (2104), LRMS-ESI 375 [M + H]^+^, HRMS-ESI for C_22_H_10_N_6_O_5_Na calculated 397.0814, found 397.0815.


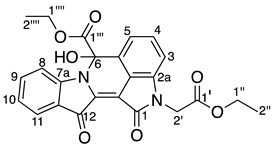

*Ethyl 2-(2-ethoxy-2-oxoethyl)-6-hydroxy-1,12-dioxo-1,2,6,12-tetrahydroindolo[1,2-b]pyrrolo[4,3,2-de]isoquinoline-6-carboxylate* **28**

Yield (5.4 mg, 2%), as a red, pink, waxy solid, R*_f_* (3% MeCN/CHCl_3_) 0.28; mp. 280–281 °C, ^1^H NMR (500 MHz, CDCl_3_) δ 7.67 (1H, dd, *J* = 7.6, 1.3 Hz, H11), 7.52 (1H, ddd, *J* = 8.2, 7.4, 1.4 Hz, H9), 7.33 (1H, t, *J* = 7.8 Hz, H4), 7.22 (1H, d, *J* = 8.2, Hz, H8), 7.11 (1H, dd, *J* = 8.0, 0.5 Hz, H5), 7.08 (1H, td, *J* = 7.5, 0.8 Hz, H10), 6.69 (1H, d, *J* = 7.8 Hz, H3), 5.39 (1H, s, OH), 4.43 (1H, d, *J* = 17.7 Hz, H2′a), 4.31 (1H, d, *J* = 17.1 Hz, H2′b), 4.28–4.15 (4H, m, H1′′′′ and H1′′), 1.26 (3H, t, *J* = 7.1 Hz, H2′′′′), 1.08 (3H, t, *J* = 7.1 Hz, H2′′). ^13^C NMR (126 MHz, CDCl_3_) δ 181.8 (C12), 169.2 (C1′′′), 167.8 (C1′), 162.9 (C1), 150.3 (C7a), 139.6 (C2a), 136.4 (C9), 134.7 (C12a), 131.3 (C4), 126.7 (C5a), 125.4 (C11), 123.1 (C10), 121.8 (C11a), 119.2 (C2b), 117.7 (C5), 112.8 (C8), 108.5 (C3), 104.1 (C12b), 84.5 (C6), 63.9 (C1′′), 61.8 (C1′′′′), 41.5 (C2′), 14.1 (C2′′′′), 13.8 (C2′′). FTIR (neat) ν*_max_* 3376 (s), 2980 (s), 1744 (s), 1712 (s), 1592 (s), 1469 (s), 1303 (s), 1200 (s), 756 (s) cm^−1^. UV-vis (CH_2_Cl_2_) *λ*_max_/nm (ε M^−1^cm^−1^) 512.50 (3921), LRMS-ESI (100%) 449 [M + H]+, HRMS-ESI for C_25_H_16_N_6_O_3_Na for calculated 471.1182, found 471.1180.


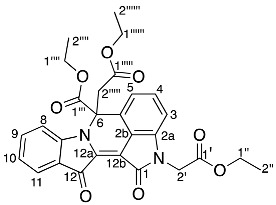

*Diethyl 2,2′-(6-(ethoxycarbonyl)-1,12-dioxo-6,12-dihydroindolo[1,2-b]pyrrolo[4,3,2-de]isoquinoline-2,6(1H)-diyl) diacetate* **29**

Yield (7.8 mg, 3%), as a dark purple waxy solid, R*_f_* (3% MeCN/CHCl_3_) 0.75; ^1^H NMR (500 MHz, CDCl_3_) δ 7.86 (1H, dd, *J* = 7.6, 1.5 Hz, H11), 7.52 (1H, ddd, *J* = 8.2, 7.4, 1.4 Hz, H9), 7.24 (1H, t, *J* = 7.9 Hz, H4), 7.12 (1H, td, *J* = 7.5, 0.7 Hz, H10), 6.86 (1H, d, *J* = 7.8 Hz, H5), 6.84 (1H, d, *J* = 8.2 Hz, H8), 6.63 (1H, d, *J* = 7.6 Hz, H3), 4.57 (1H, d, *J* = 17.7 Hz, H2′a), 4.48 (1H, d, *J* = 17.7 Hz, H2′b), 4.27 (2H, q, *J* = 7.1 Hz, H1′′′′), 4.22 (2H, q, *J* = 7.1 Hz, H1′′), 3.81 (2H, q, *J* = 7.1 Hz, H1′′′′′′), 3.63 (1H, d, *J* = 15.6 Hz, H2′′′′′a), 3.46 (1H, d, *J* = 15.5 Hz, H2′′′′′b), 1.28 (3H, t, *J* = 7.1 Hz, H2′′), 1.10 (3H, t, *J* = 7.1 Hz, H2′′′′), 0.86 (3H, t, *J* = 7.1 Hz, H2′′′′′′). ^13^C NMR (126 MHz, CDCl_3_) δ 181.5 (C12), 169.0 (C1′′′), 168.0 (C1′′′′′), 167.8 (C1′), 162.8 (C1), 150.3 (C7a), 140.0 (C2a), 136.3 (C12a), 136.0 (C9), 131.3 (C4), 126.0 (C5a), 125.9 (C11), 122.4 (C10), 121.9 (C11a), 119.2 (C2b), 116.5 (C5), 110.8 (C8), 108.0 (C3), 105.0 (C12b), 66.2 (C6), 63.4 (C1′′′′), 61.7 (C1′′), 61.0 (C1′′′′′′), 41.6 (C2′), 41.5 (C2′′′′′), 14.1 (C2′′), 13.8 (C2′′′′), 13.6 (C2′′′′′′). FTIR (neat) ν*_max_* 2956 (s), 2920 (s), 2851 (s), 1735 (s), 1720 (s), 1629 (s), 1604 (s), 1469 (s), 1302 (s), 1198 (s), 757 (s) cm^−1^. UV-vis (CH_2_Cl_2_) *λ*_max_/nm (ε M^−1^cm^−1^) 558.00 (3907), LRMS-ESI 519 [M + H]^+^, HRMS-ESI for C_28_H_26_N_2_O_8_H calculated 519.1781, found 519.1767.


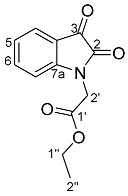

*Ethyl 2-(2,3-dioxo-1H-indol-1-yl)acetate* **30**

(Yield 3.5 mg, 3%), yellow waxy solid. ^1^H NMR (400 MHz, CDCl_3_) δ 8.63 (d, *J* = 8.4 Hz, 1H), 7.91 (d, *J* = 7.9 Hz, 1H), 7.63–7.55 (m, 1H), 7.40 (t, *J* = 7.5 Hz, 1H), 5.36 (s, 2H), 4.24 (q, *J* = 7.1 Hz, 2H), 1.27 (t, *J* = 7.2 Hz, 3H). LRMS-ESI 234 [M + H]^+^. This data were consistent with that reported in reference [28].

## 4. Conclusions

This study explored the reaction of indigo **1** with ethyl bromoacetate **16** in the presence of caesium carbonate as a base, revealing the new polyheterocyclic compounds **17**, **18** and **20**, albeit in low yield. Further optimisation of the reaction allowed enhanced control over the reaction to furnish key compounds **19**, and **21**–**23**, highlighting the influence of the dual electrophilic sites in ethyl bromoacetate **16** in these cascade processes. The acidic methylene hydrogen involvement led to numerous side reactions, resulting in high reactivity with less control of the reaction. Despite this, the optimised reactions yielded a diverse range of new polyheterocyclic compounds that could be challenging to prepare from a step-by-step synthesis. The cascade reaction of ethyl bromoacetate **16** with the more stable (and less reactive) indirubin **1a** under basic conditions yielded representatives of a new heterocyclic class, in particular dihydroindolopyrroloisoquinoline-2-accetate **27**, the tetrahydroindolopyrroloisoquinoline-6-carboxylate **28**, and the dihydroindolopyrroloisoquinoline-2,6-diacetate **29**, as well as *N*- and *N*,*N*′-alkylation products **25** and **26** respectively. Detailed mapping of the proposed cascade mechanistic pathways to these compounds is presented which should be useful in informing any future synthetic studies.

## Data Availability

The original contributions presented in the study are included in the article/Supplementary Material, further inquiries can be directed to the corresponding author.

## Supplementary Materials

The following supporting information can be downloaded at: https://www.mdpi.com/article/10.3390/molecules29174242/s1, Reports all NMR data for synthesised compounds, the X-ray crystallographic data for compounds **17**, **18** and **20**, and the proposed in situ synthesis of compounds **33** and **59**.
