# Supplementary material for: Mapping of Some Further Alkylation-Initiated Pathways to Polyheterocyclic Compounds from Indigo and Indirubin
Source: Molecules. 2024 Sep 6;29(17):4242. doi: 10.3390/molecules29174242 (PMC11396853; doi:10.3390/molecules29174242)
Supplement: Supplementary file 1 [file molecules-29-04242-s001.zip › molecules-3191280-supplementary.pdf]

Supplementary Material

Mapping of Some Further Alkylation-Initiated Pathways to Polyheterocyclic Compounds From Indigo and Indirubin

Sarfaraz Ali[1], Patrick M. McCosker[1], Anthony C. Willis[2], Stephen G. Pyne[1], Christopher Richardson [1], John B. Bremner[1], Paul A. Keller[1]*

1. *School of Chemistry and Molecular Biosciences, Molecular Horizons, University of Wollongong, Wollongong, NSW, 2522, Australia. E-mail: keller@uow.edu.au*
2. *Research School of Chemistry, Australian National University, Canberra, ACT 2601, Australia*

Table of Contents **Pages**

Figures S1 - S6 ^{1}H, DEPT, COSY, HMBC, HSQC, NOESY NMR spectra of compound **17**…S2-S4

Figures S7 - S12 ^{1}H, DEPT, COSY, HMBC, HSQC NOESY NMR spectra of compound **18** ...S5-S7

Figures S13 - S17 ^{1}H, ^{13}C, COSY, HMBC, HSQC NMR spectra of compound **19** S8-S10

Figures S18 – S22 ^{1}H, DEPT, COSY, HMBC, HSQC NMR spectra of compound **20** S10-S12

Figures S23 - S27 ^{1}H, ^{13}C, COSY, HMBC, HSQC NMR spectra of compound **21** ……….. S13-S15

Figures S28 - S32 ^{1}H, ^{13}C, COSY, HMBC, HSQC NMR spectra of compound **22**……….. S15-S17

Figures S33- S37 ^{1}H, ^{13}C, COSY, HMBC, HSQC NMR spectra of compound **23** ………...S18-S20

Figures S38 - S42 ^{1}H, ^{13}C, COSY, HMBC, HSQC NMR spectra of compound **25** S20-S22

Figures S43- S47 ^{1}H, ^{13}C, COSY, HMBC, HSQC NMR spectra of compound **26** ………...S23-S25

Figures S48 - S52 ^{1}H, ^{13}C, COSY, HMBC, HSQC NMR spectra of compound **27** ………...S25-S27

Figures S53 - S57 ^{1}H, ^{13}C, COSY, HMBC, HSQC NMR spectra of compound **28** ………...S28-S30

Figures S58 - S62 ^{1}H, ^{13}C, COSY, HMBC, HSQC NMR spectra of compound **29** ………...S30-S32

X-ray Crystallography Data for Compound **18**, Compound **17**, and Compound **20** ……………………………………………………….………………………..…S33-S35

Scheme S1. Proposed synthesis of intermediates **59**, and **33**, involved in the cascade reaction of indirubin **1a** and indigo **1.** …………………………………………….………………………… S35

NMR spectra of compound 17.

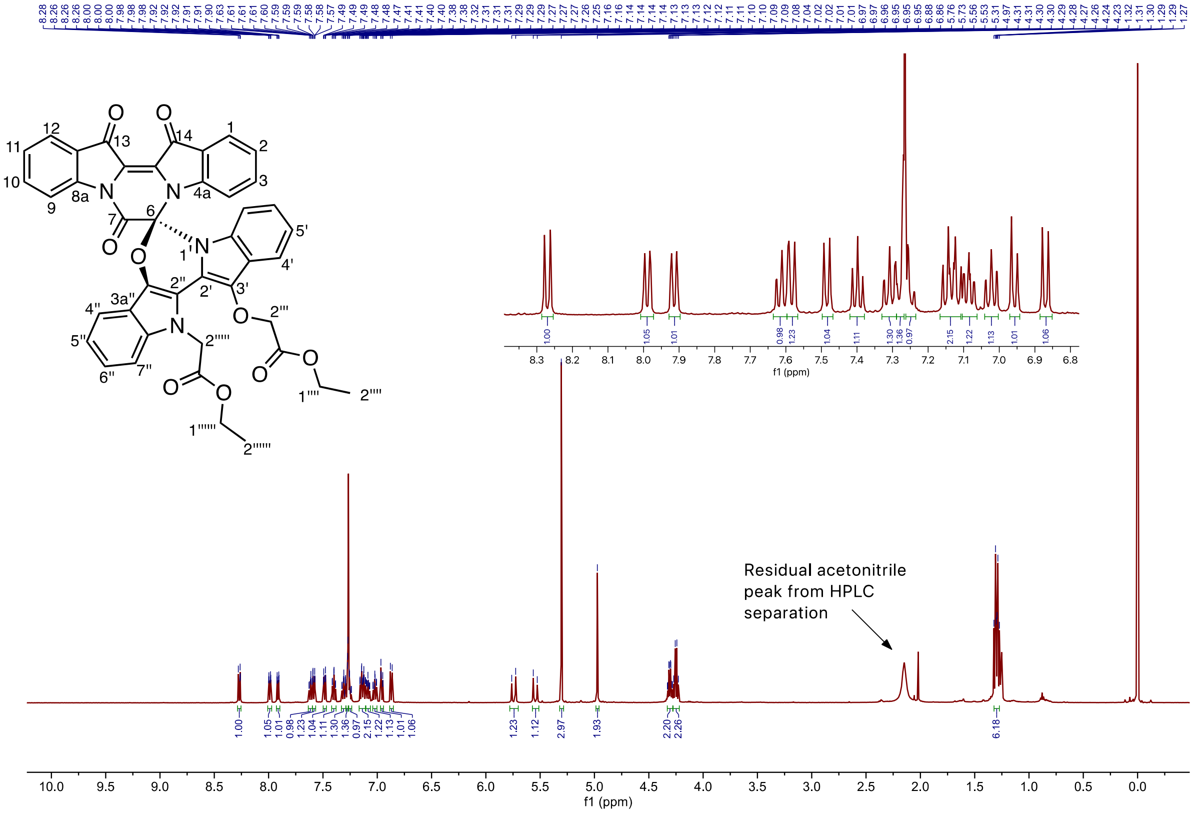

Figure S1: ^{1}H NMR spectrum of the compound **17** ($CDCl_3$, 500 MHz).

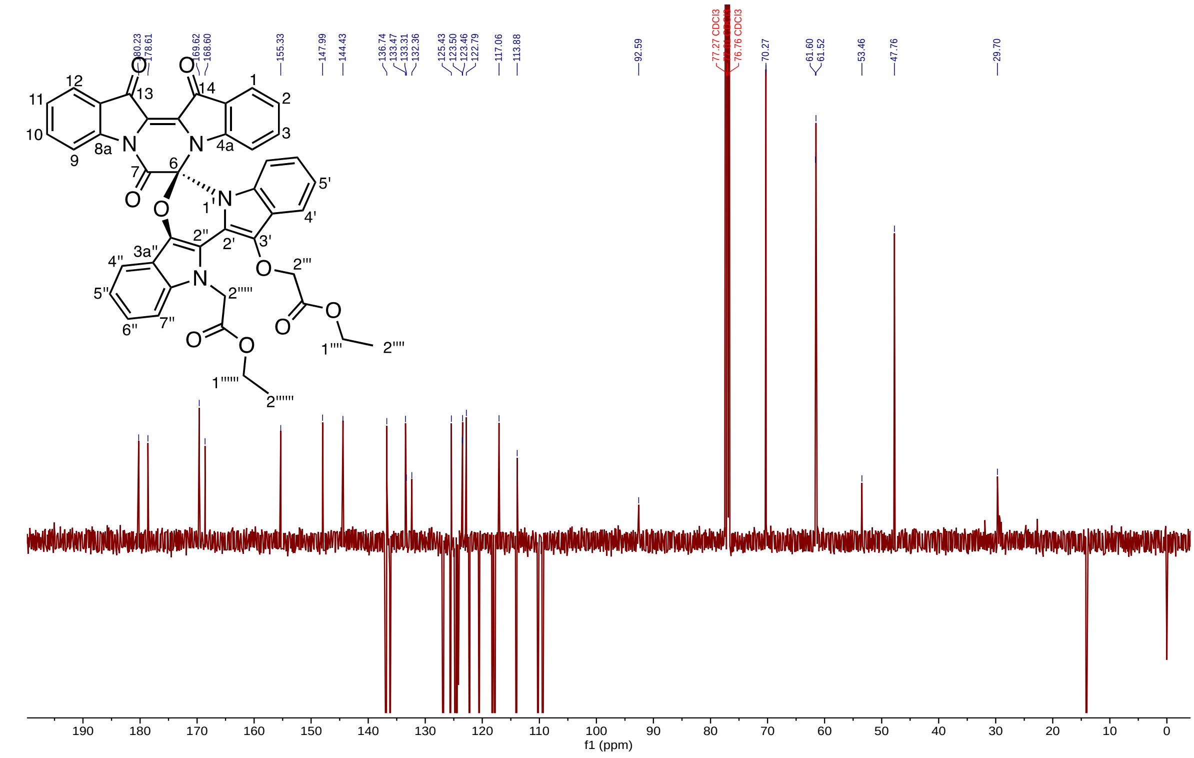

Figure S2: DEPT NMR spectrum of the compound **17** ($CDCl_3$, 125 MHz).

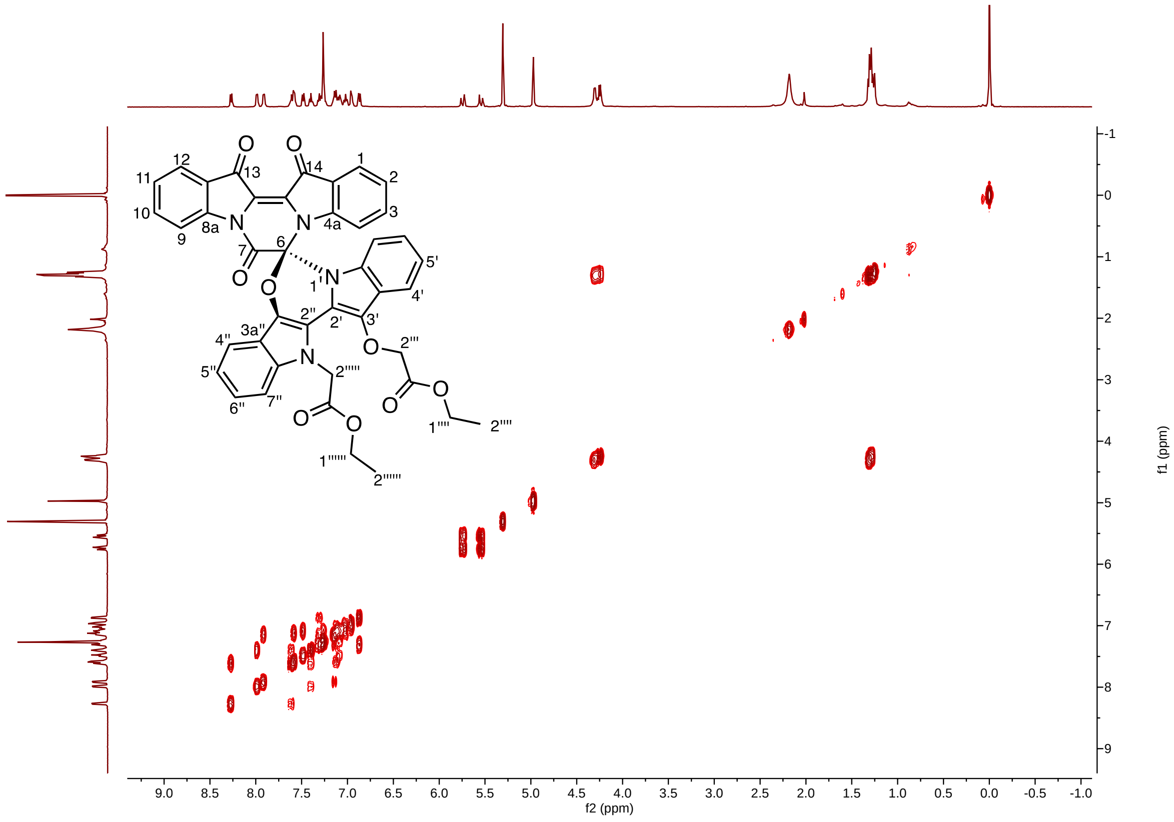

Figure S3: COSY spectrum of the compound **17** ($CDCl_3$, 500 MHz).

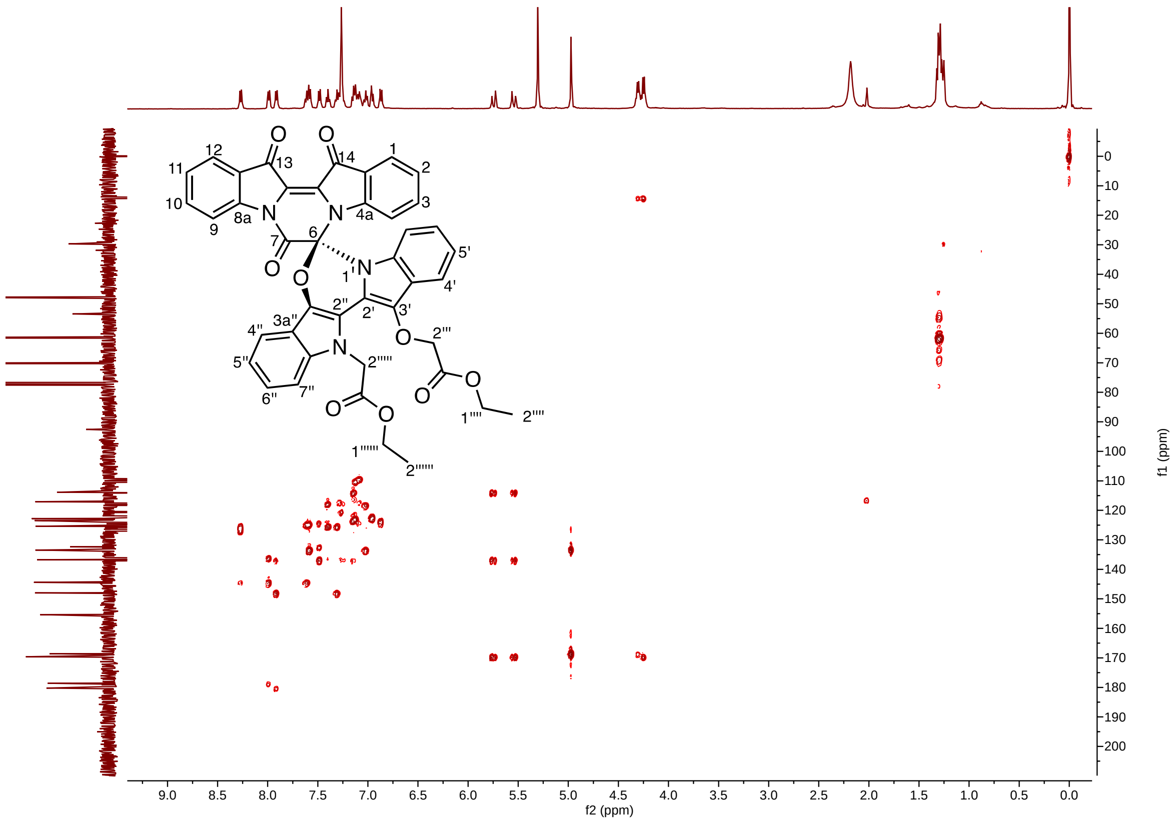

Figure S4: HMBC spectrum of the compound **17**.

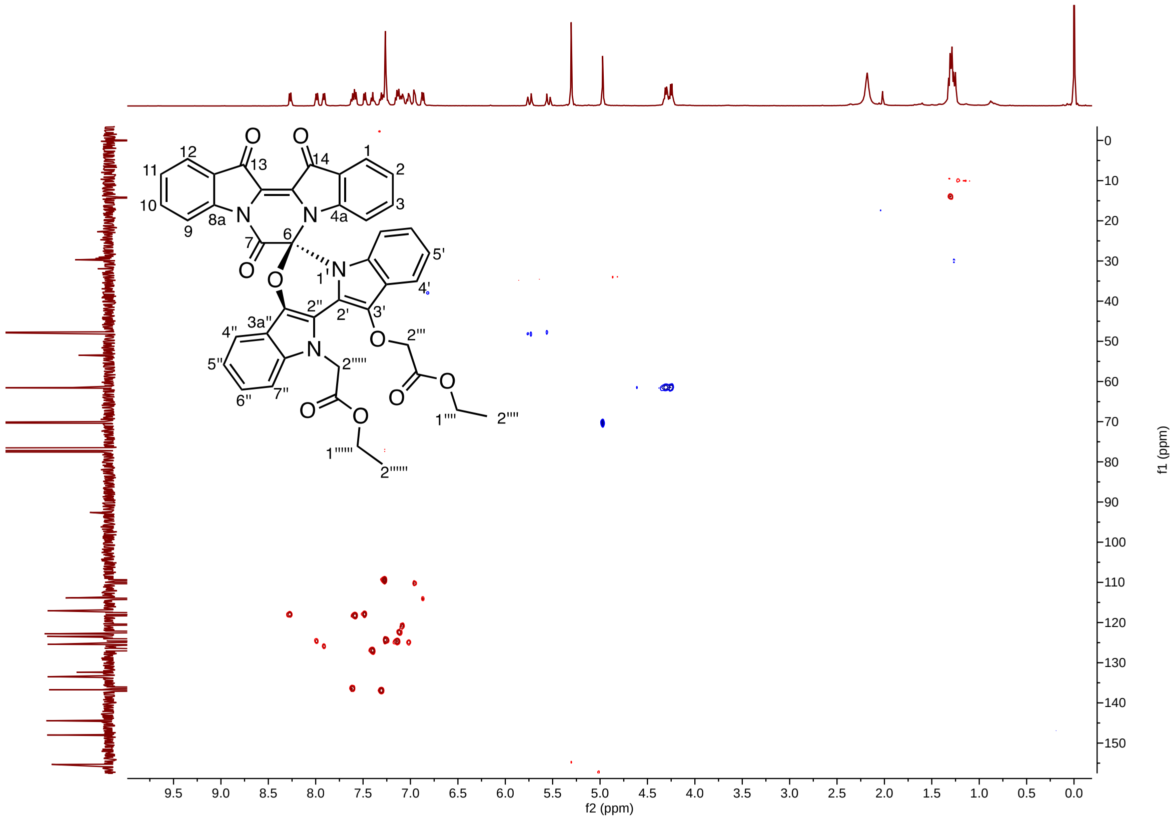

Figure S5: HSQC spectrum of the compound **17** recorded in $CDCl_3$ using 400 MHz NMR instrument.

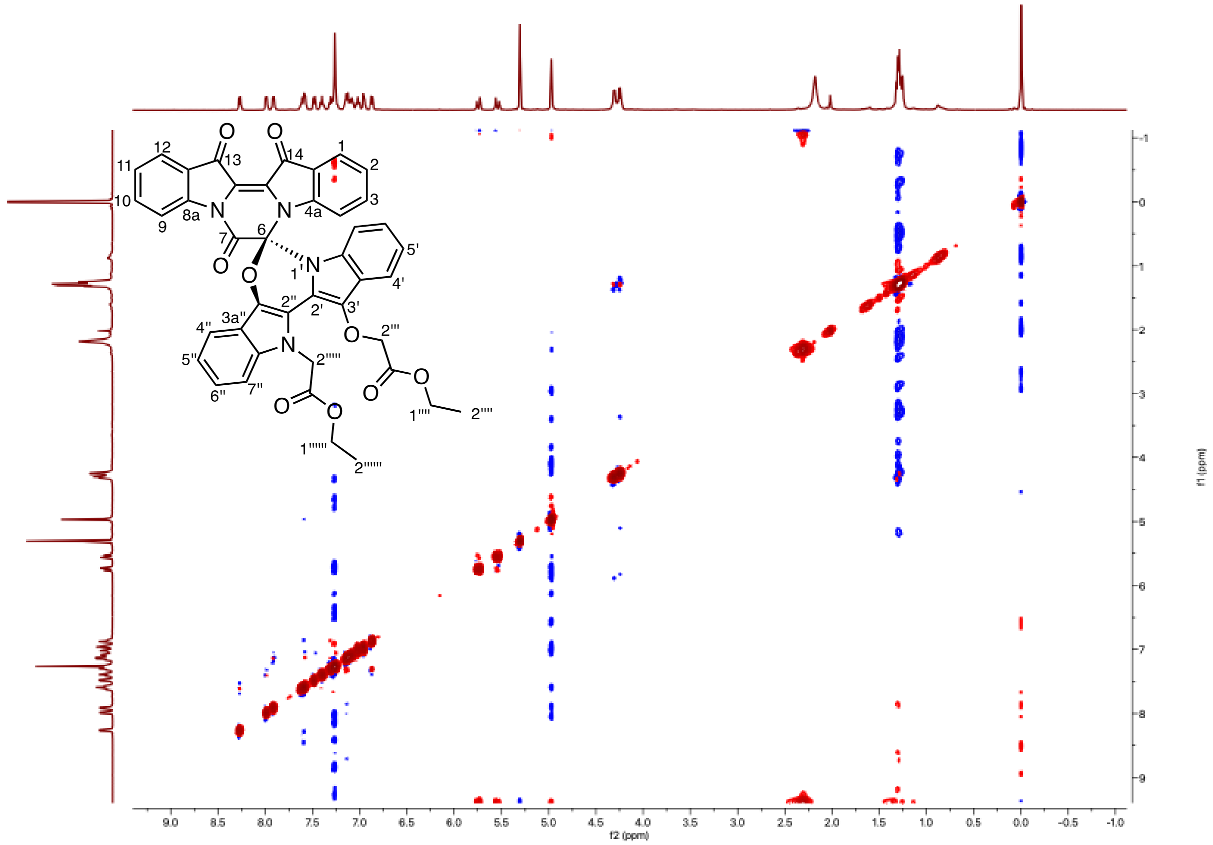

Figure S6: NOESY spectrum of the compound **17** recorded in $CDCl_3$ using 500 MHz NMR instrument.

NMR spectra of compound 18.

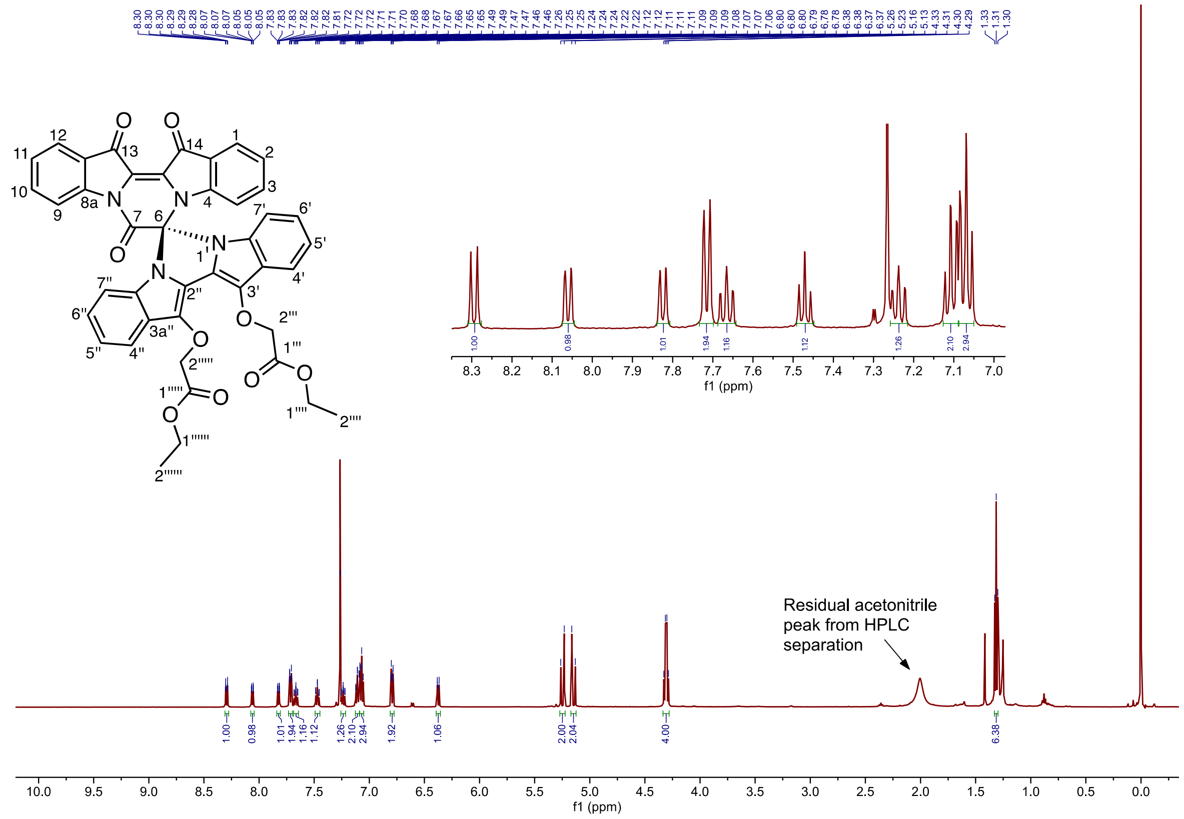

Figure S7: ^{1}H NMR spectrum of the compound **18** recorded in $CDCl_3$ using 500 MHz NMR instrument.

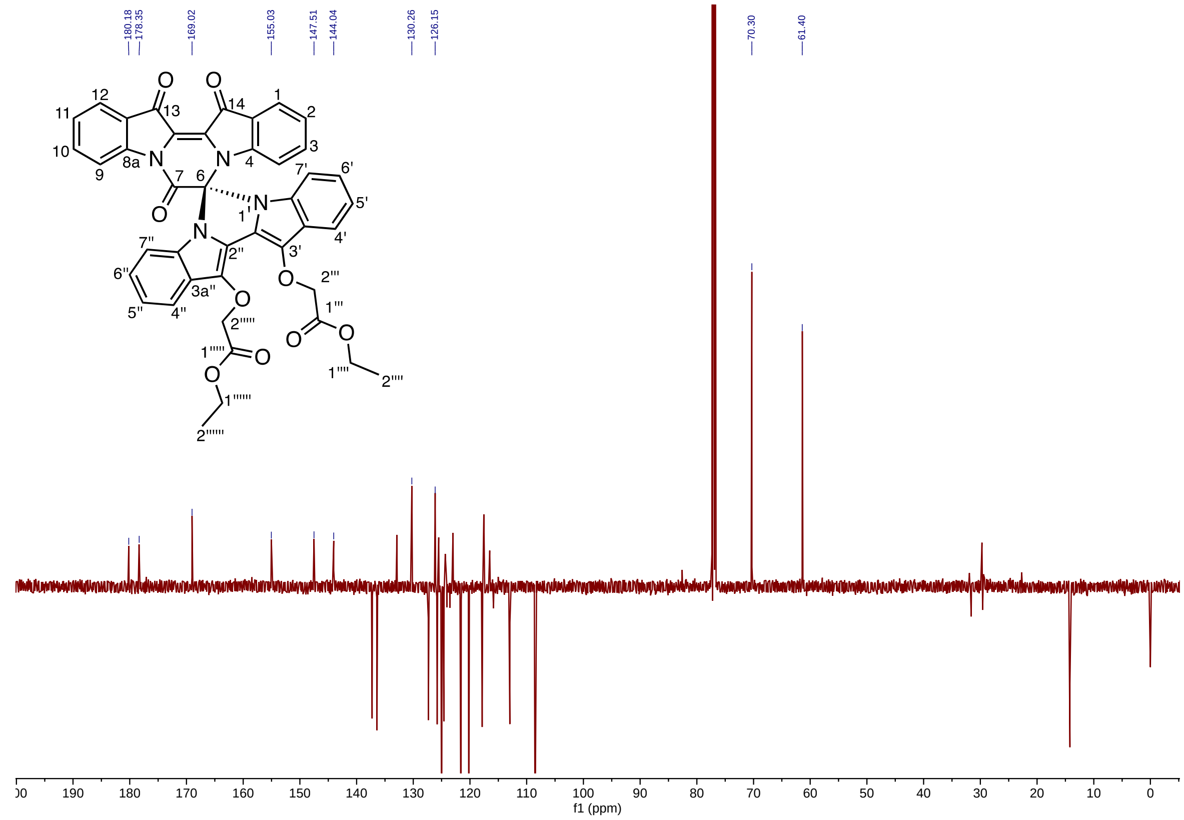

Figure S8: ^{13}C NMR spectrum of the compound **18** ($CDCl_3$, 500 MHz).

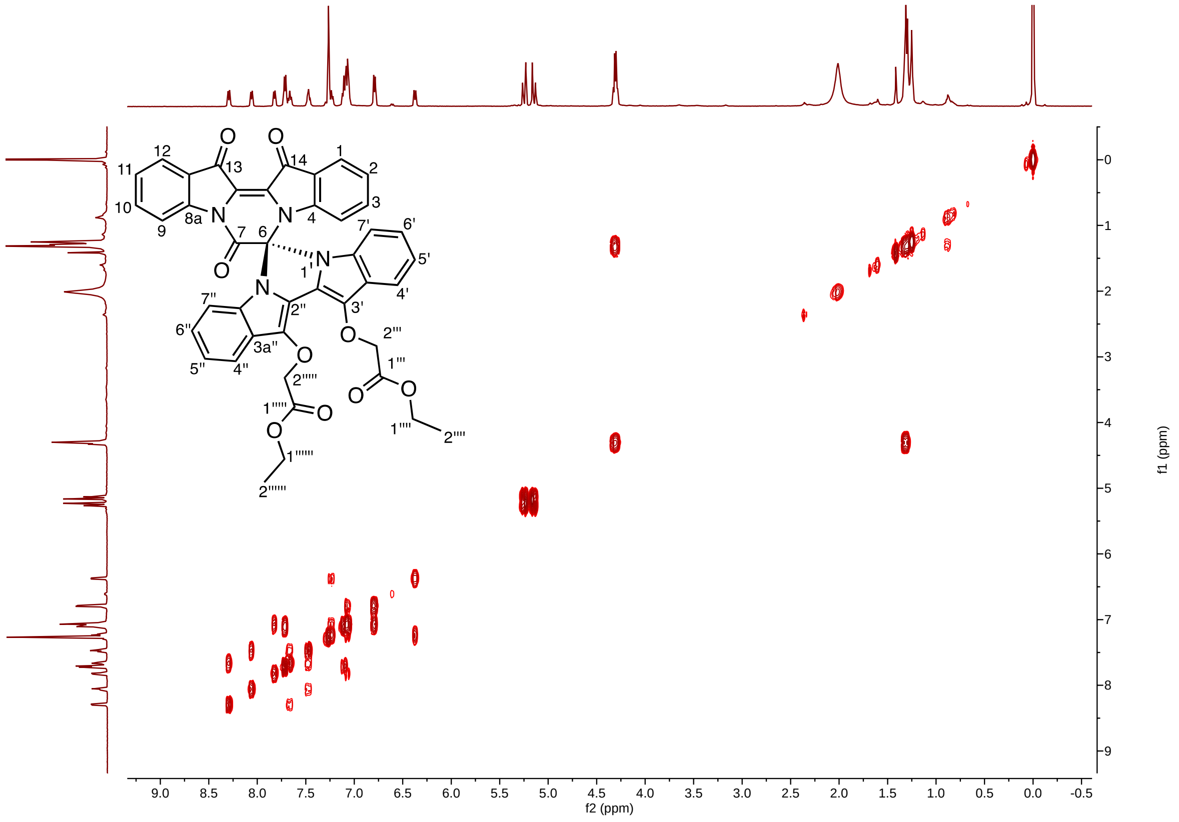

Figure S9: COSY spectrum of the compound **18** ($CDCl_3$, 500 MHz).

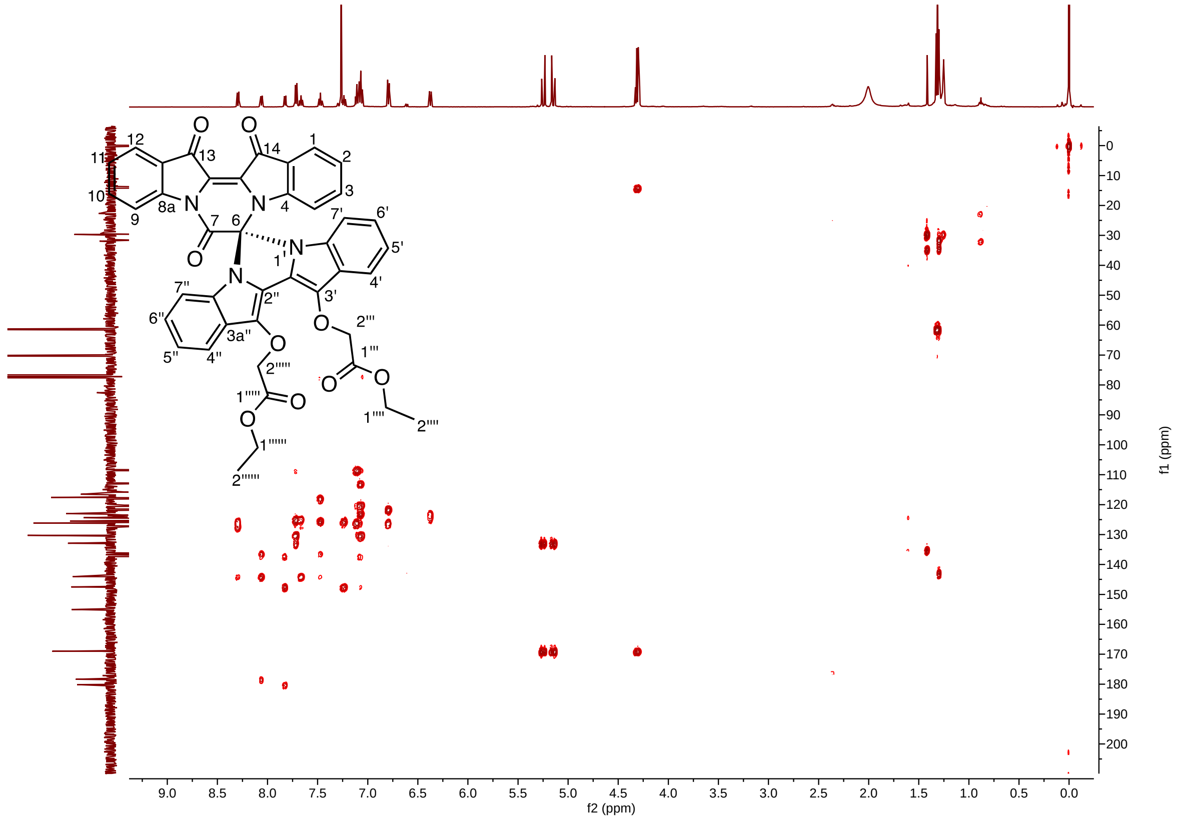

Figure S10: HMBC spectrum of the compound **18** recorded in $CDCl_3$ using 500 MHz NMR instrument.

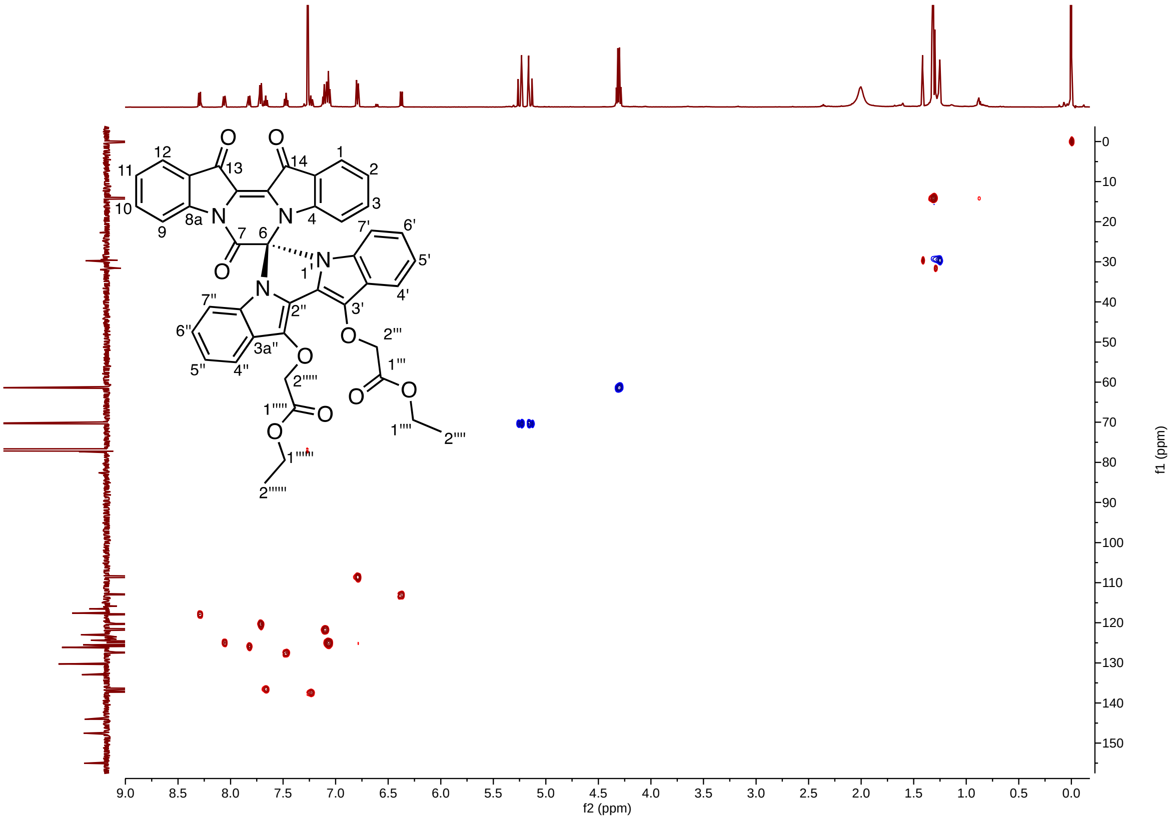

Figure S11: HSQC spectrum of the compound **18** recorded in $CDCl_3$ using 400 MHz NMR instrument.

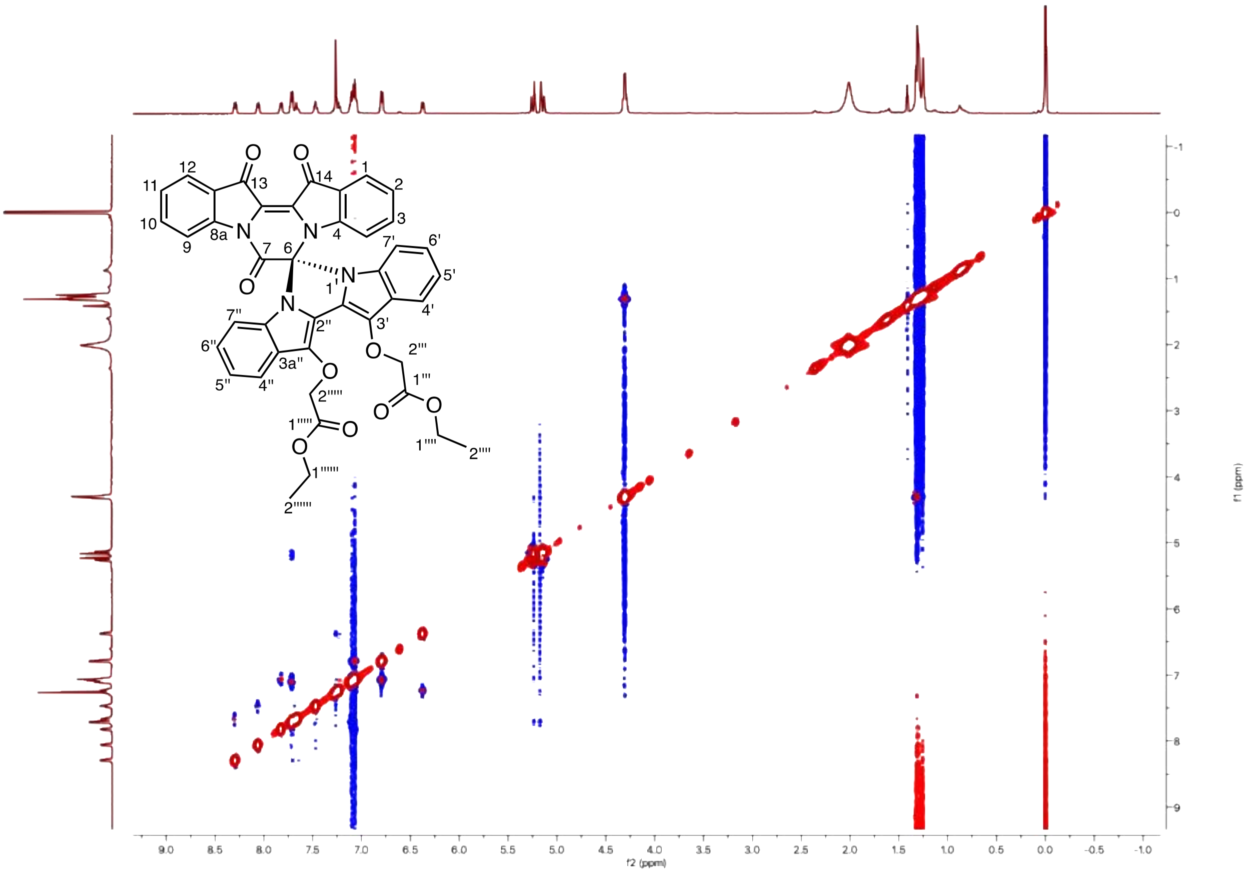

Figure S12: NOESY spectrum of the compound **18** recorded in $CDCl_3$ using 500 MHz NMR instrument.

NMR spectra of compound 19.

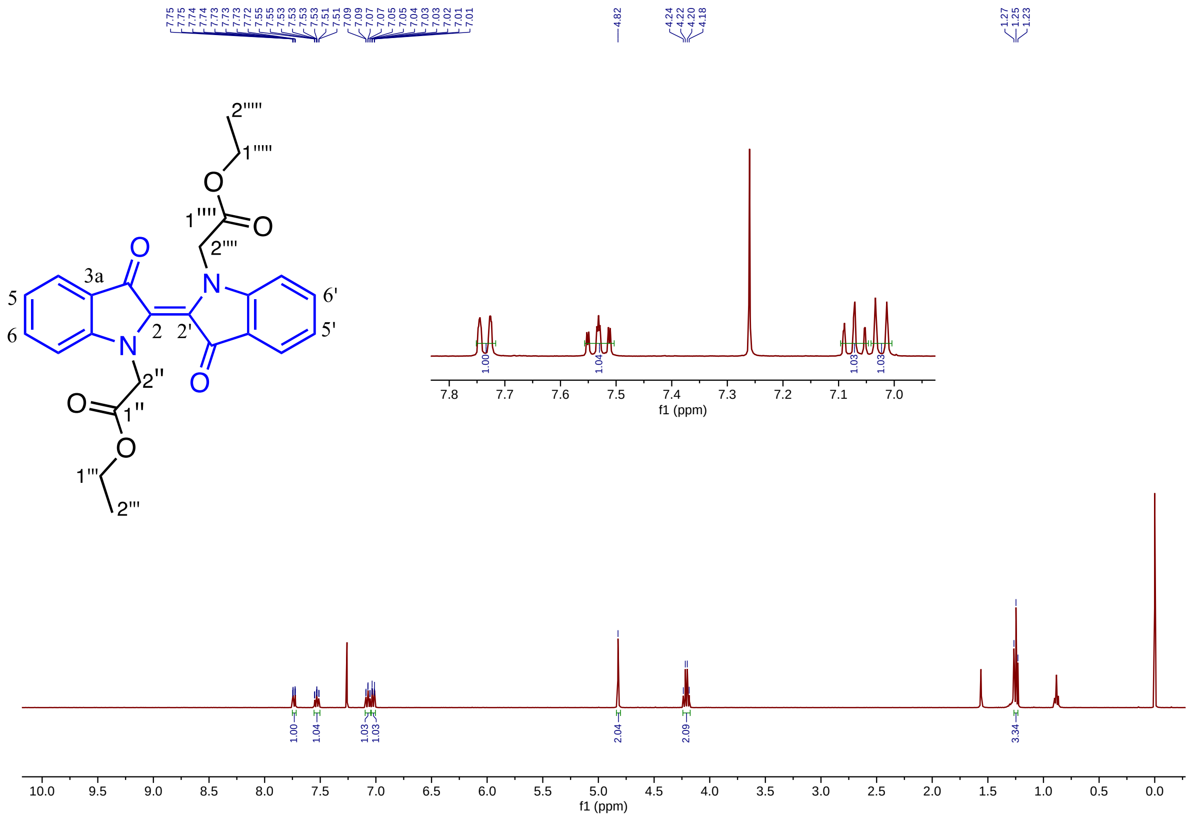

Figure S13: ^{1}H NMR spectrum of the compound **19** recorded in $CDCl_3$ using 400 MHz NMR instrument.

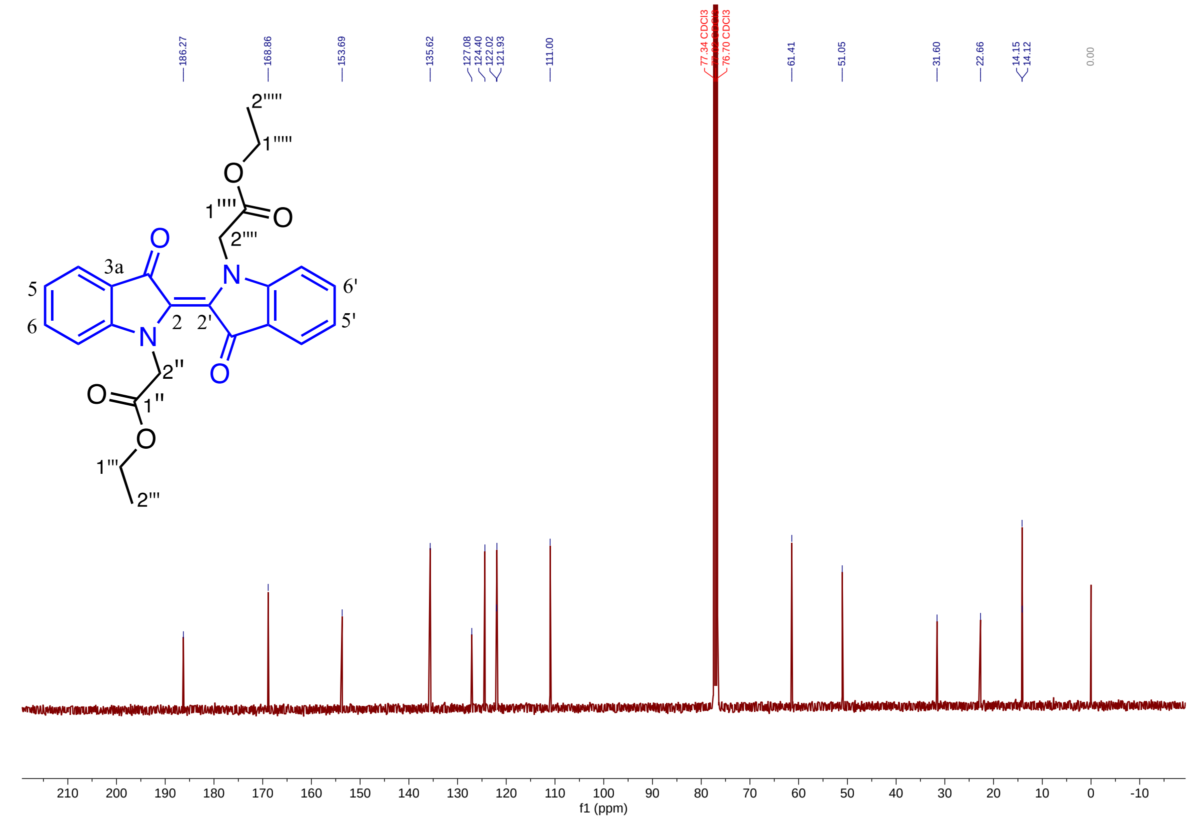

Figure S14: ^{13}C NMR spectrum of the compound **19** ($CDCl_3$, 500 MHz).

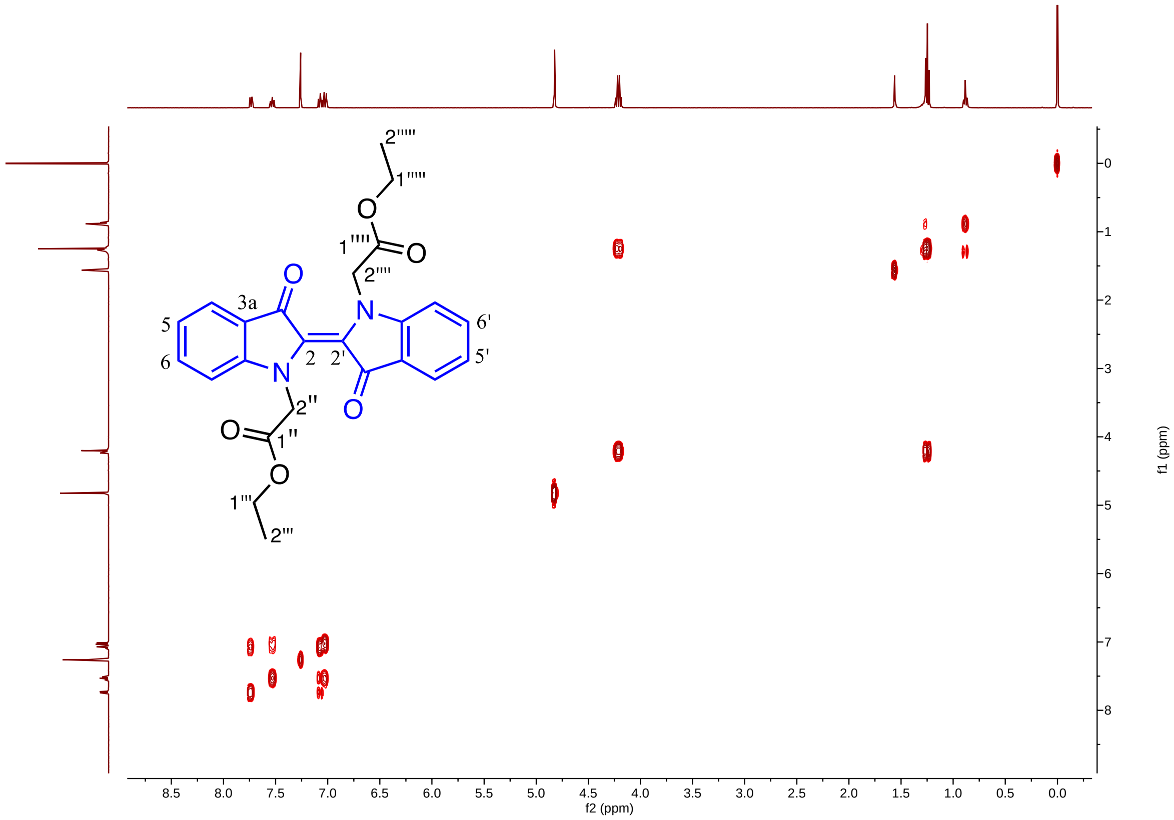

Figure S15: COSY spectrum of the compound **19** recorded in $CDCl_3$ using 400 MHz NMR instrument.

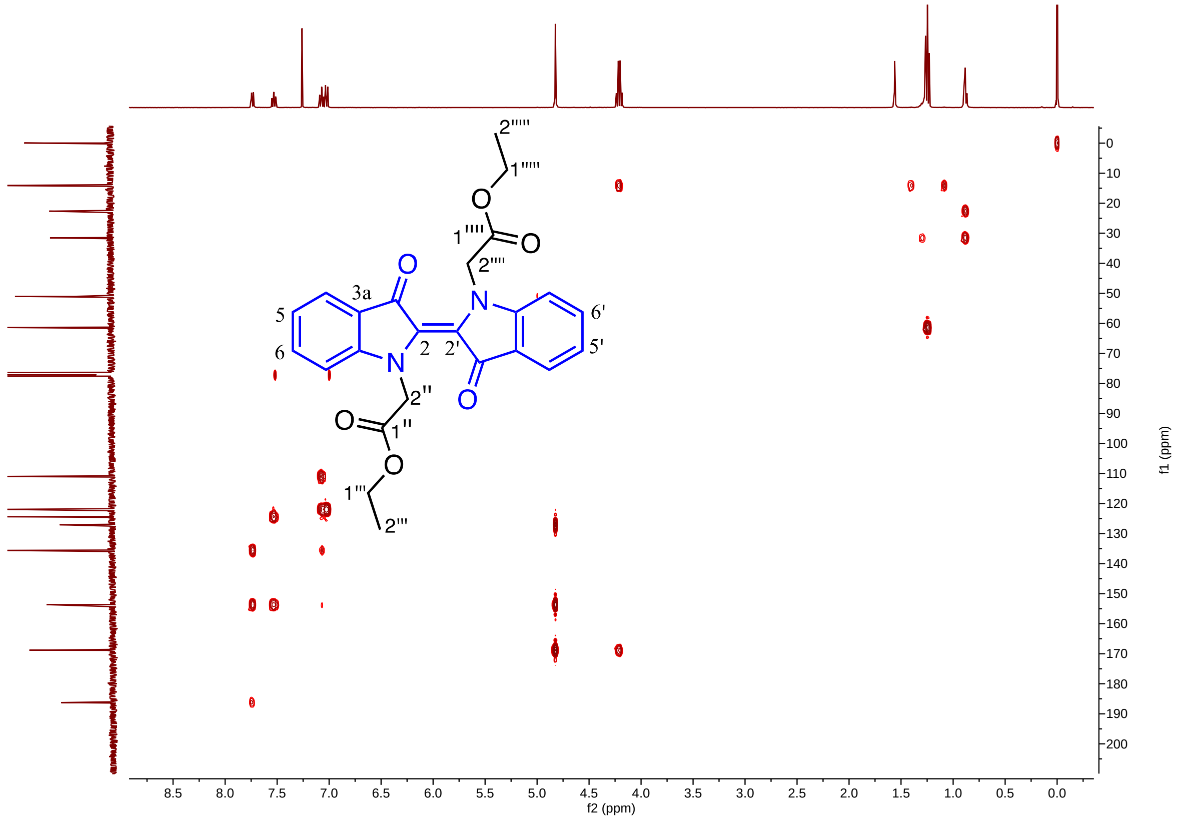

Figure S16: HMBC spectrum of the compound **19** recorded in $CDCl_3$ using 400 MHz NMR instrument.

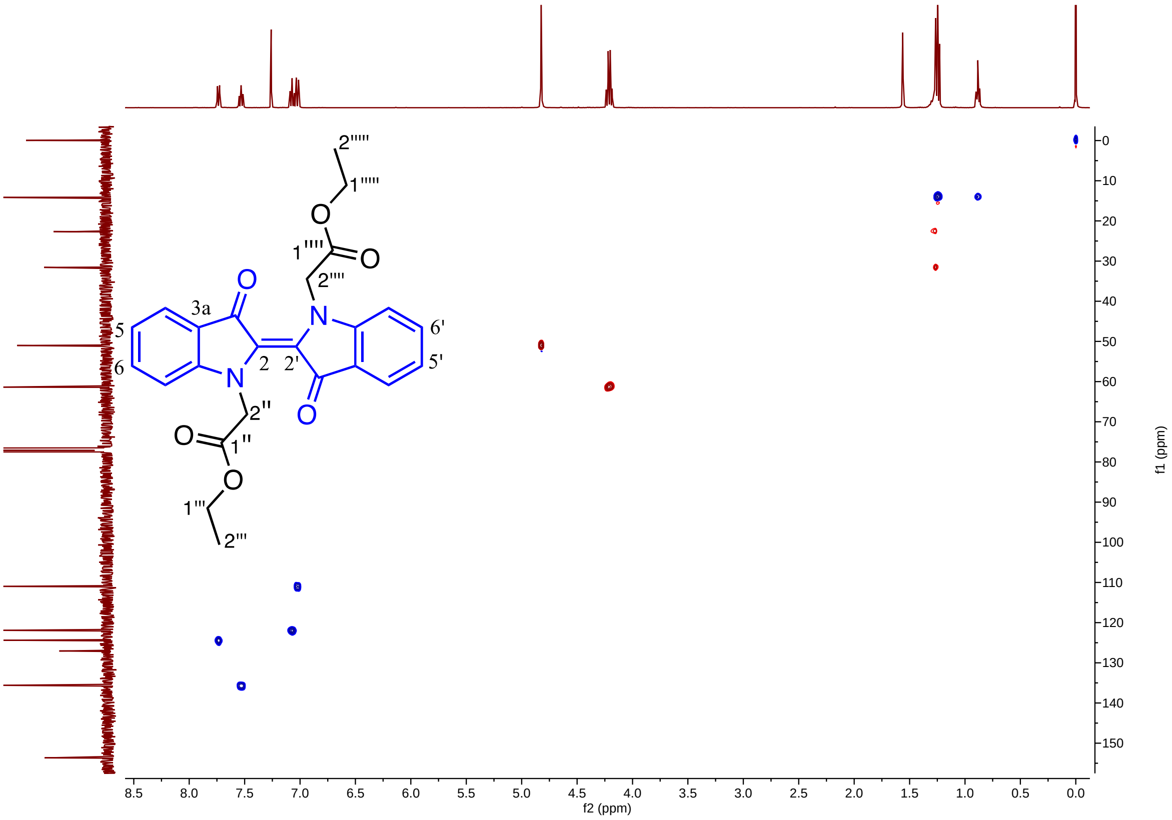

Figure S17: HSQC spectrum of the compound **19** recorded in $CDCl_3$ using 400 MHz NMR instrument.

NMR spectra of compound 20.

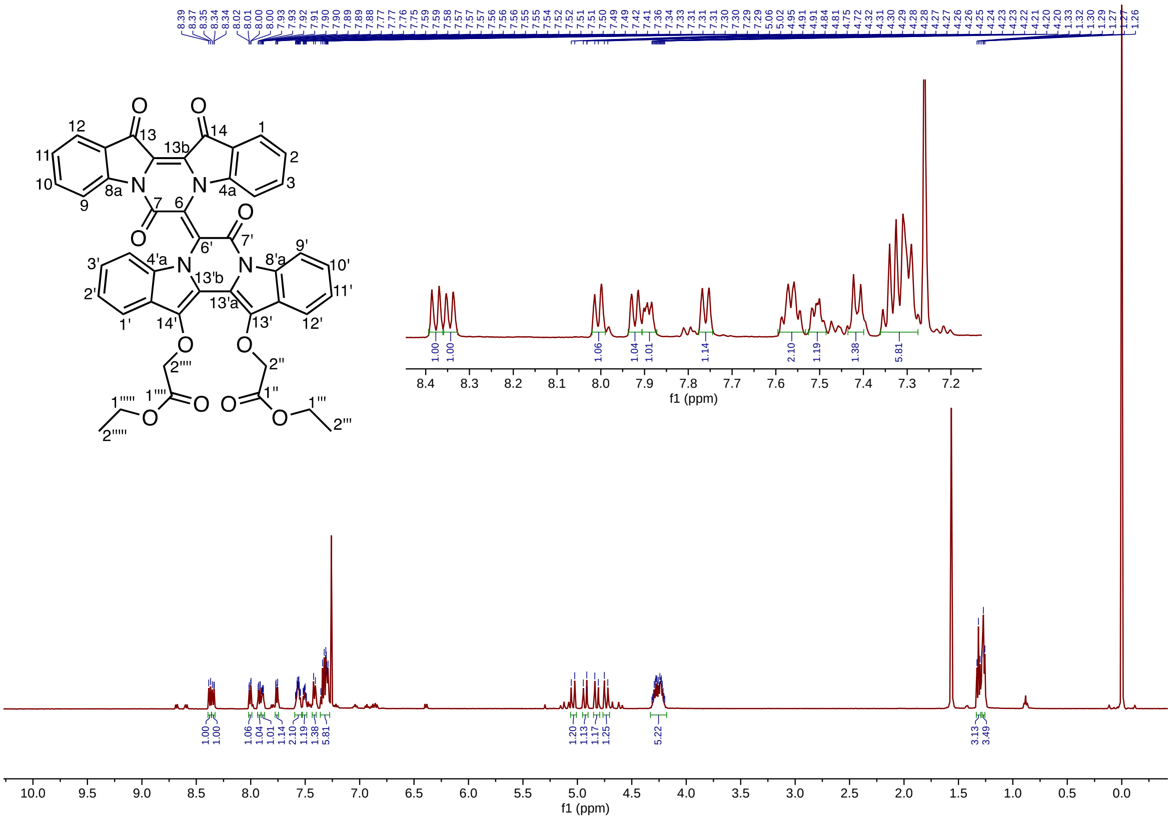

Figure S18: ^{1}H NMR spectrum of the compound **20** ($CDCl_3$, 500 MHz).

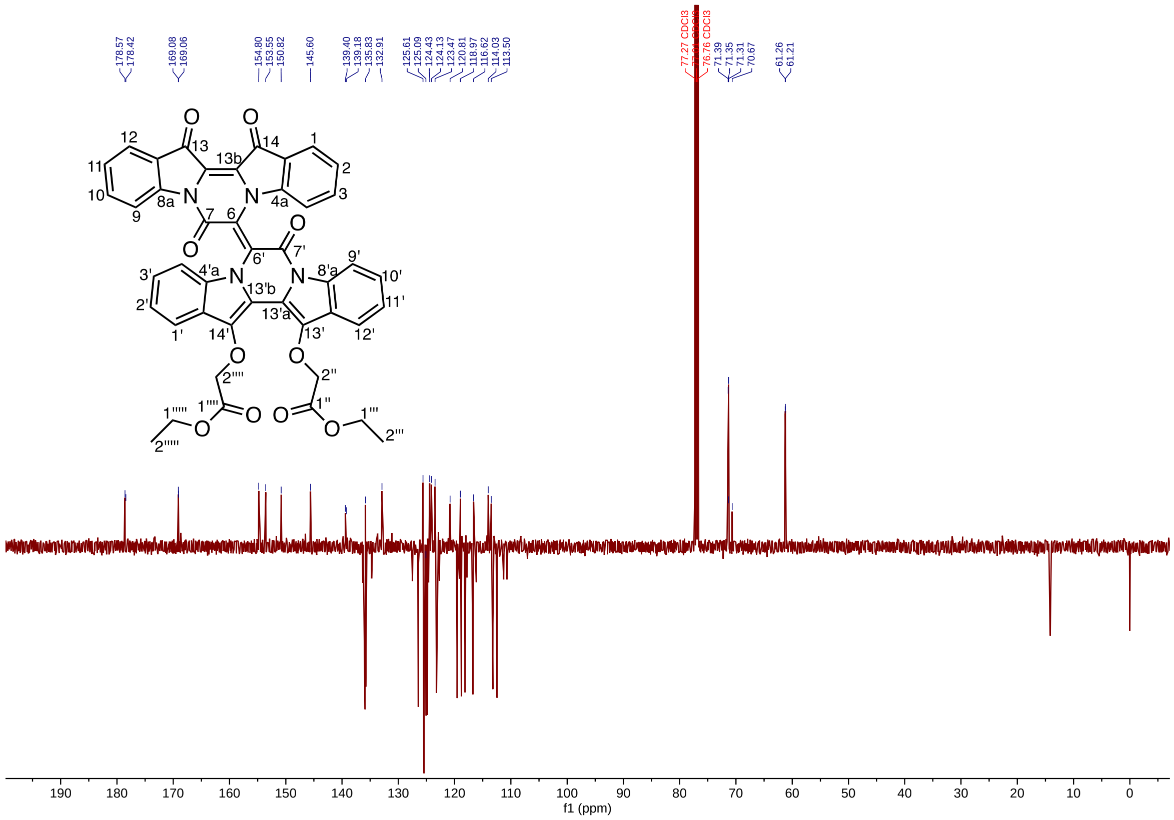

Figure S19: DEPT NMR spectrum of the compound **20** recorded in $CDCl_3$ using 125 MHz NMR instrument.

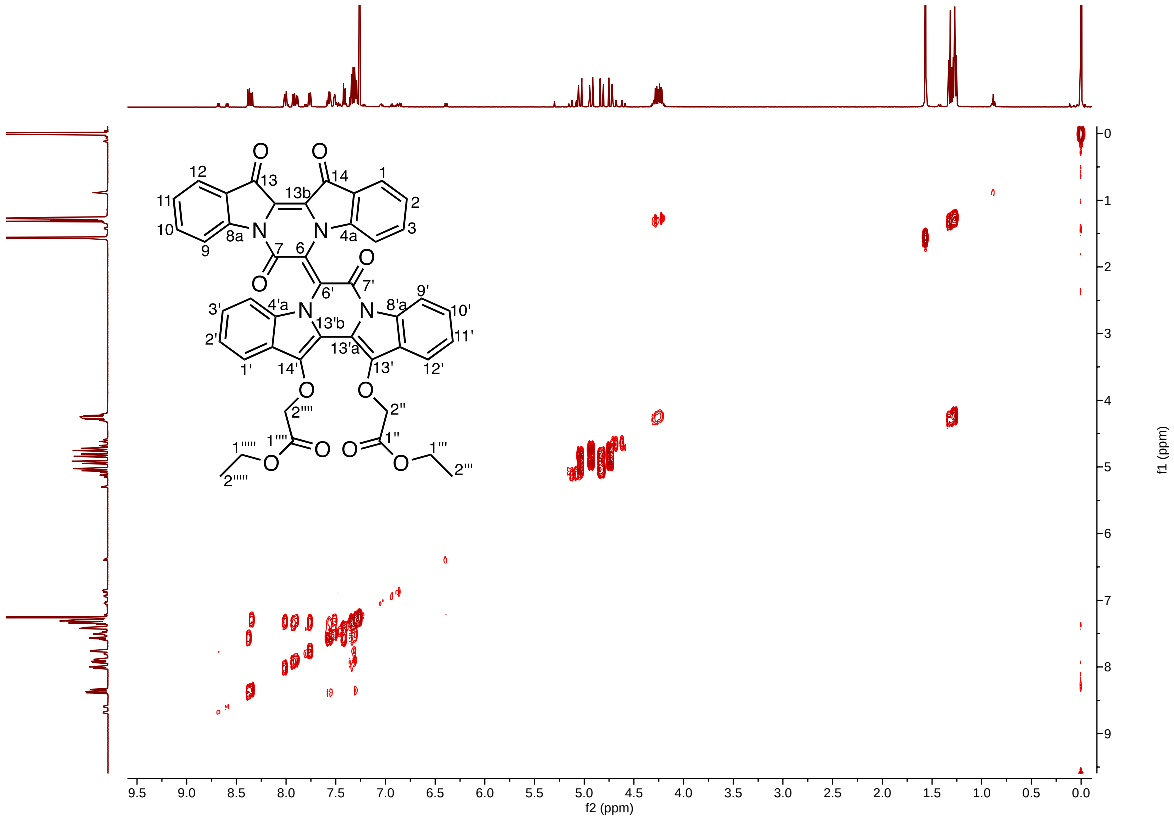

Figure S20: COSY spectrum of the compound **20** ($CDCl_3$, 500 MHz).

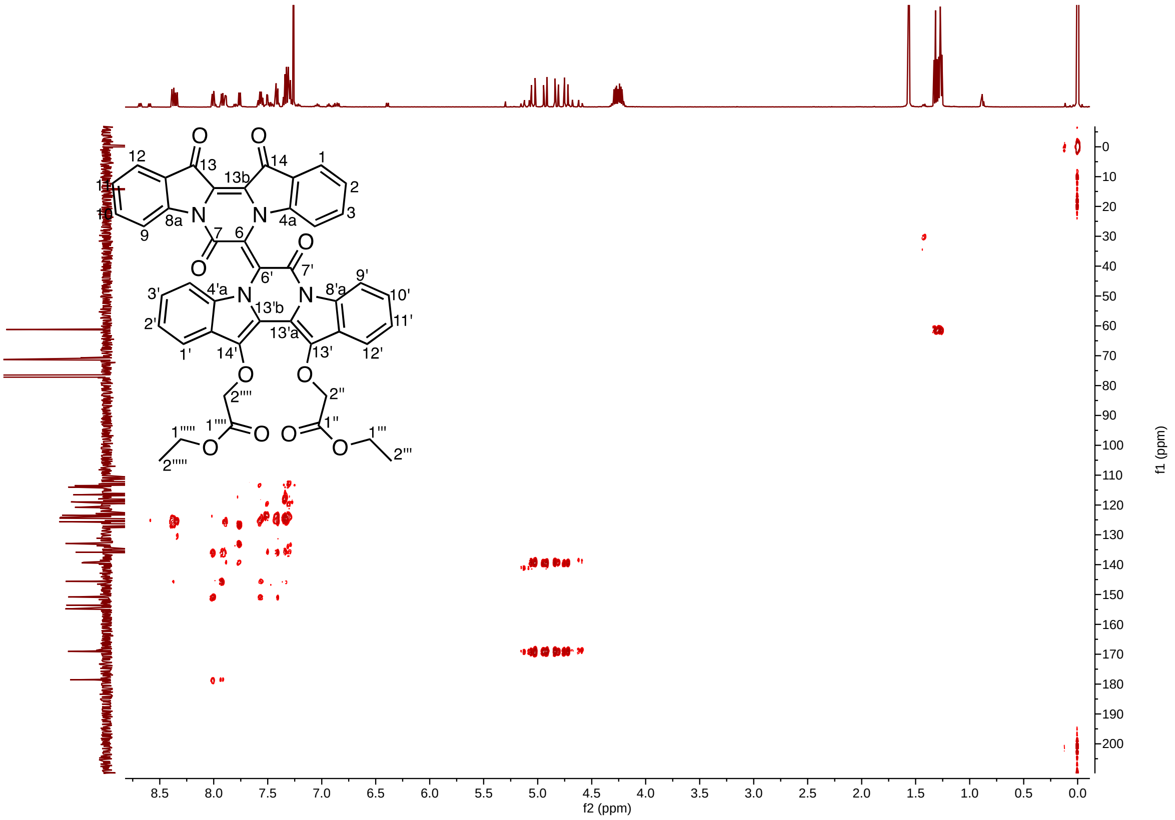

Figure S21: HMBC spectrum of the compound **20** recorded in $CDCl_3$ using 500 MHz NMR instrument.

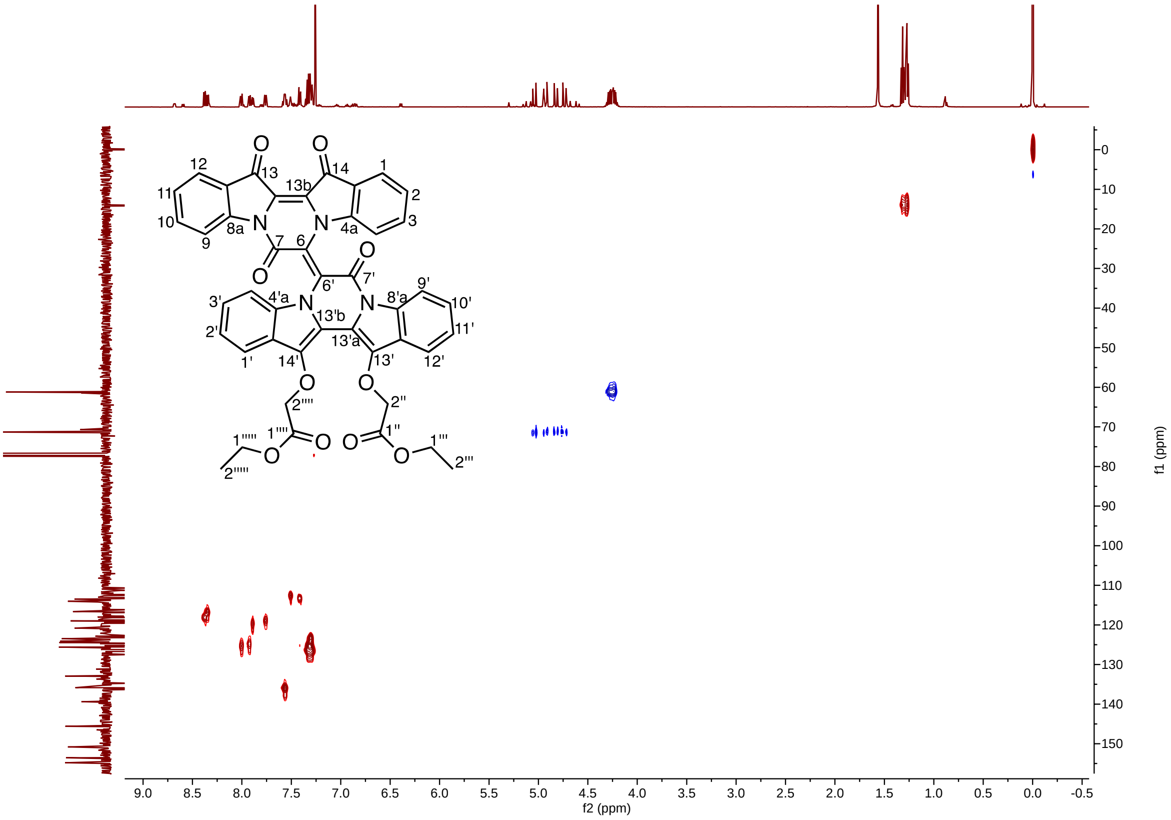

Figure S22: HSQC spectrum of the compound **20** recorded in $CDCl_3$ using 400 MHz NMR instrument.

NMR spectra of compound 21.

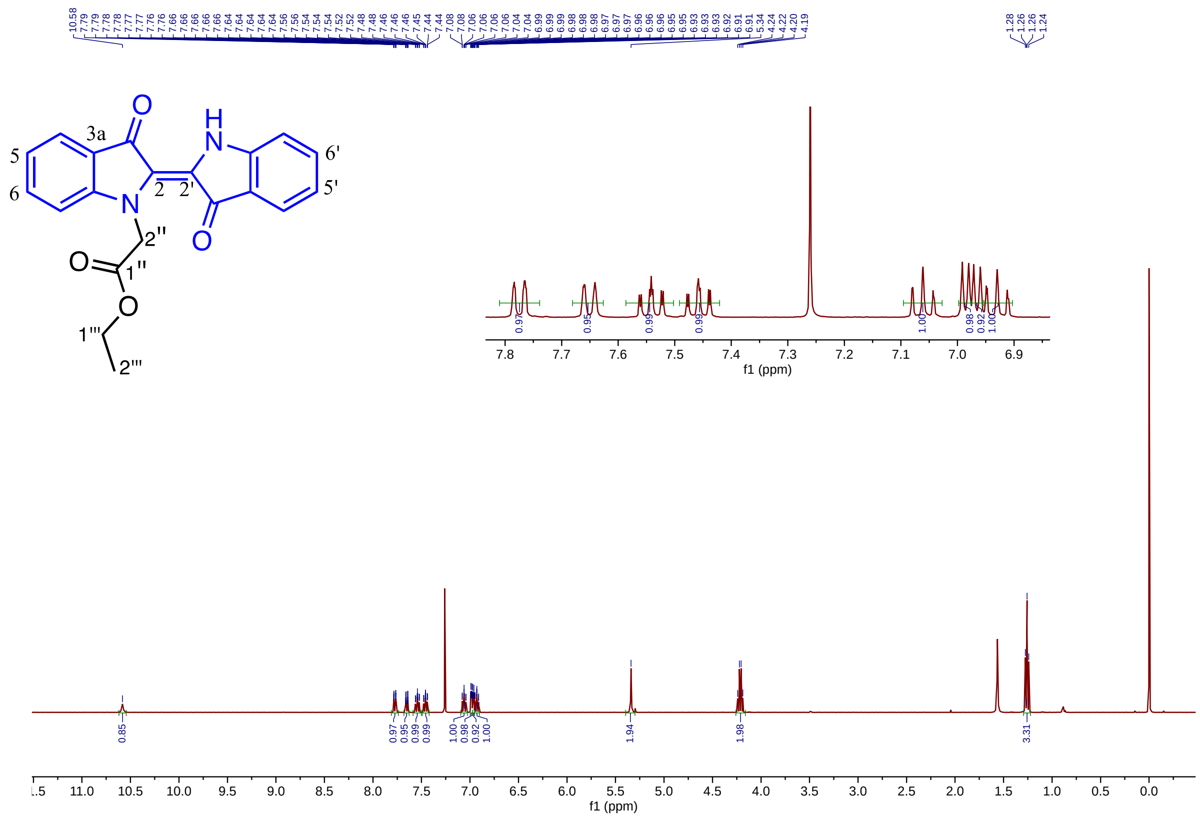

Figure S23: ^{1}H NMR spectrum of the compound **21** ($CDCl_3$, 500 MHz).

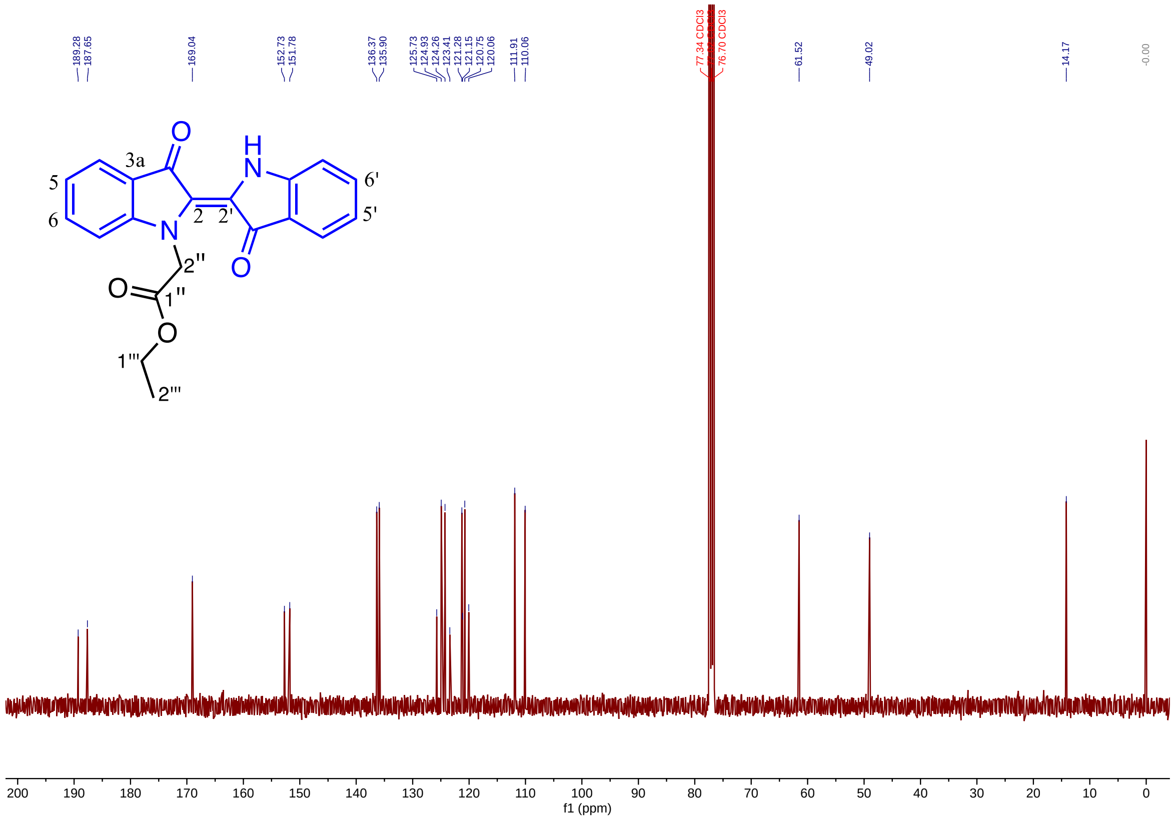

Figure S24: ^{13}C NMR spectrum of the compound **21** recorded in $CDCl_3$ using 100 MHz NMR instrument.

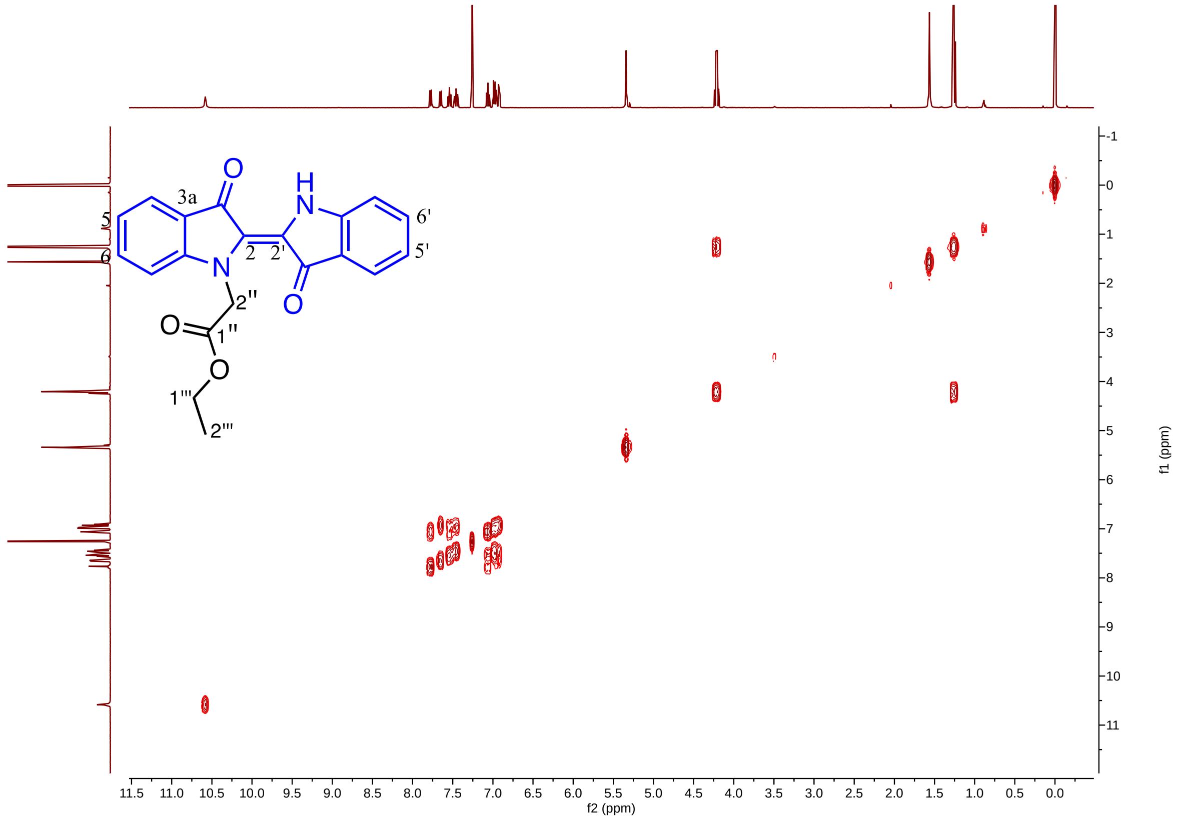

Figure S25: COSY spectrum of the compound **21** ($CDCl_3$, 500 MHz).

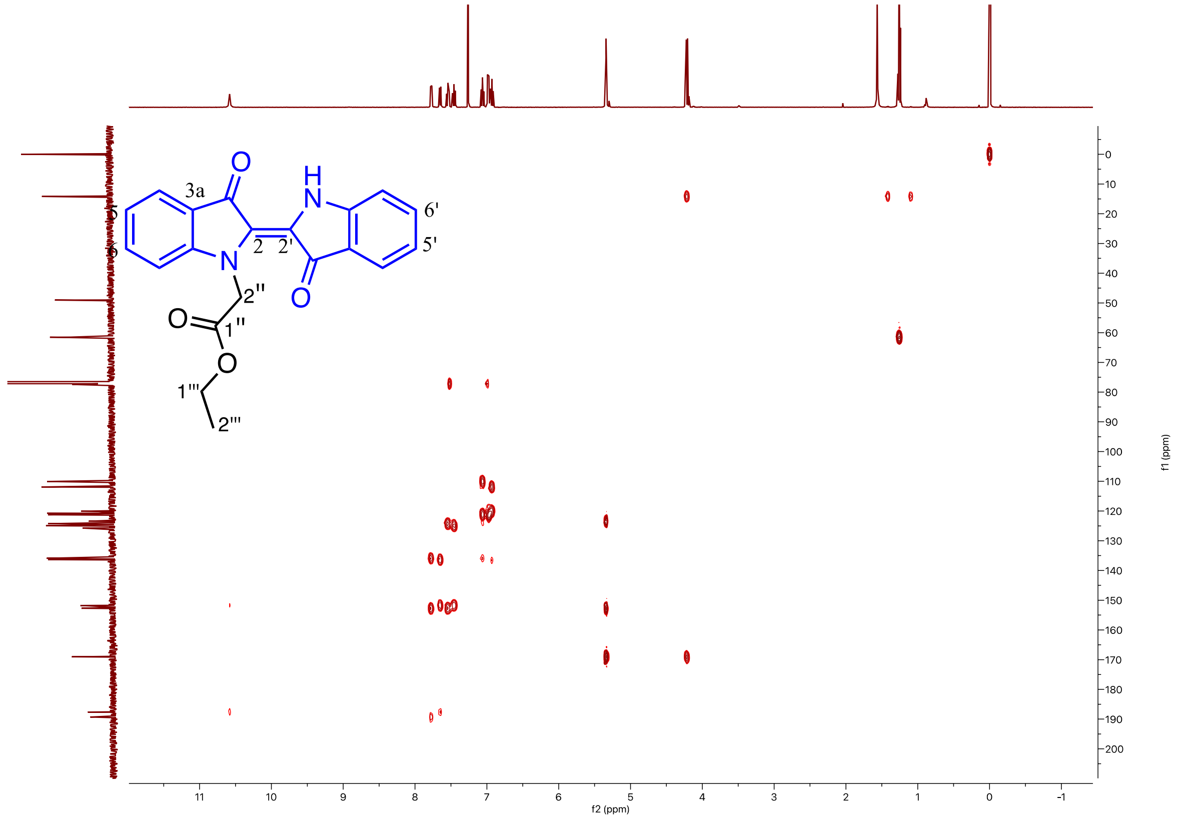

Figure S26: HMBC spectrum of the compound **21** recorded in $CDCl_3$ using 400 MHz NMR instrument.

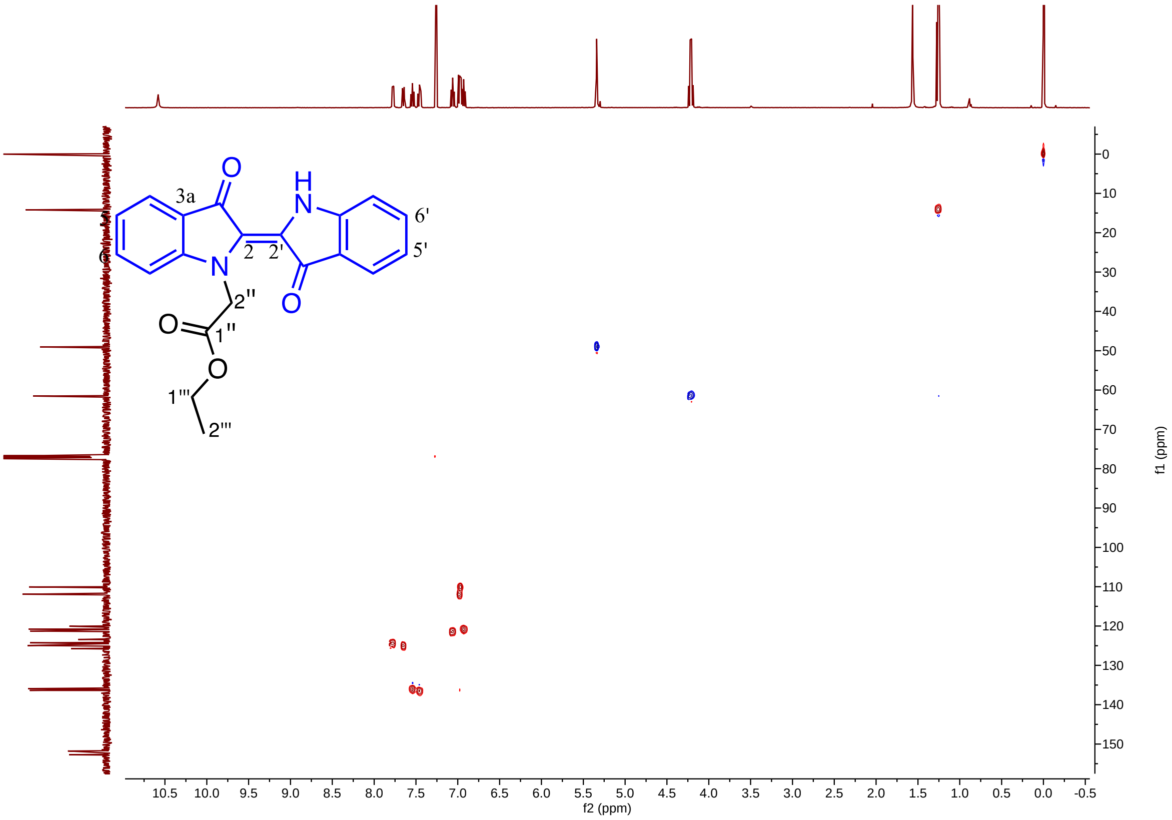

Figure S27: HSQC spectrum of the compound **21** recorded in $CDCl_3$ using 400 MHz NMR instrument.

NMR spectra of compound 22.

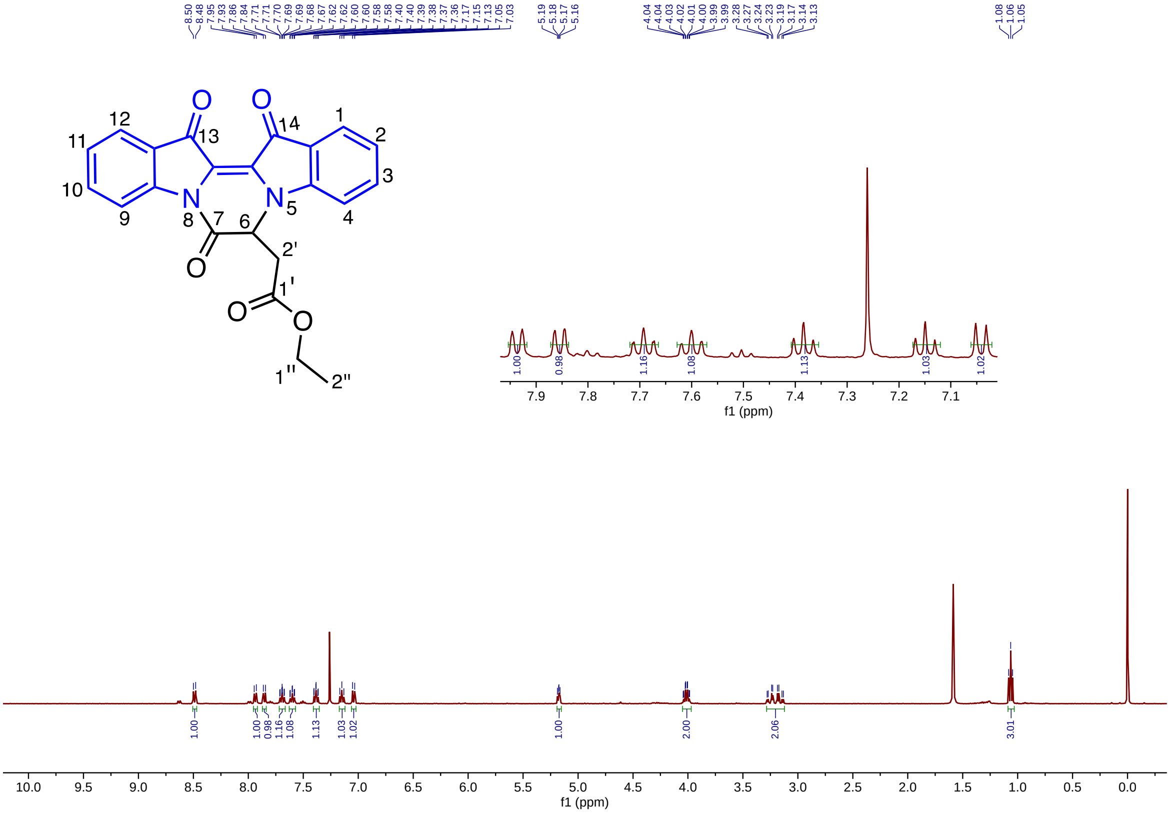

Figure S28: ^{1}H NMR spectrum of the compound **22** ($CDCl_3$, 500 MHz).

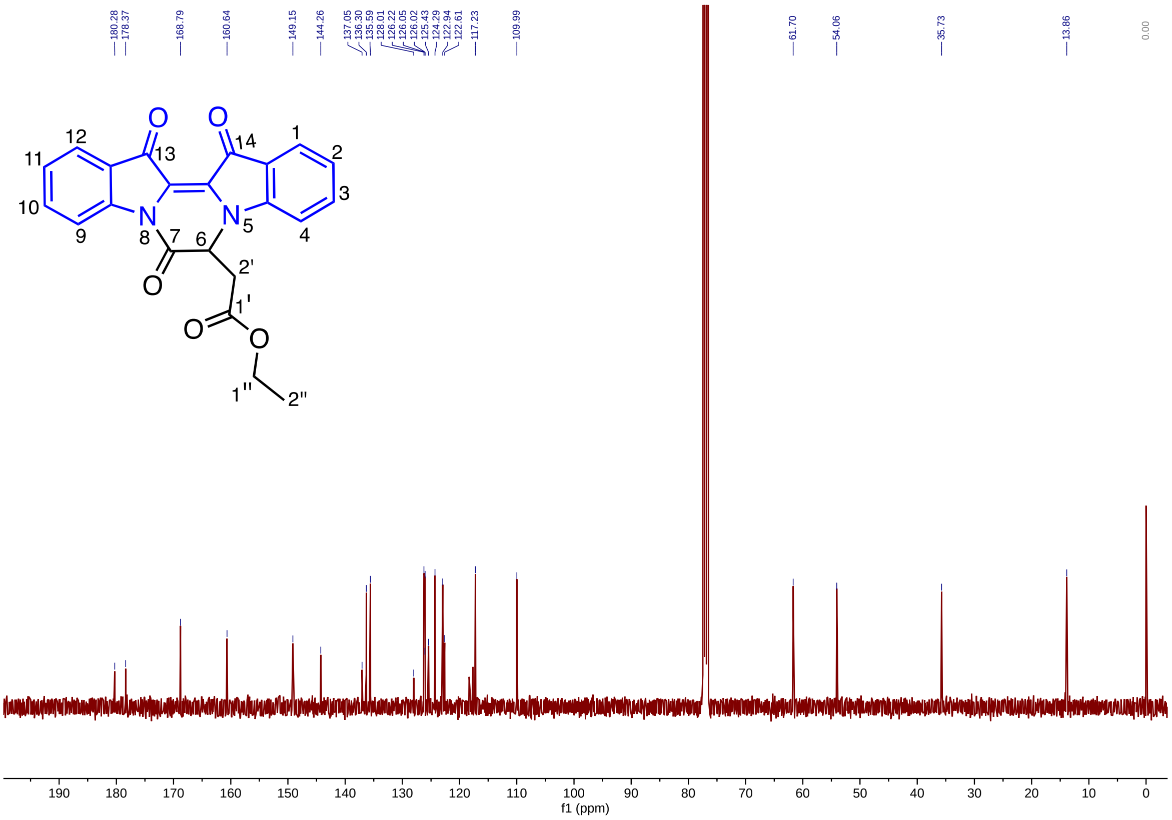

Figure S29: ^{13}C NMR spectrum of the compound **22** recorded in $CDCl_3$ using 100 MHz NMR instrument.

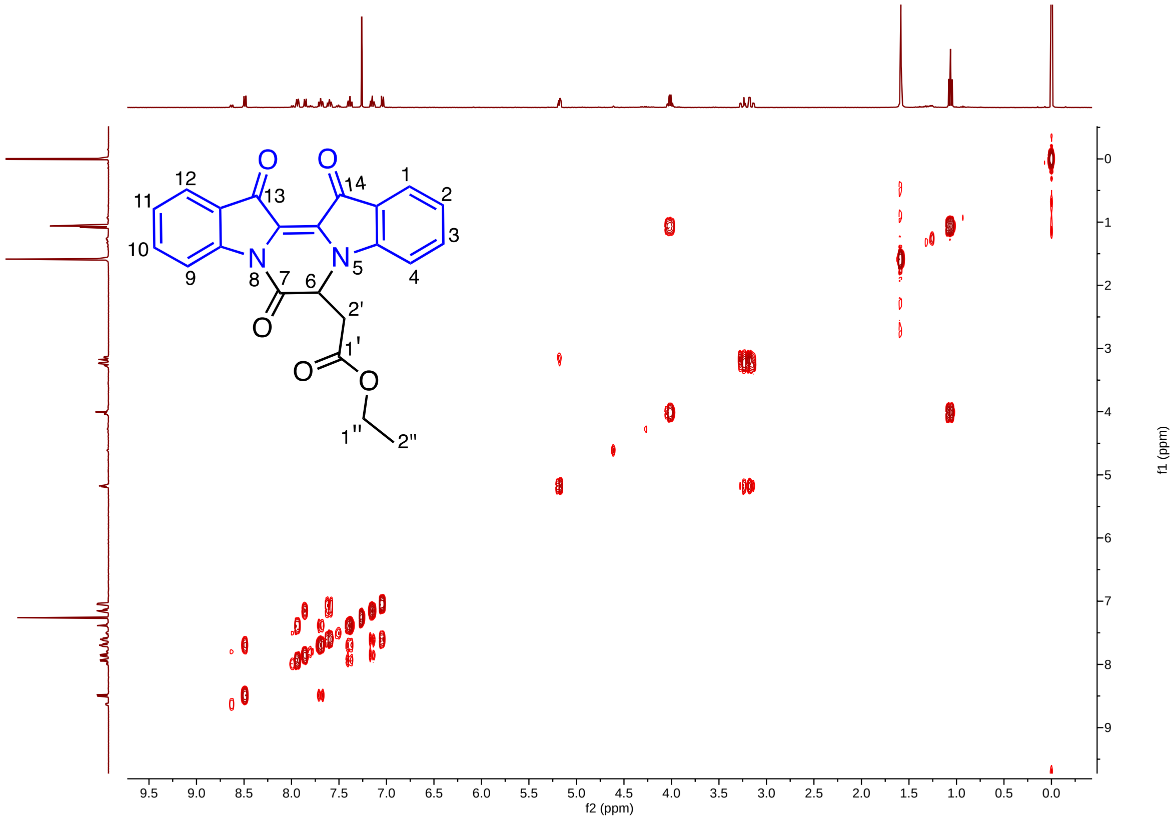

Figure S30: COSY spectrum of the compound **22** ($CDCl_3$, 500 MHz).

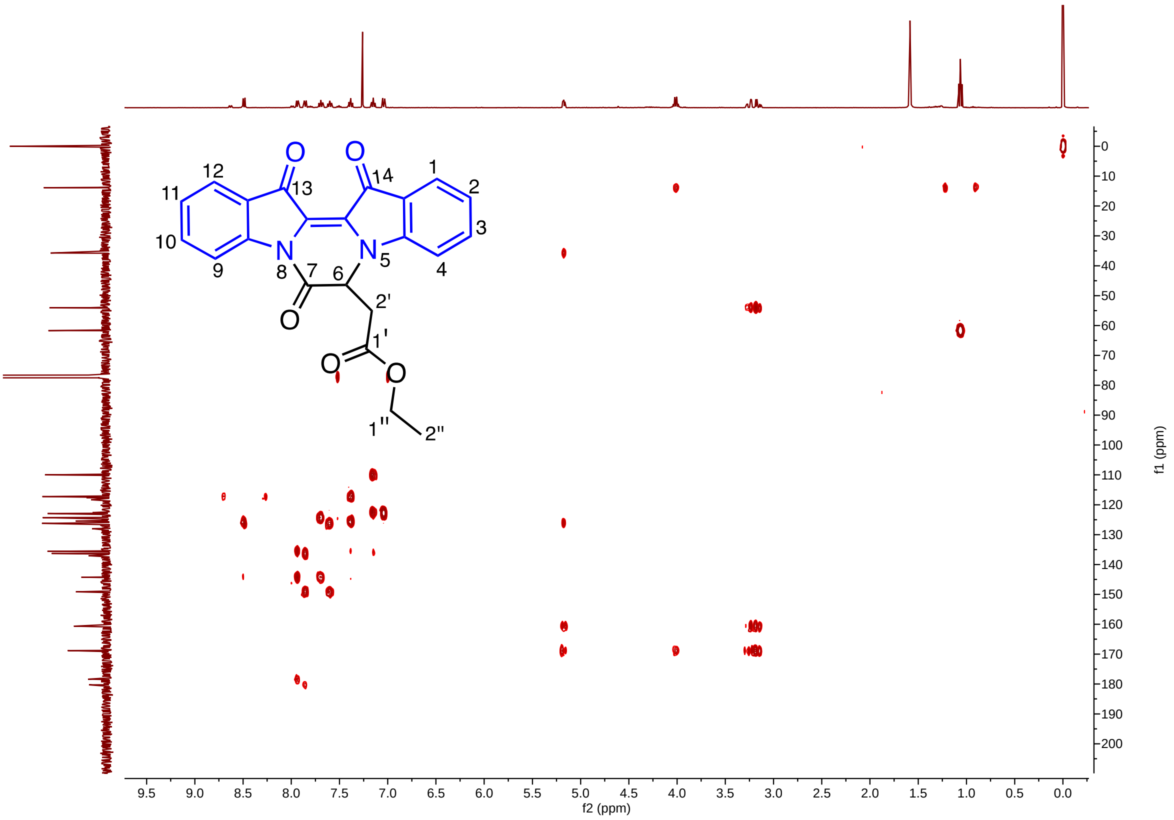

Figure S31: HMBC spectrum of the compound **22** recorded in $CDCl_3$ using 400 MHz NMR instrument.

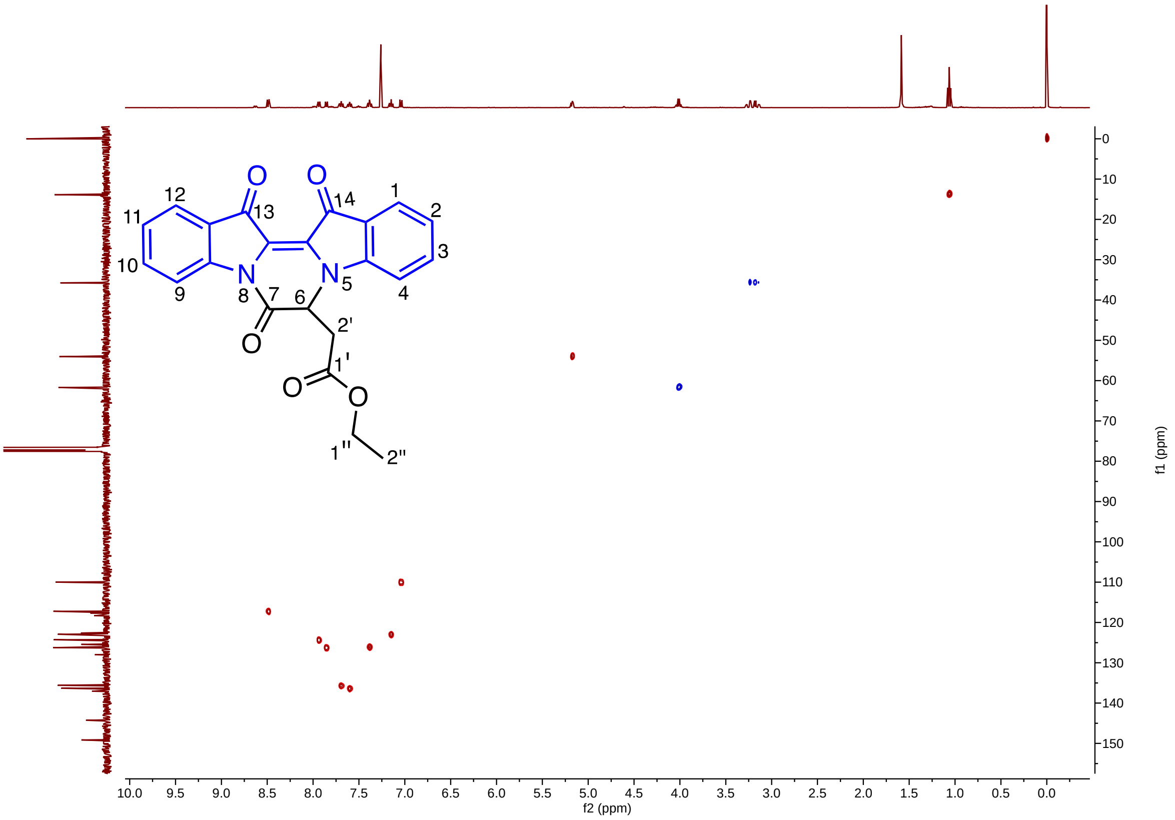

Figure S32: HSQC spectrum of the compound **22** recorded in $CDCl_3$ using 400 MHz NMR instrument.

NMR spectra of compound 23.

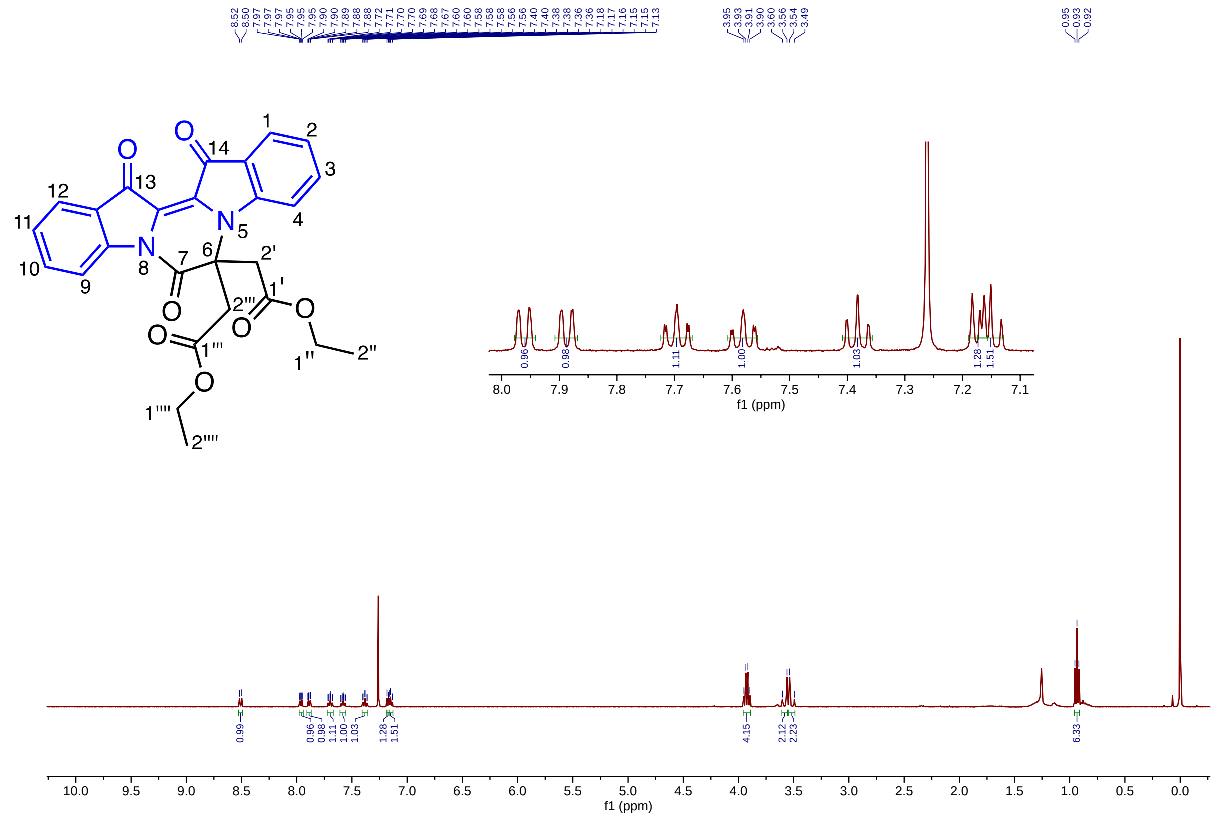

Figure S33: ^{1}H NMR spectrum of the compound **23** ($CDCl_3$, 400 MHz).

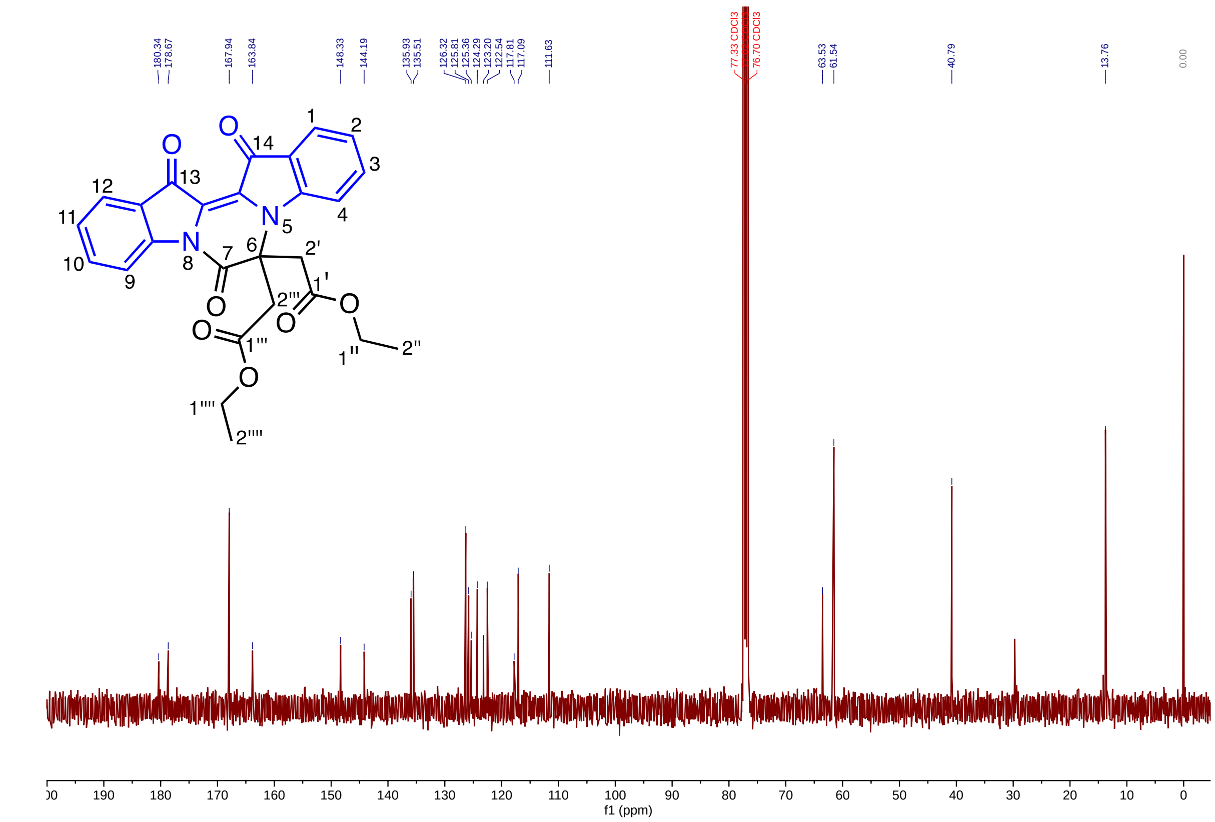

Figure S34: ^{13}C NMR spectrum of the compound **23** recorded in $CDCl_3$ using 100 MHz NMR instrument.

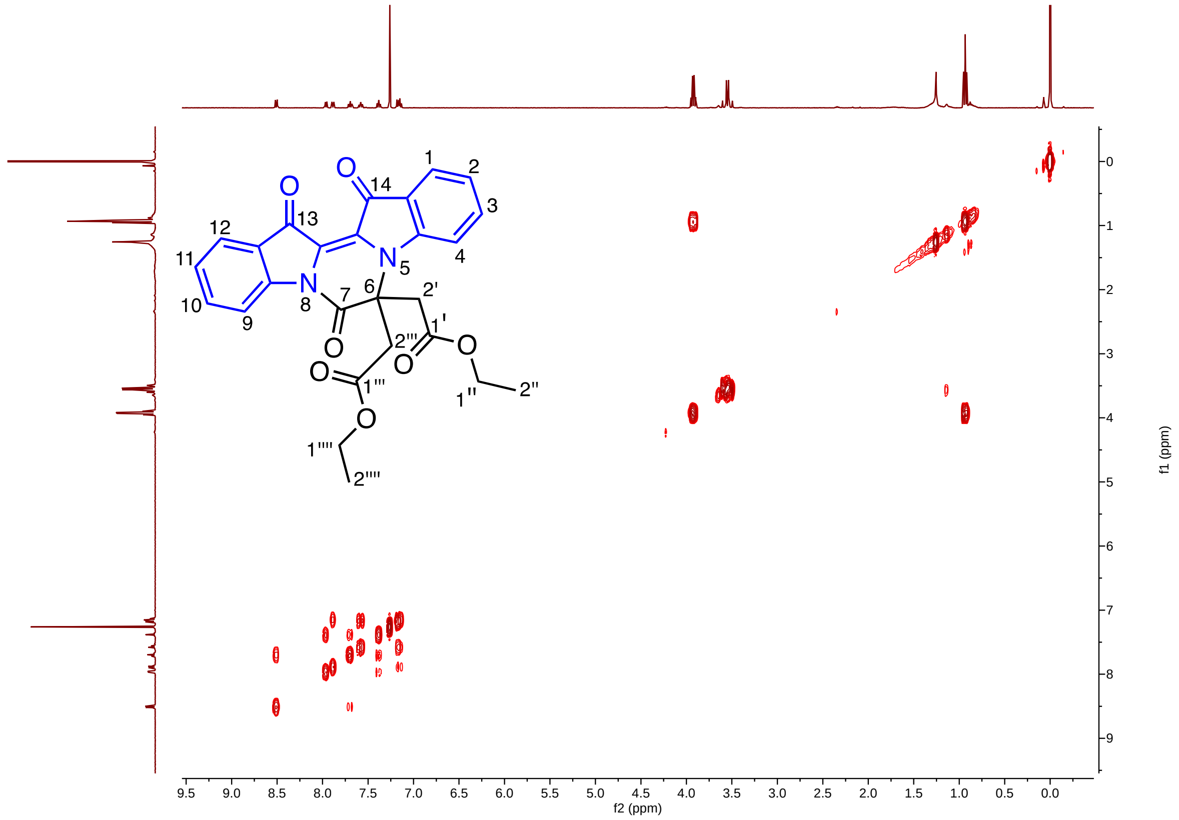

Figure S35: COSY spectrum of the compound **23** ($CDCl_3$, 400 MHz).

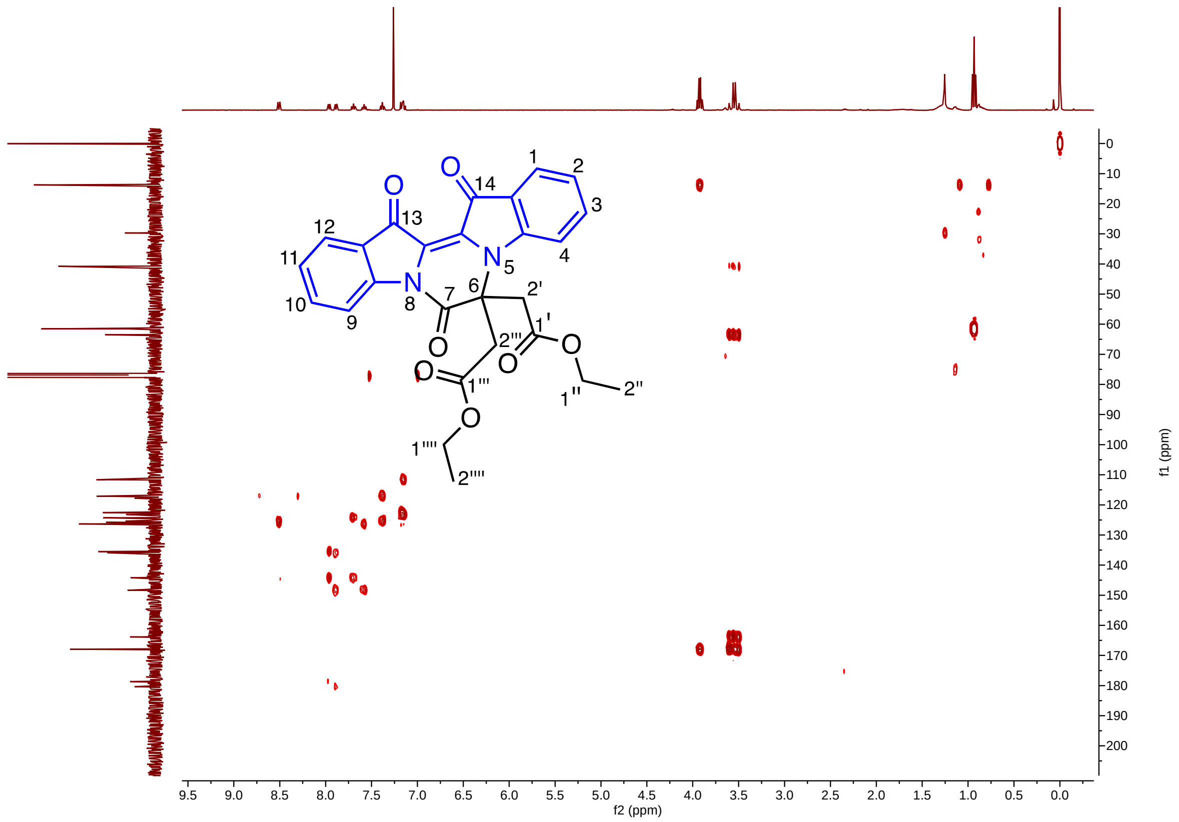

Figure S36: HMBC spectrum of the compound **23** recorded in $CDCl_3$ using 400 MHz NMR instrument.

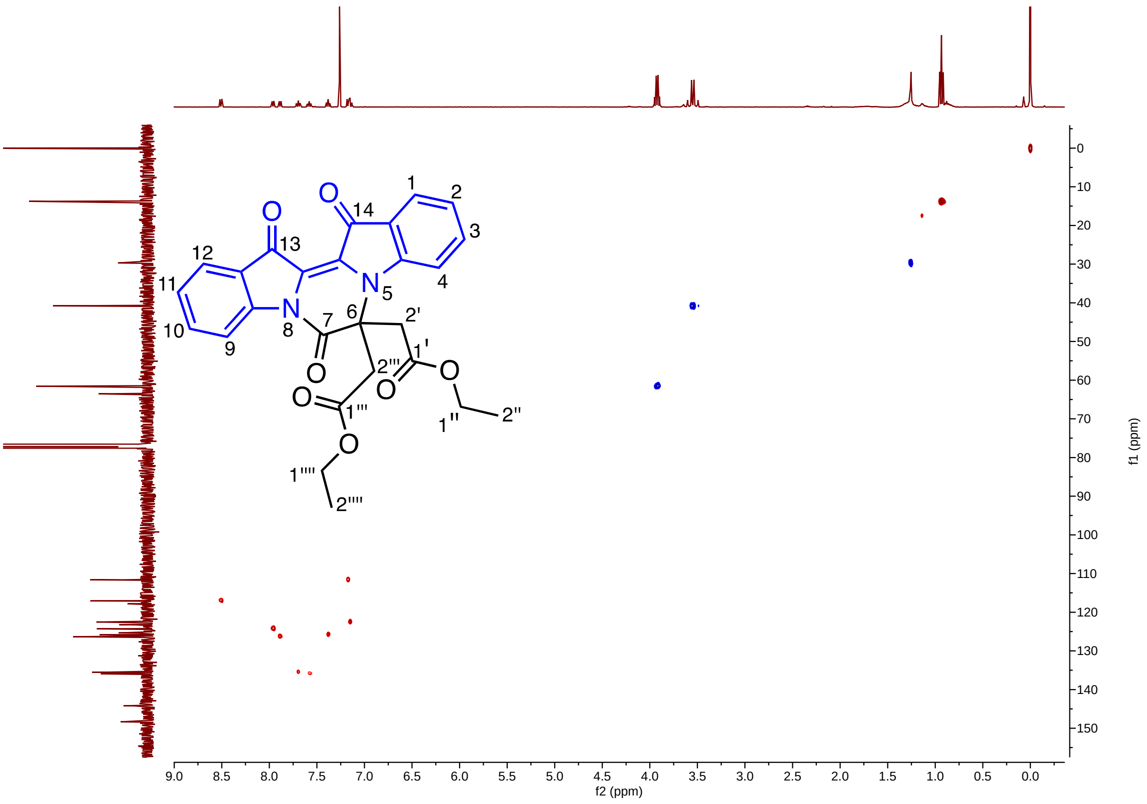

Figure S37: HSQC spectrum of the compound **23** recorded in $CDCl_3$ using 400 MHz NMR instrument.

NMR spectra of compound 25.

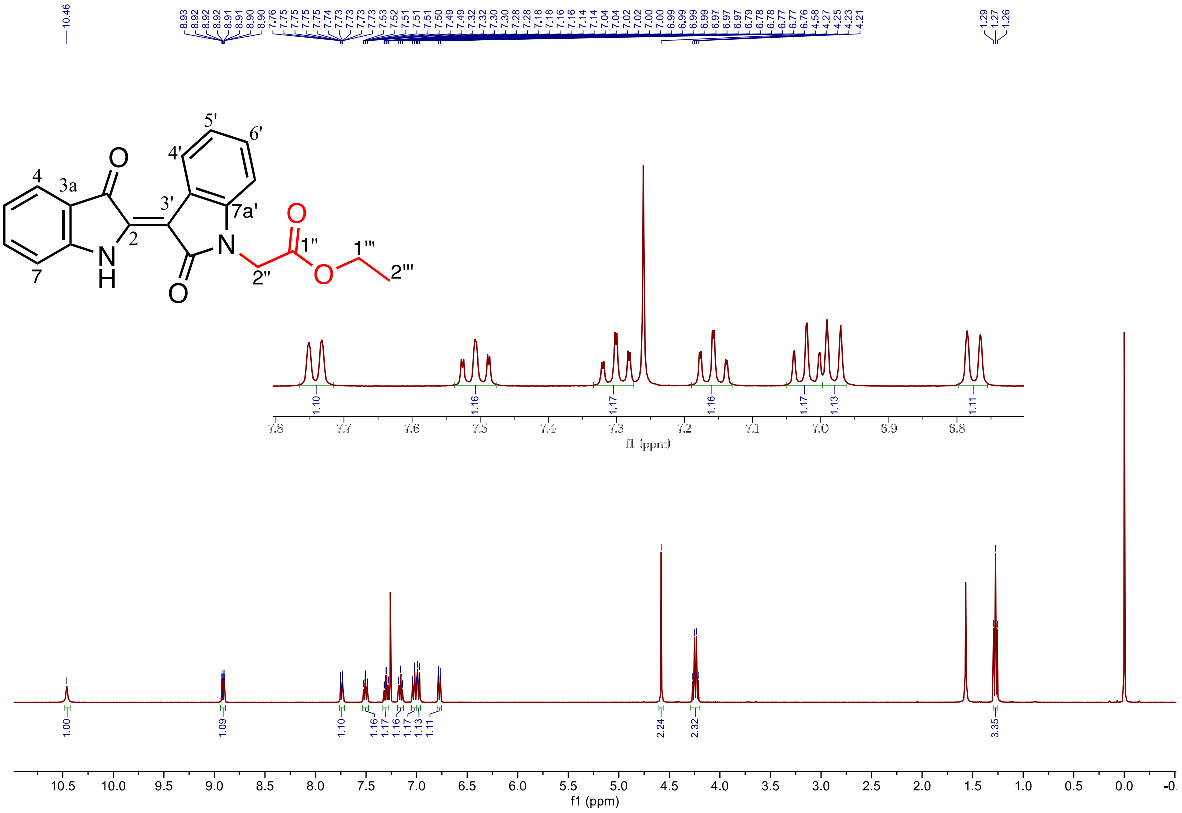

Figure S38: ^{1}H NMR spectrum of the compound **25** ($CDCl_3$, 400 MHz).

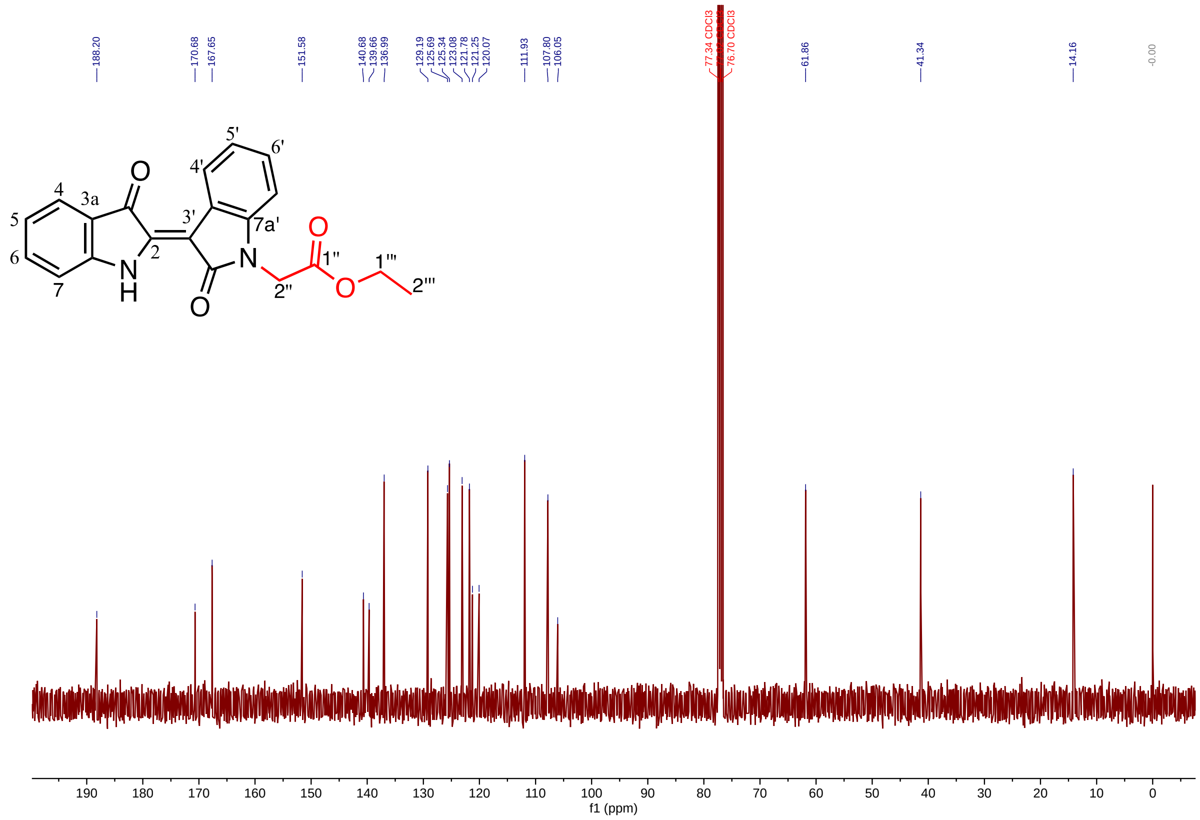

Figure S39: ^{13}C NMR spectrum of the compound **25** recorded in $CDCl_3$ using 100MHz NMR instrument.

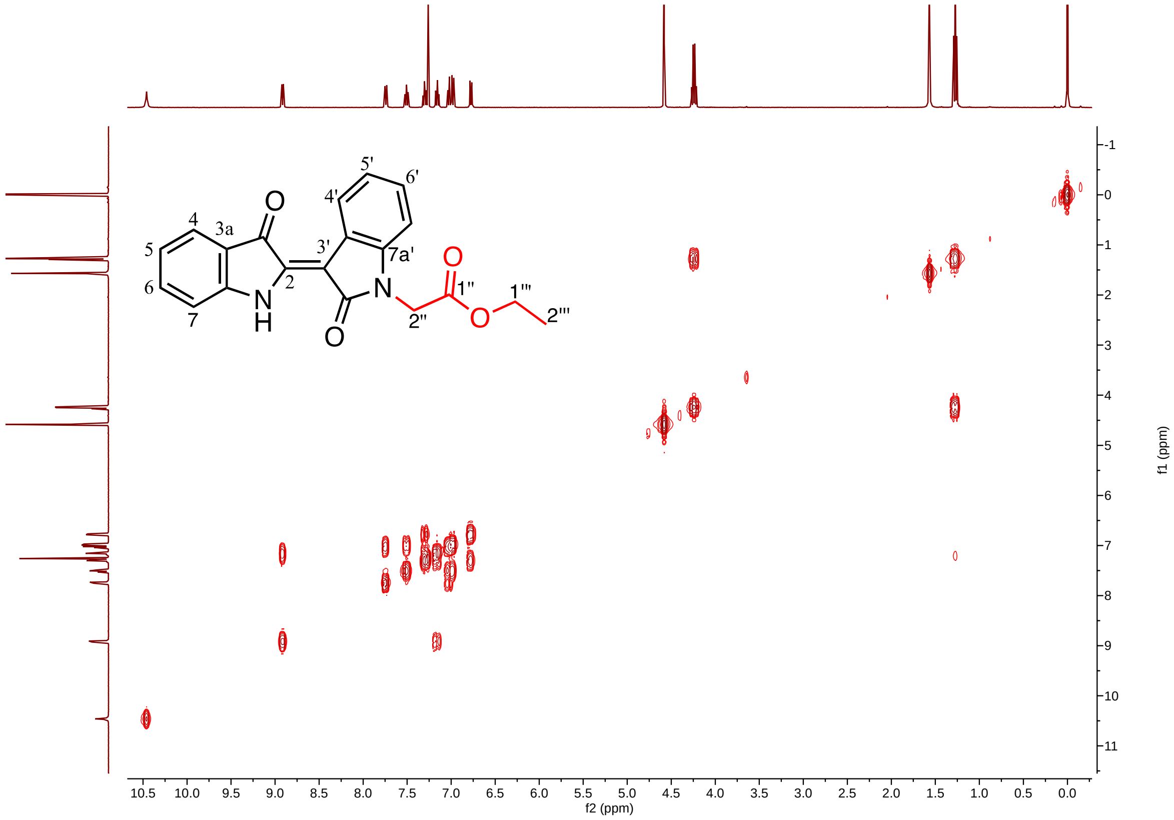

Figure S40: COSY spectrum of the compound **25** ($CDCl_3$, 400 MHz).

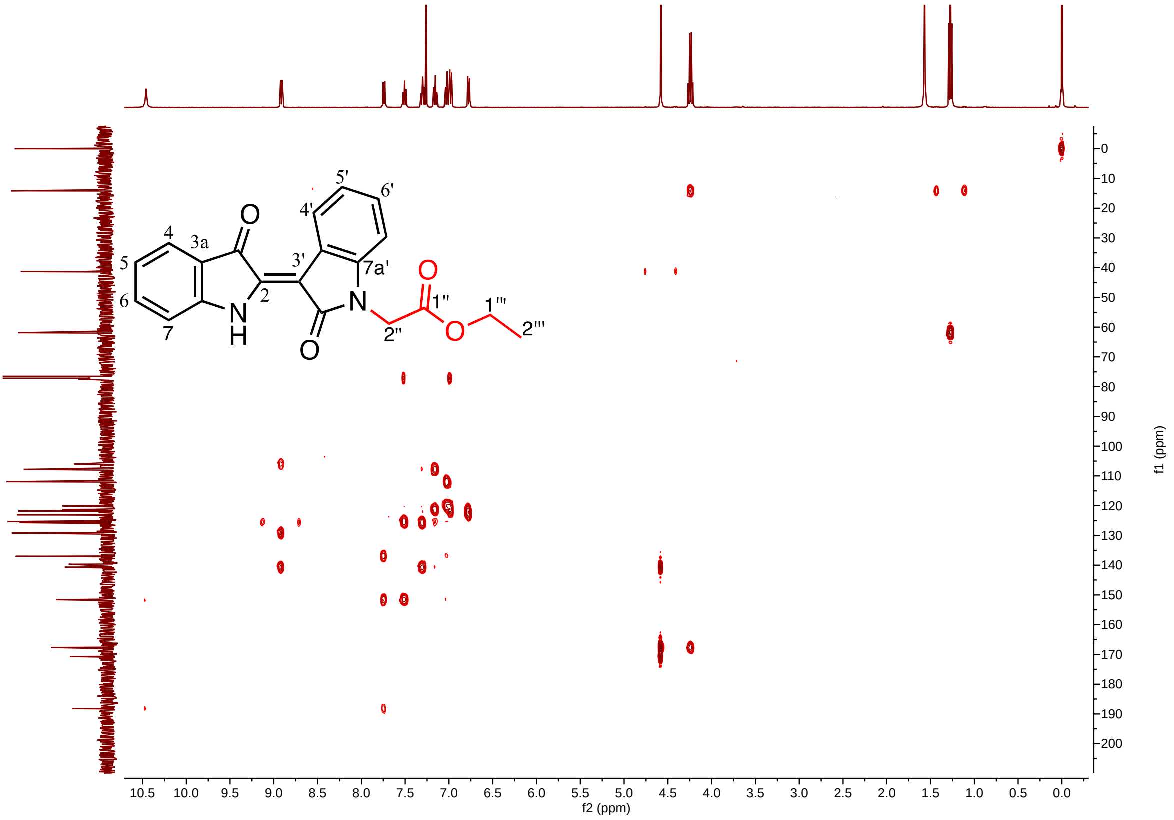

Figure S41: HMBC spectrum of the compound **25** recorded in $CDCl_3$ using 400 MHz NMR instrument.

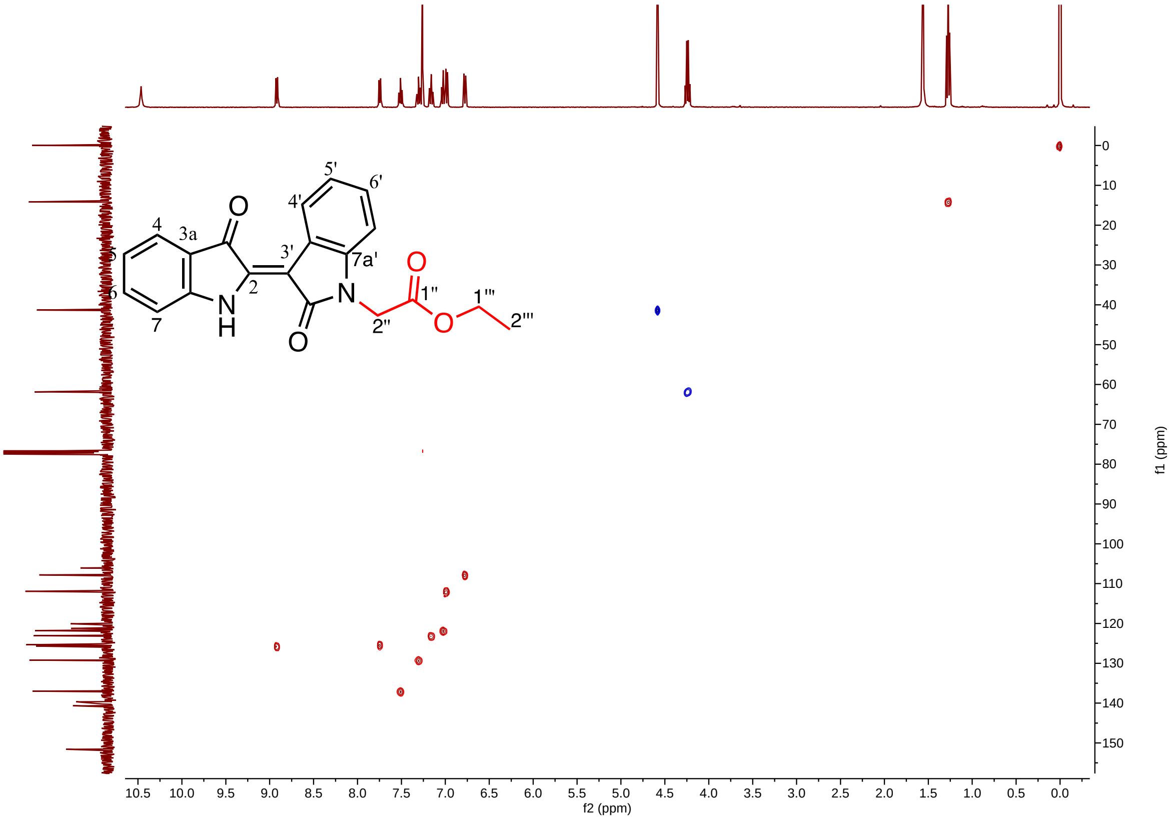

Figure S42: HSQC spectrum of the compound **25** recorded in $CDCl_3$ using 400 MHz NMR instrument.

NMR spectra of compound 26.

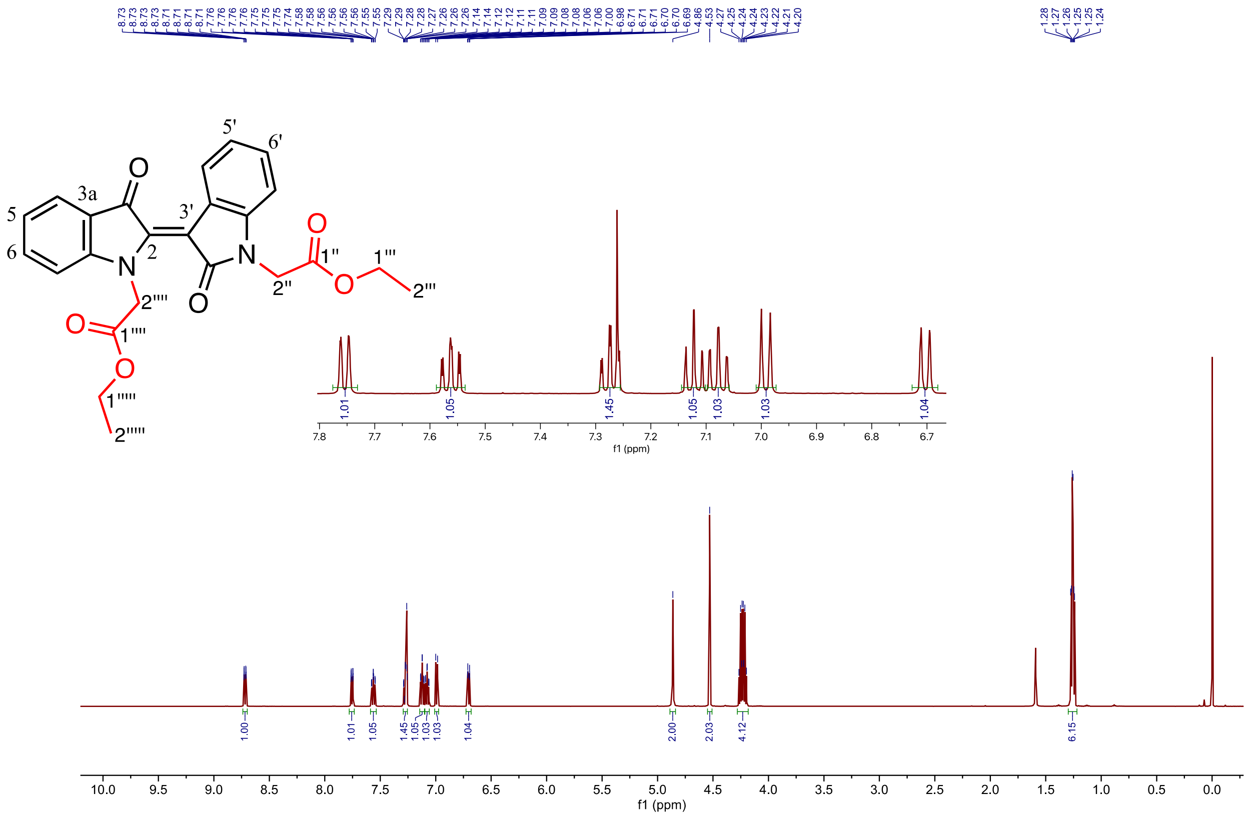

Figure S43: ^{1}H NMR spectrum of the compound **26** ($CDCl_3$, 400 MHz).

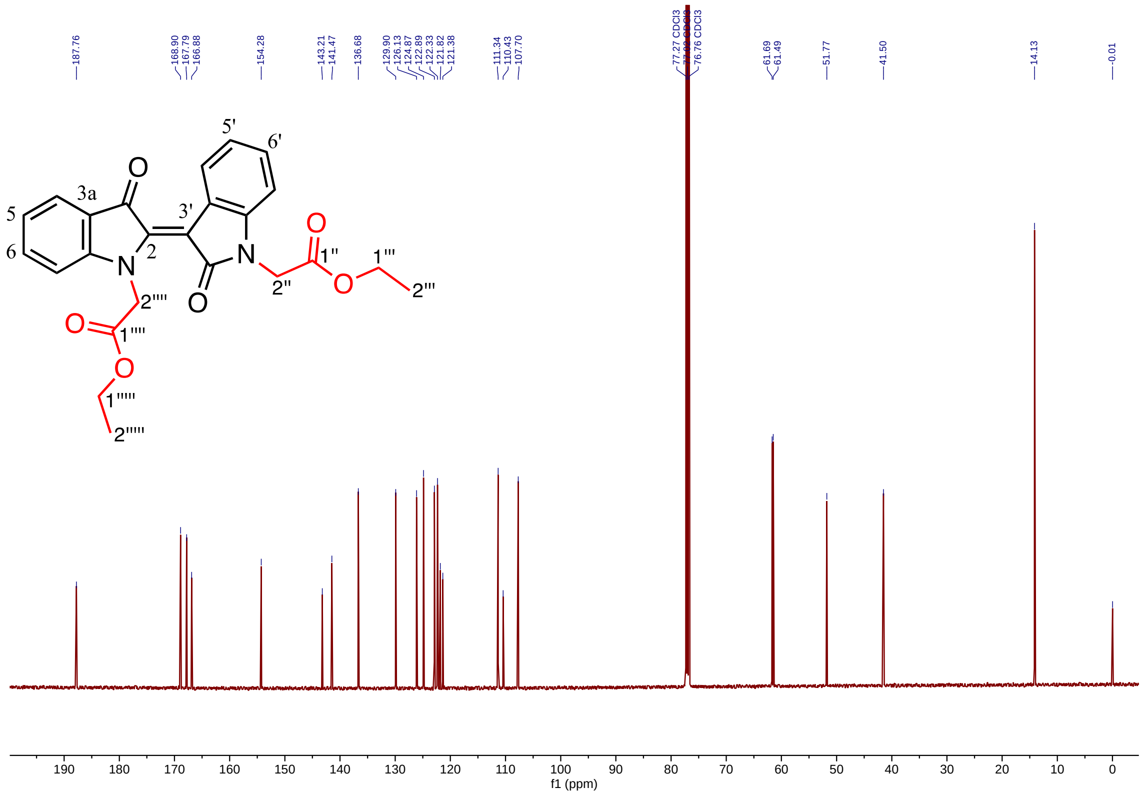

Figure S44: ^{13}C NMR spectrum of the compound **26** recorded in $CDCl_3$ using 125 MHz NMR instrument.

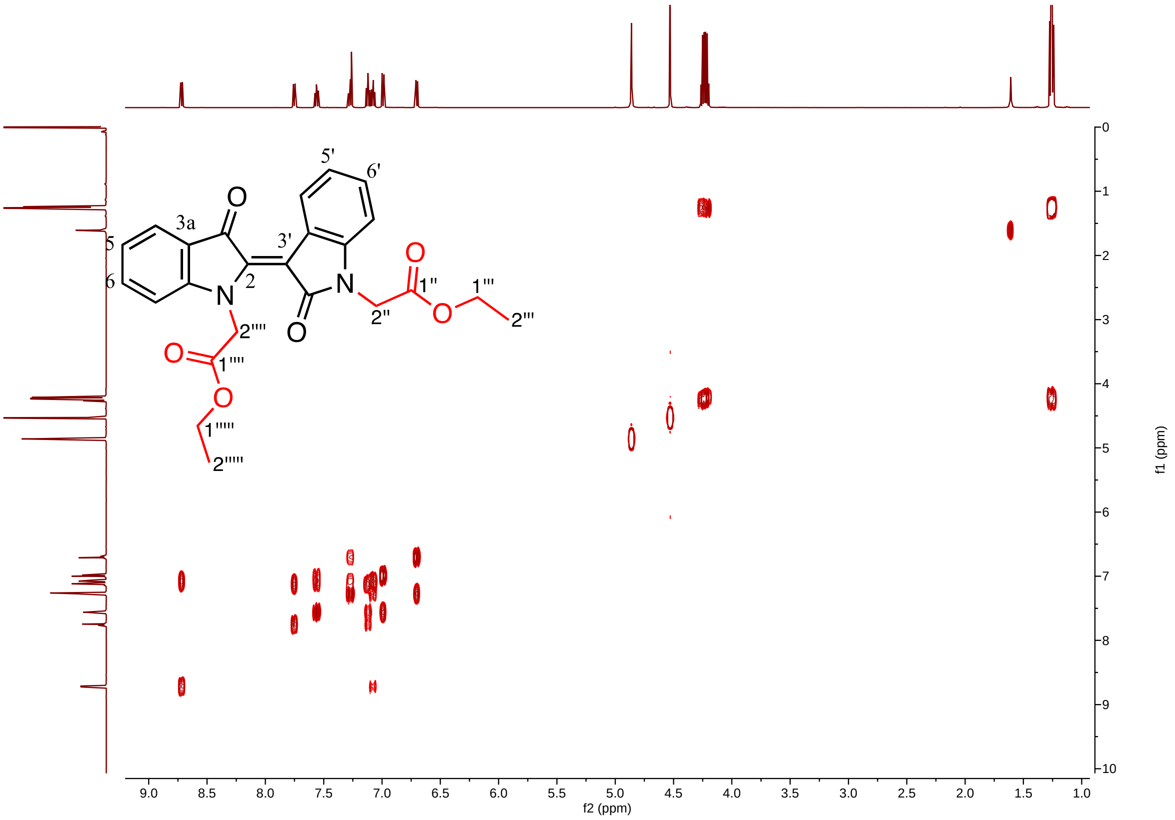

Figure S45: COSY spectrum of the compound **26** ($CDCl_3$, 500 MHz).

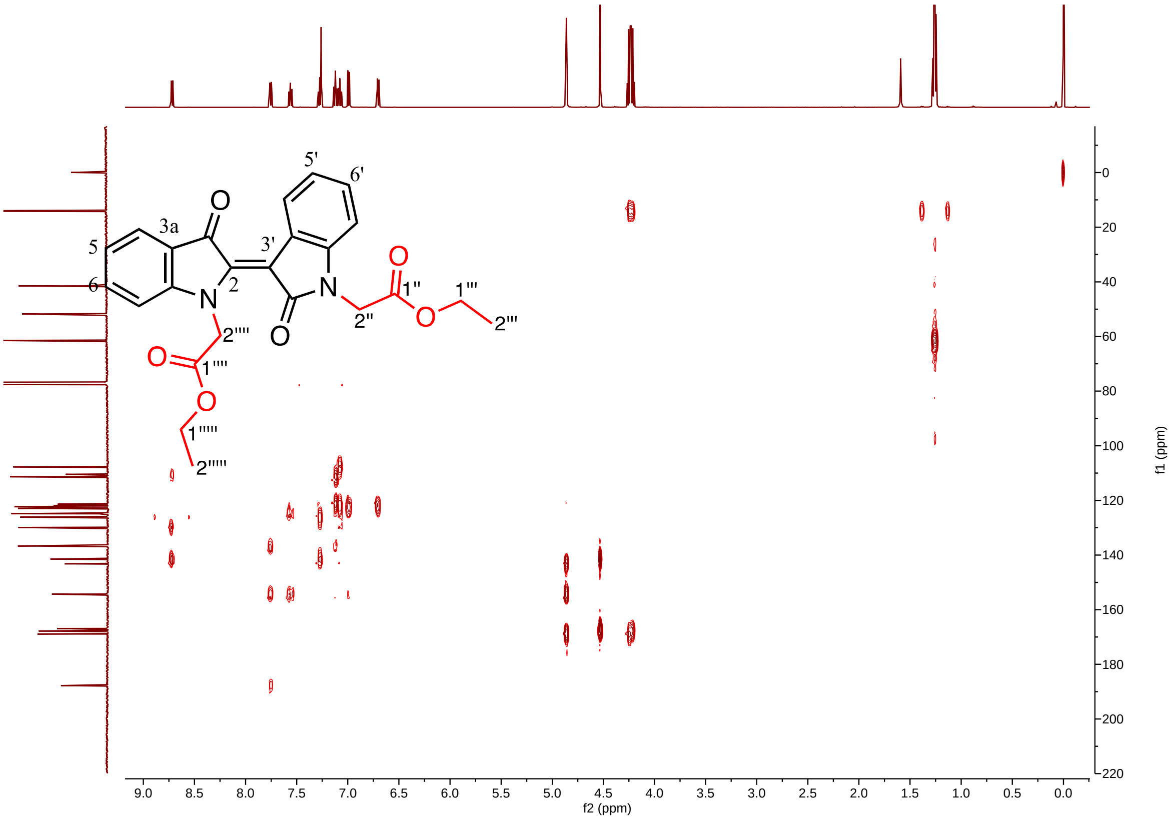

Figure S46: HMBC spectrum of the compound **26** recorded in $CDCl_3$ using 500 MHz NMR instrument.

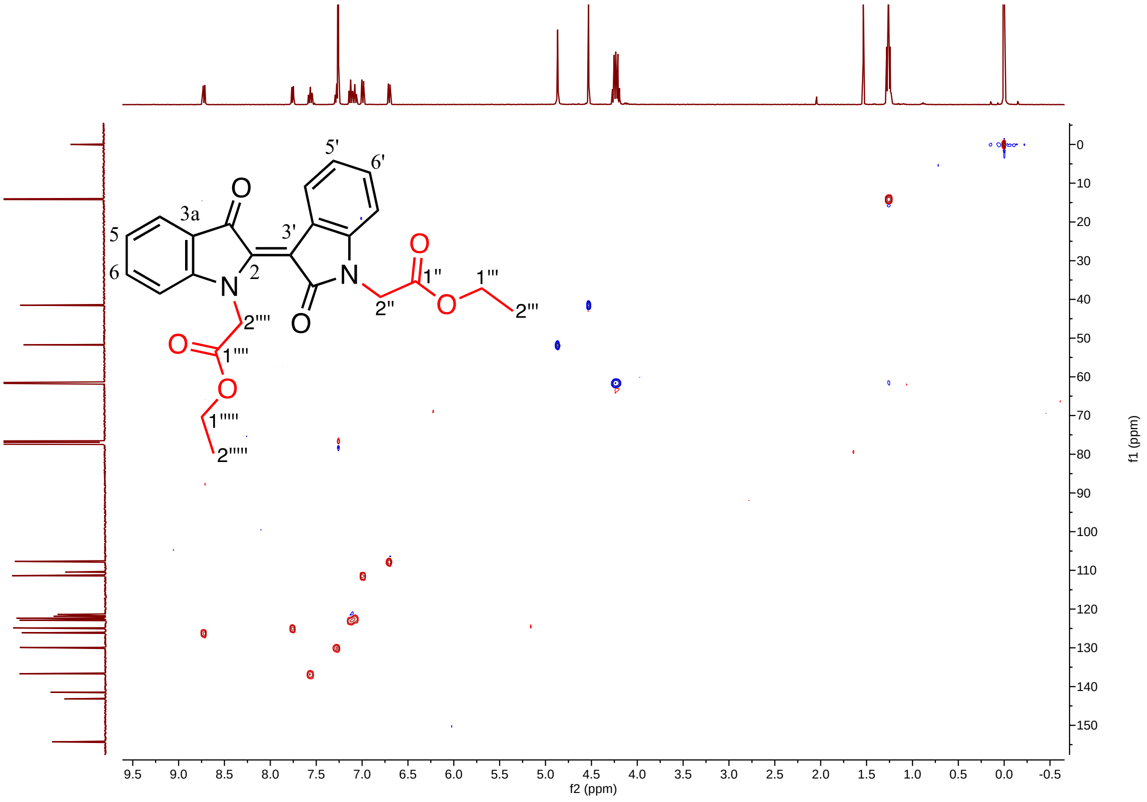

Figure S47: HSQC spectrum of the compound **26** recorded in $CDCl_3$ using 400 MHz NMR instrument.

NMR spectra of compound 27.

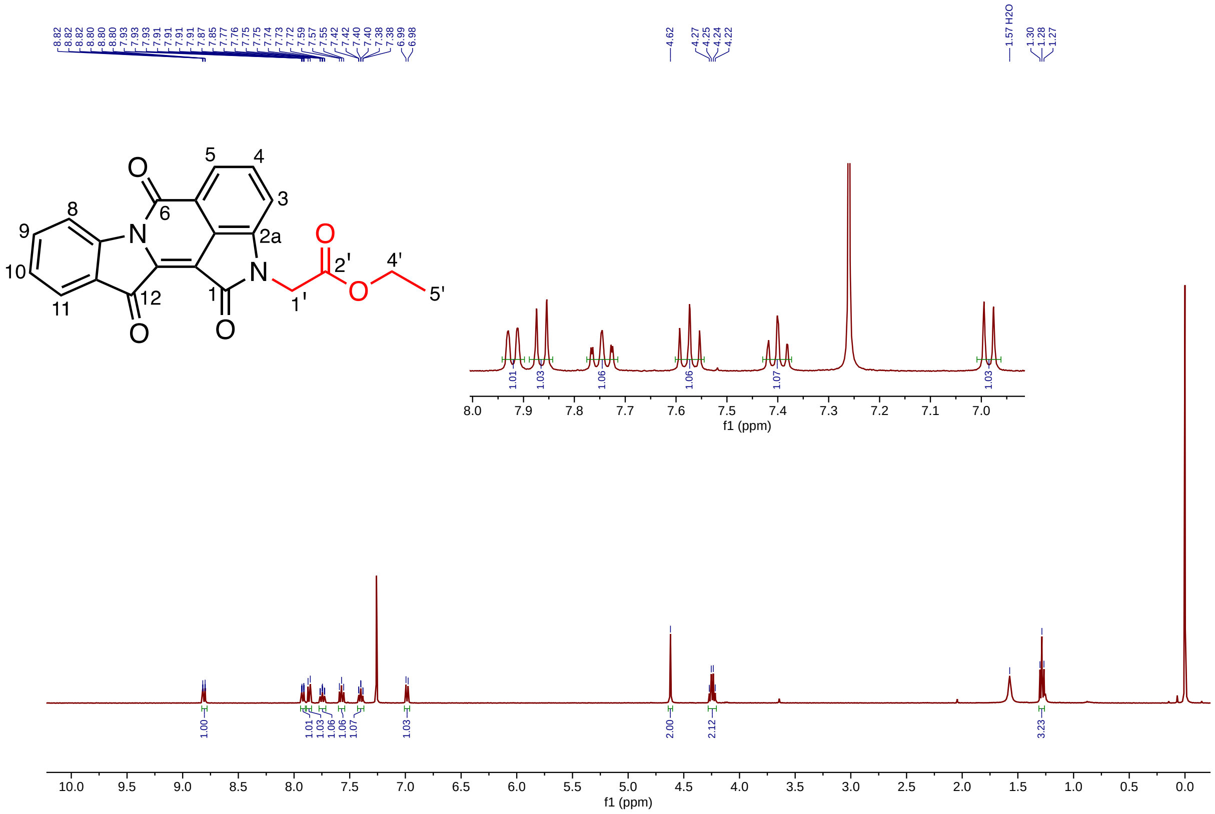

Figure S48: ^{1}H NMR spectrum of the compound **27** ($CDCl_3$, 400 MHz).

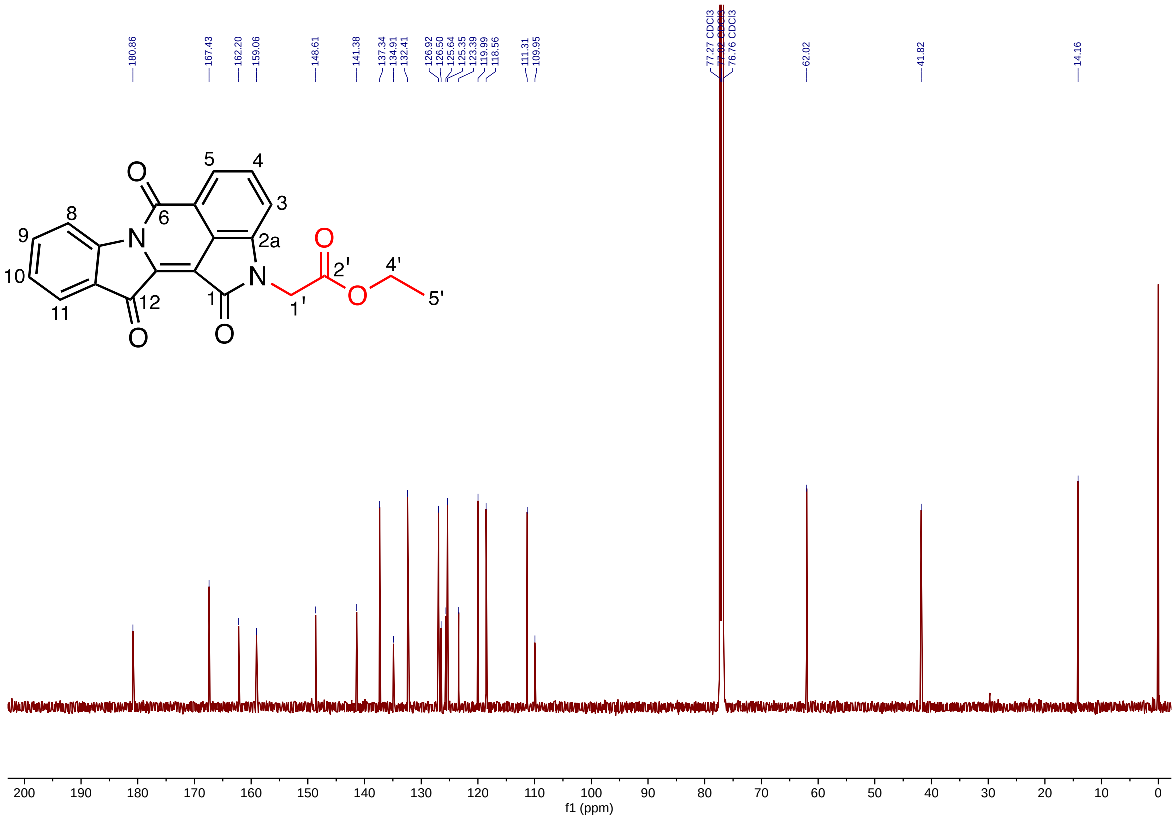

Figure S49: ^{13}C NMR spectrum of the compound **27** recorded in $CDCl_3$ using 125 MHz NMR instrument.

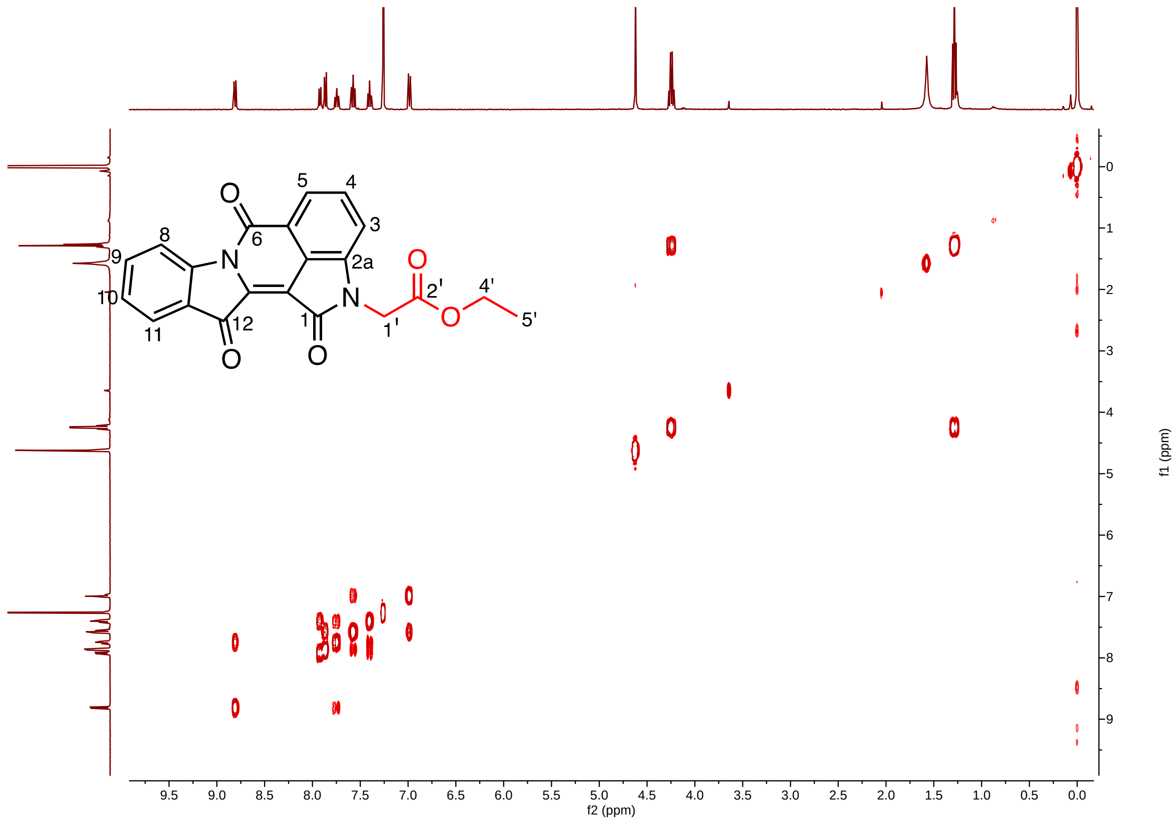

Figure S50: COSY spectrum of the compound **27** ($CDCl_3$, 400 MHz).

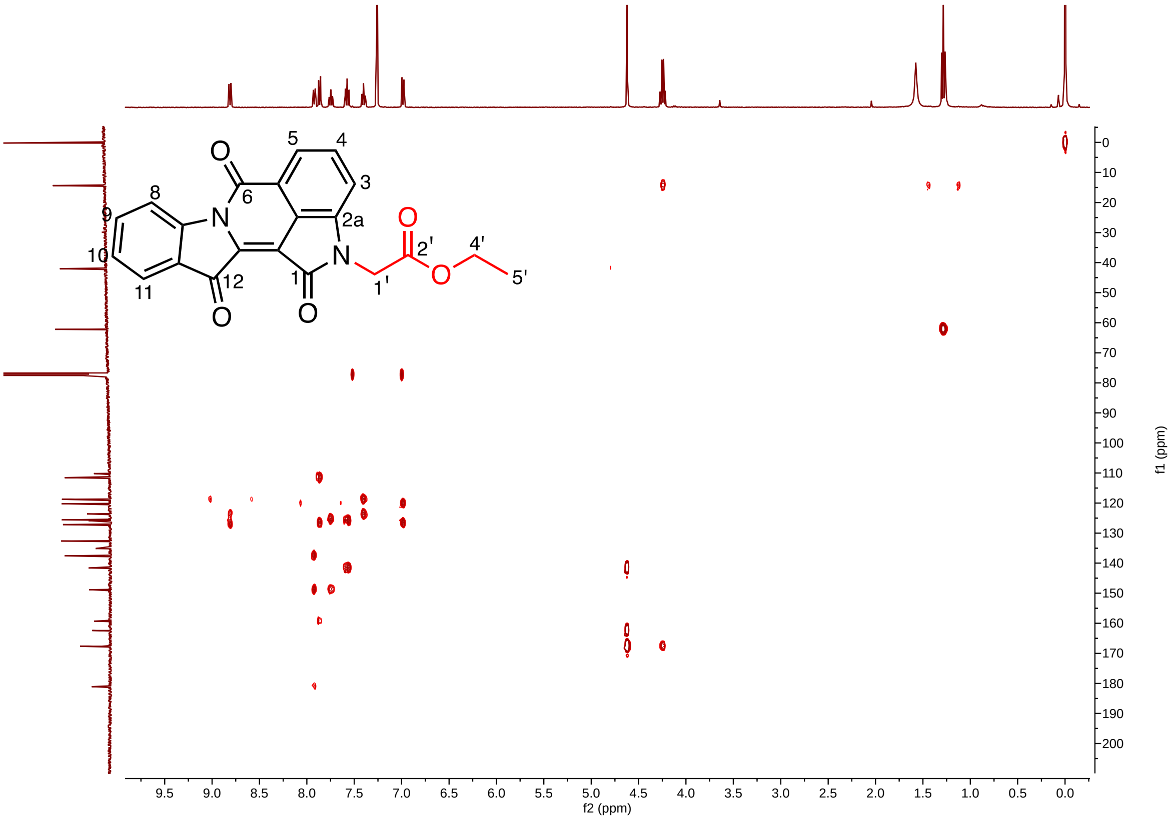

Figure S51: HMBC spectrum of the compound **27** recorded in $CDCl_3$ using 400 MHz NMR instrument.

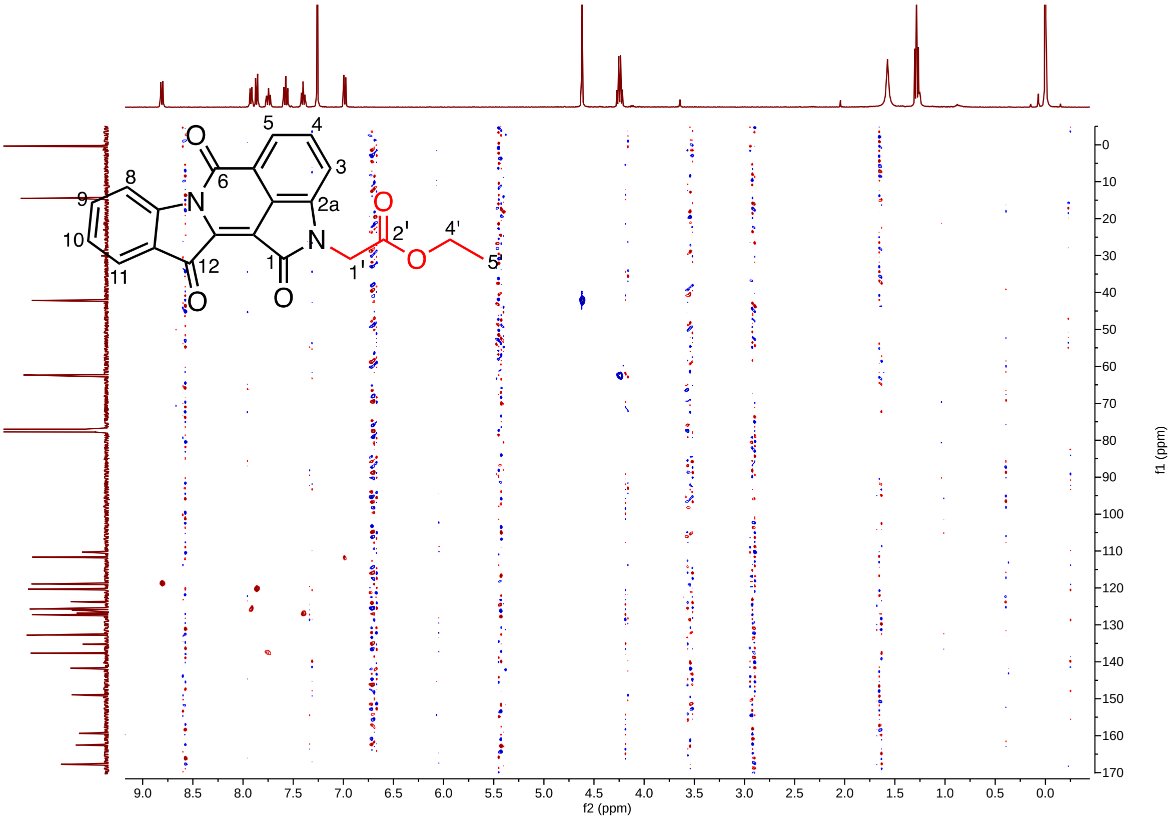

Figure S52: HSQC spectrum of the compound **27** recorded in $CDCl_3$ using 500 MHz NMR instrument.

NMR spectra of compound 28.

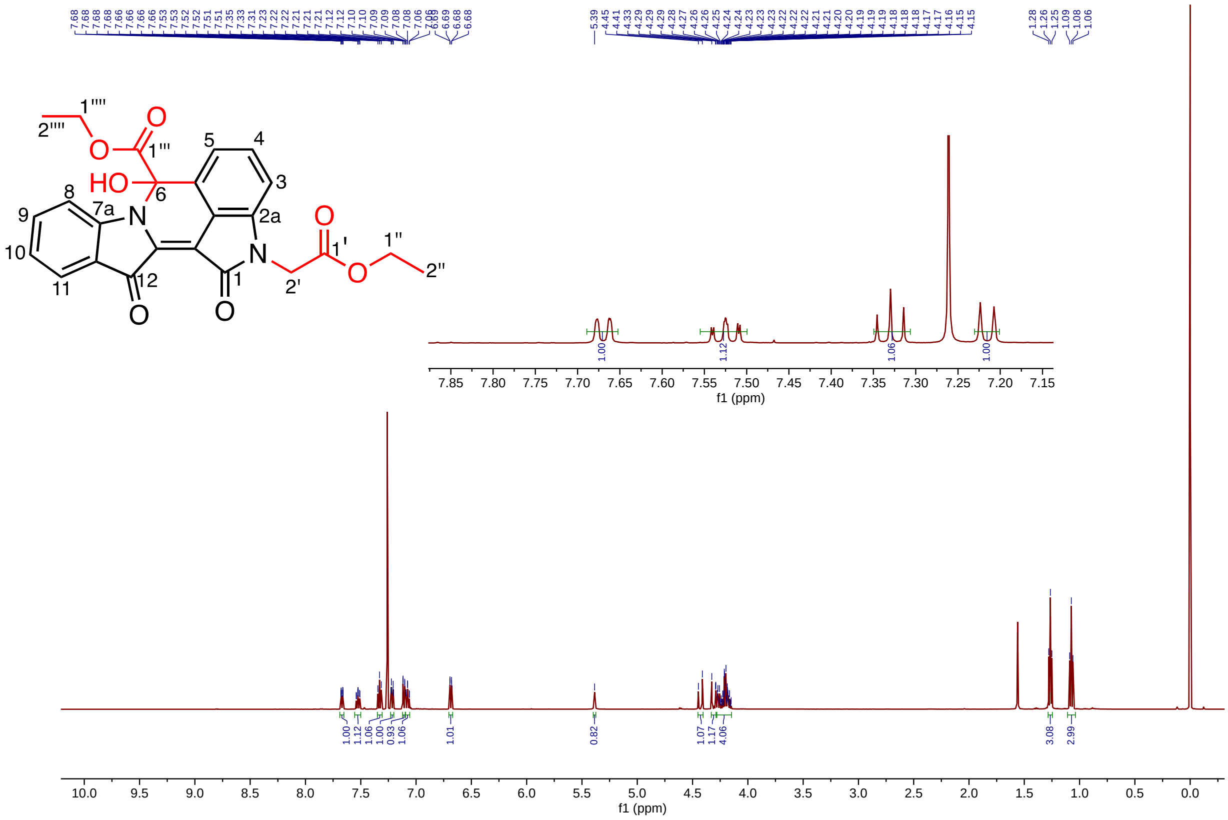

Figure S53: ^{1}H NMR spectrum of the compound **28** ($CDCl_3$, 500 MHz).

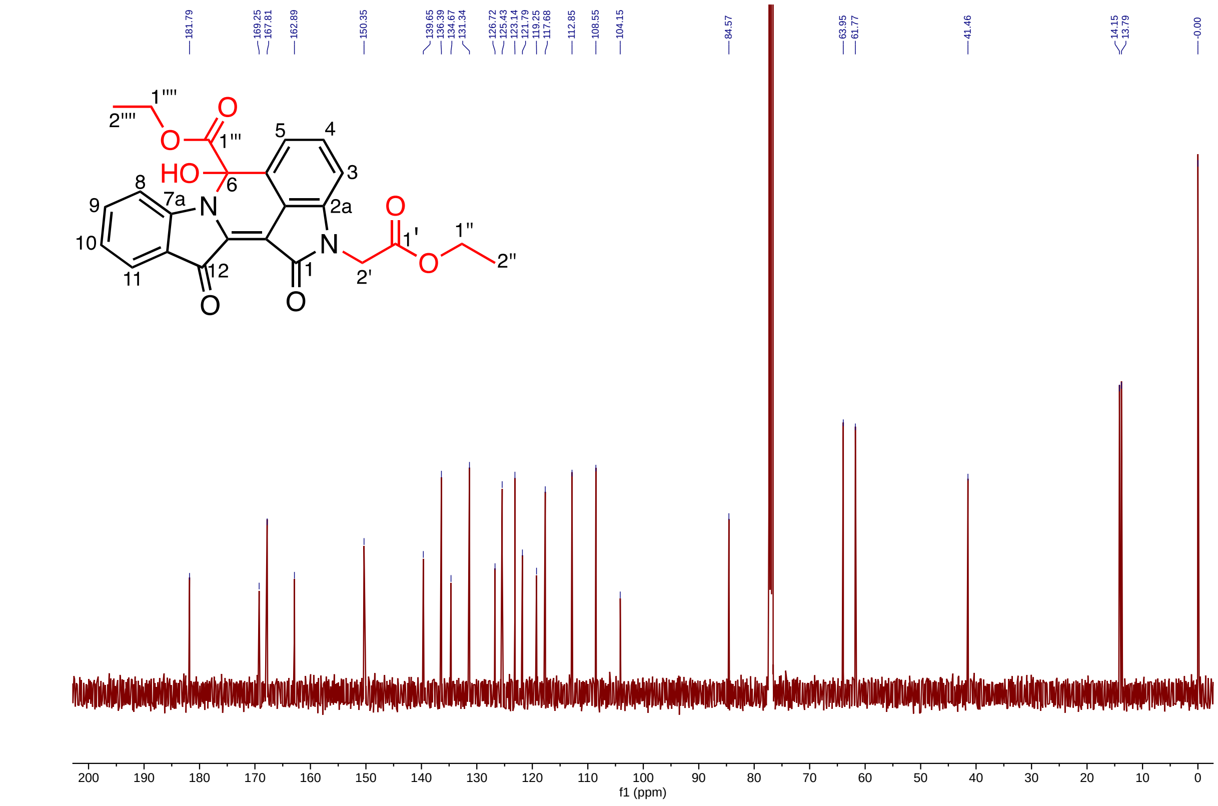

Figure S54: ^{13}C NMR spectrum of the compound **28** recorded in $CDCl_3$ using 125 MHz NMR instrument.

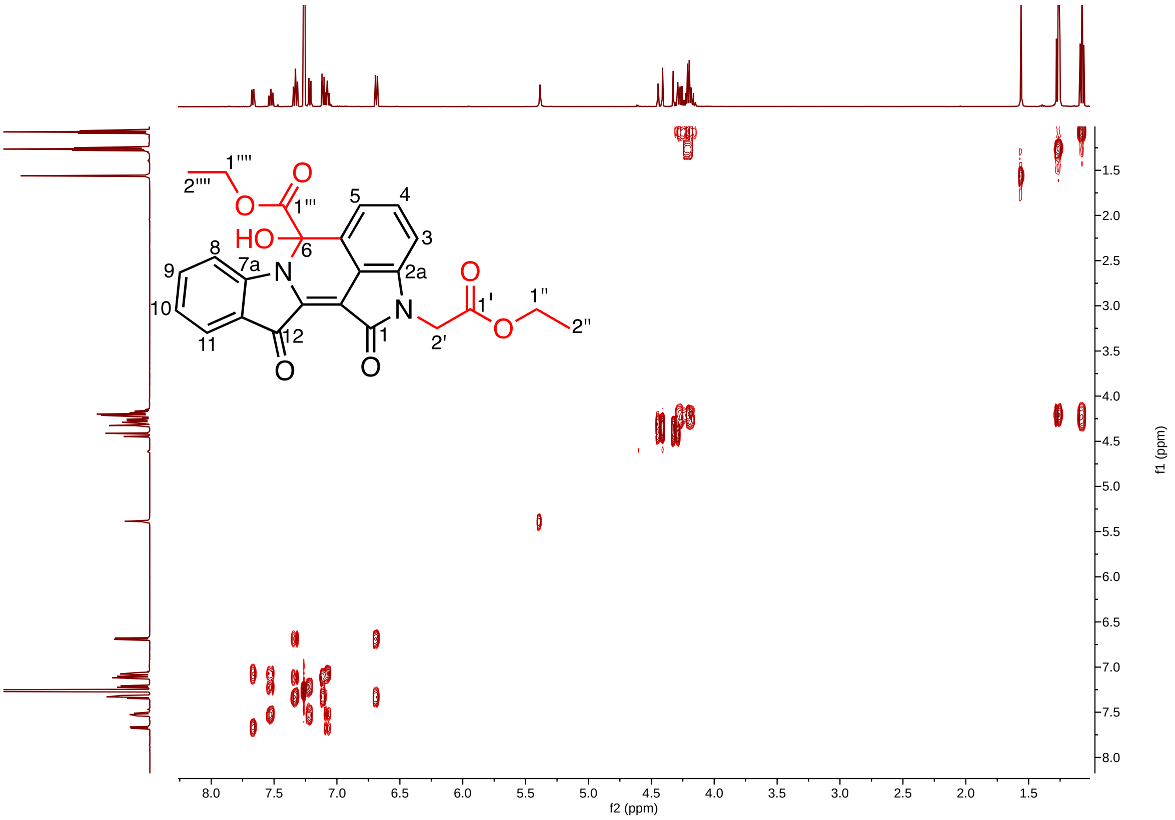

Figure S55: COSY spectrum of the compound **28** ($CDCl_3$, 500 MHz).

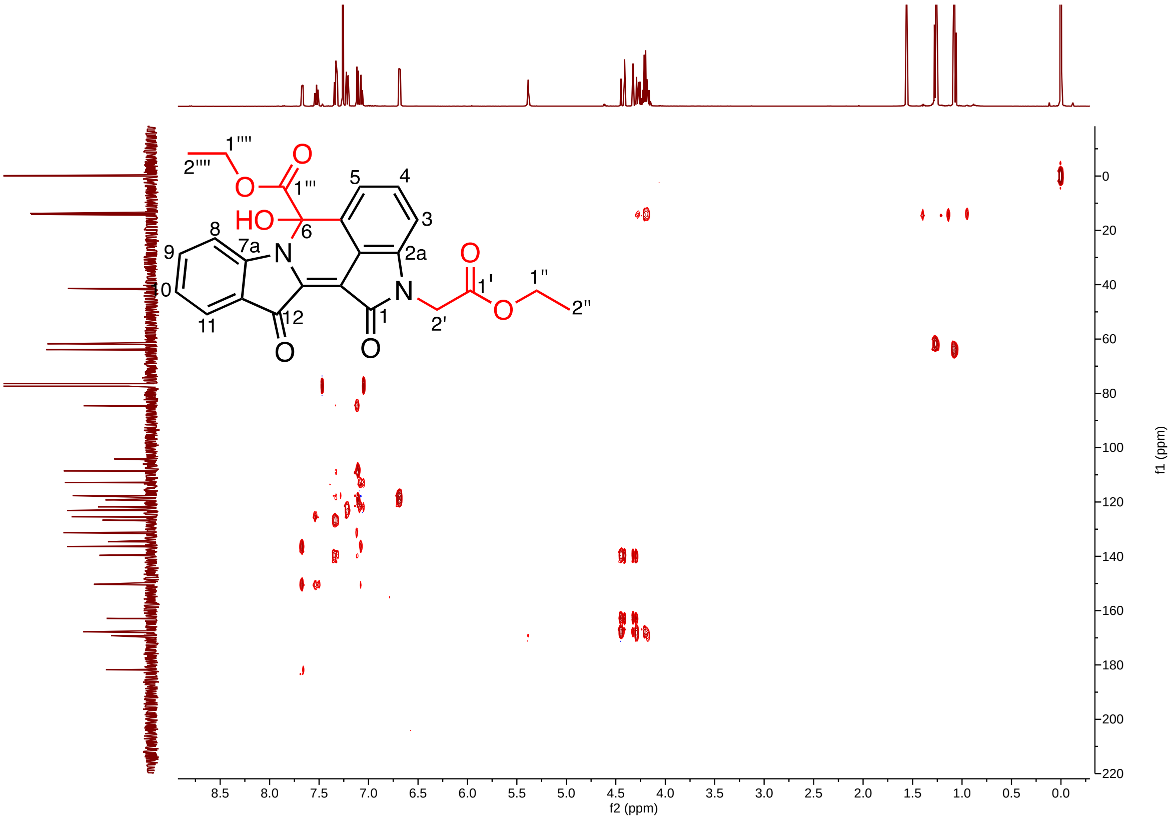

Figure S56: HMBC spectrum of the compound **28** recorded in $CDCl_3$ using 500 MHz NMR instrument.

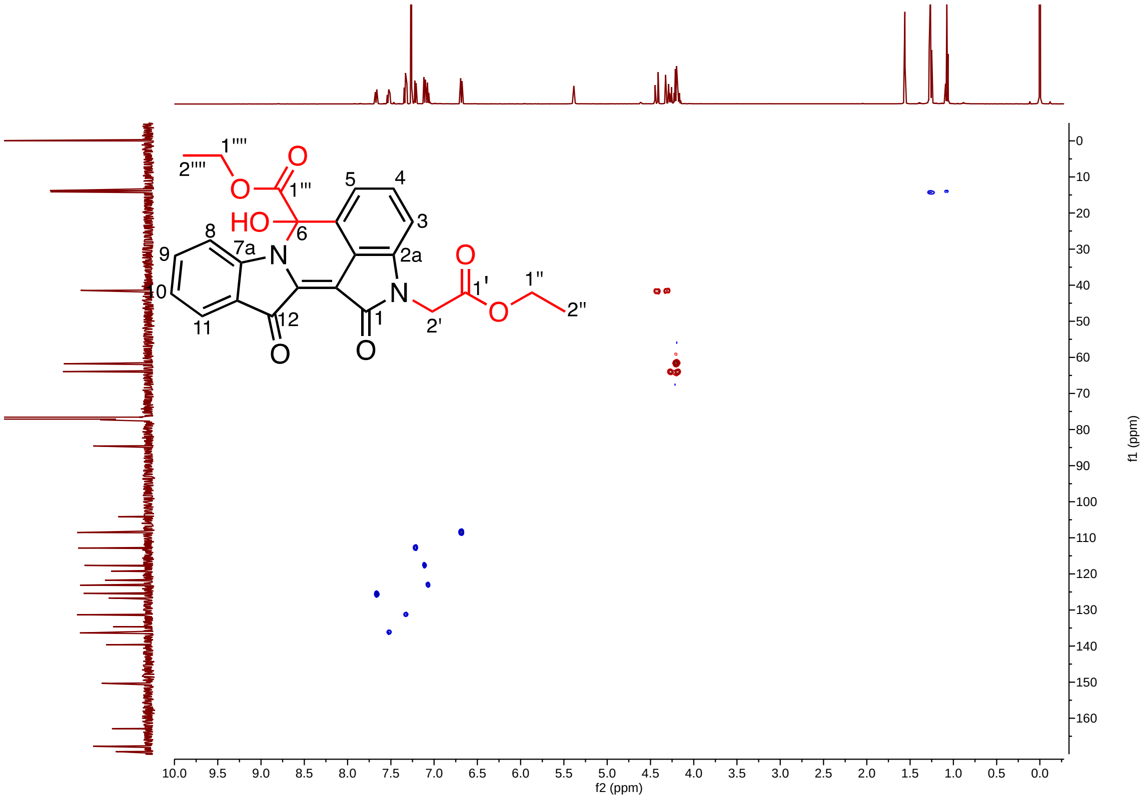

Figure S57: HSQC spectrum of the compound **28** recorded in $CDCl_3$ using 400 MHz NMR instrument.

NMR spectra of compound 29.

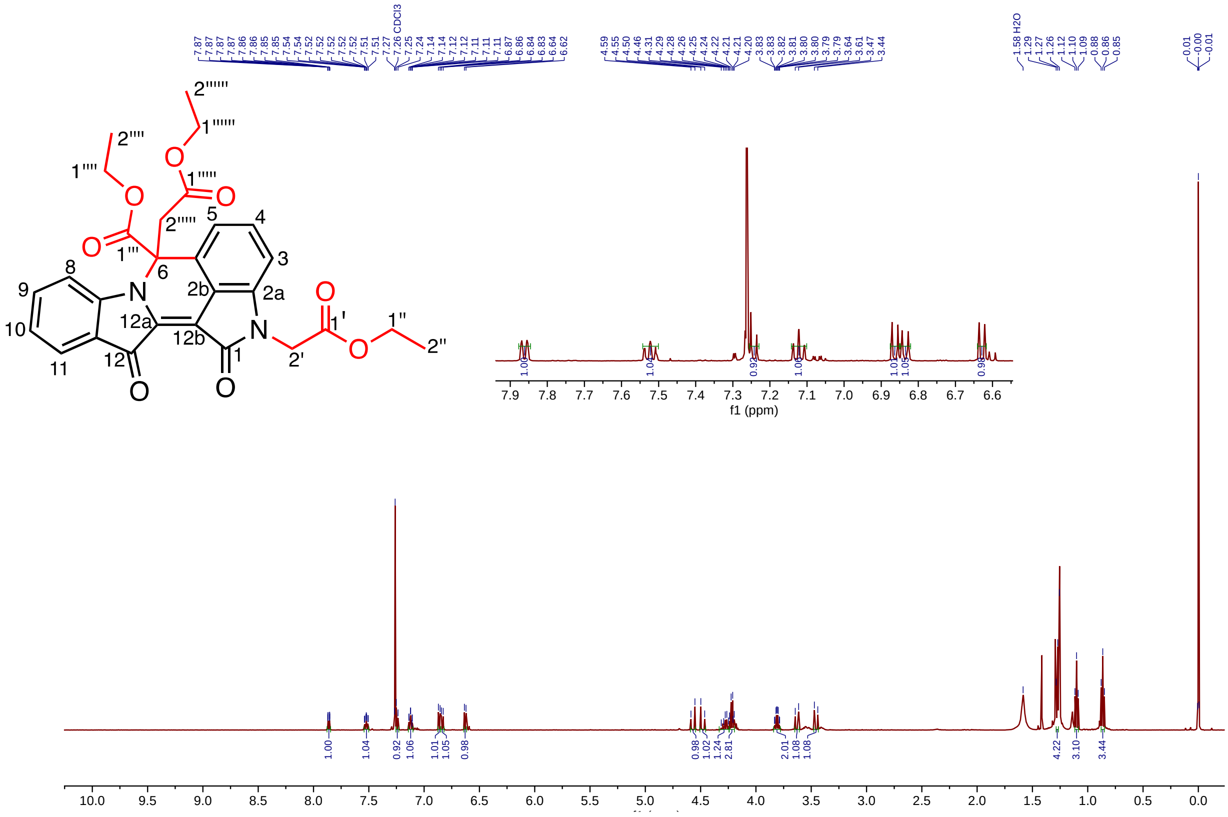

Figure S58: ^{1}H NMR spectrum of the compound **29** ($CDCl_3$, 500 MHz).

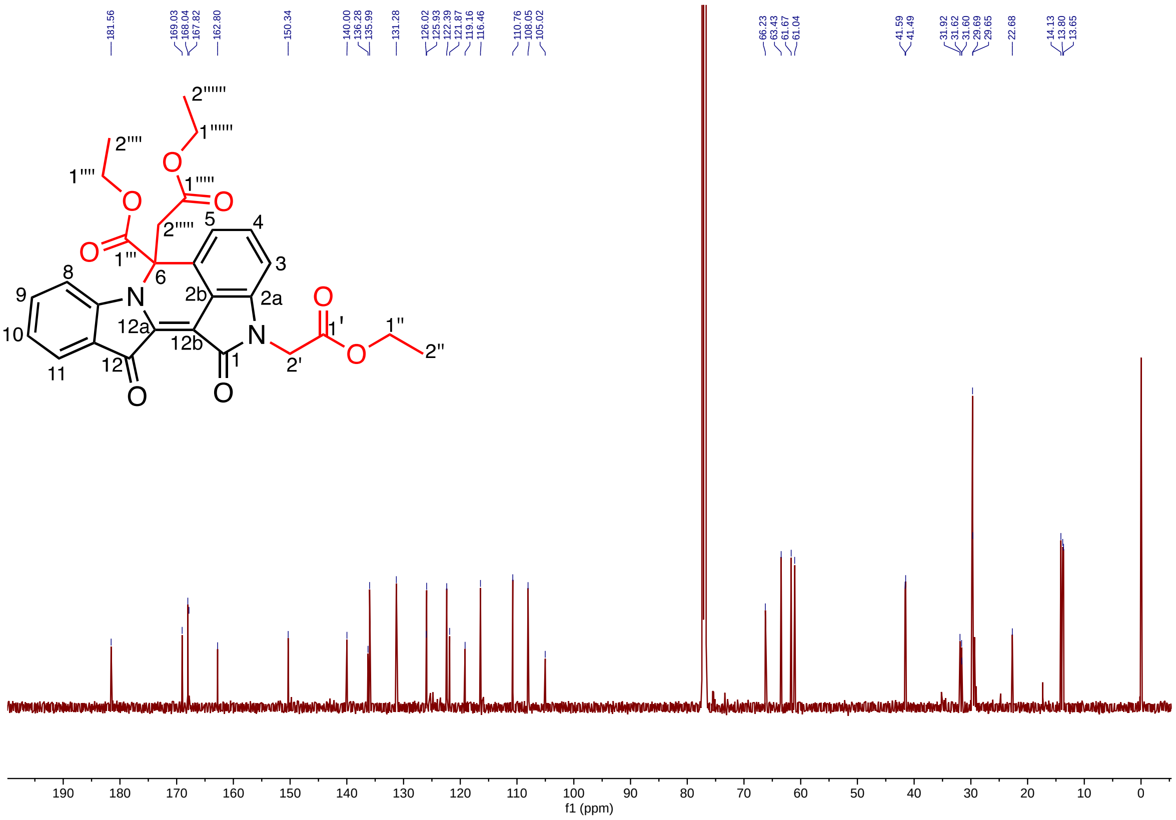

Figure S59: ^{13}C NMR spectrum of the compound **29** recorded in $CDCl_3$ using 125 MHz NMR instrument.

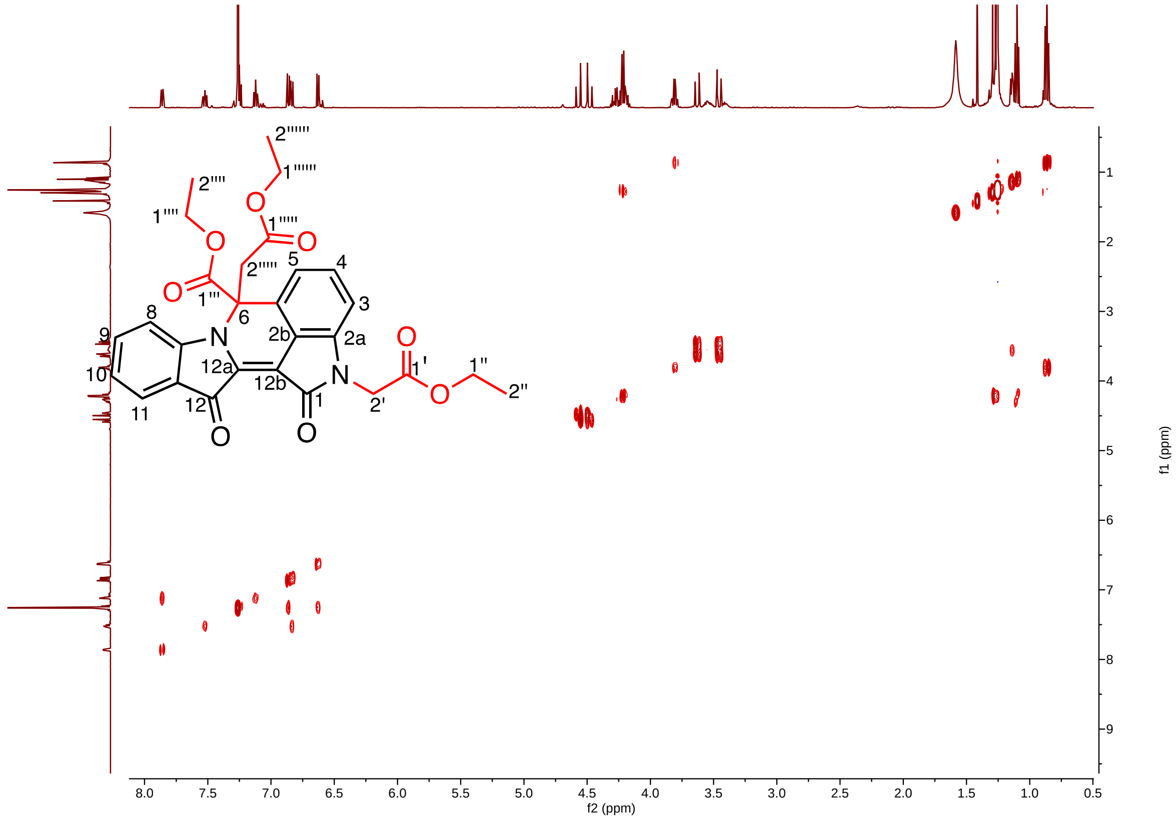

Figure S60: COSY spectrum of the compound **29** ($CDCl_3$, 500 MHz).

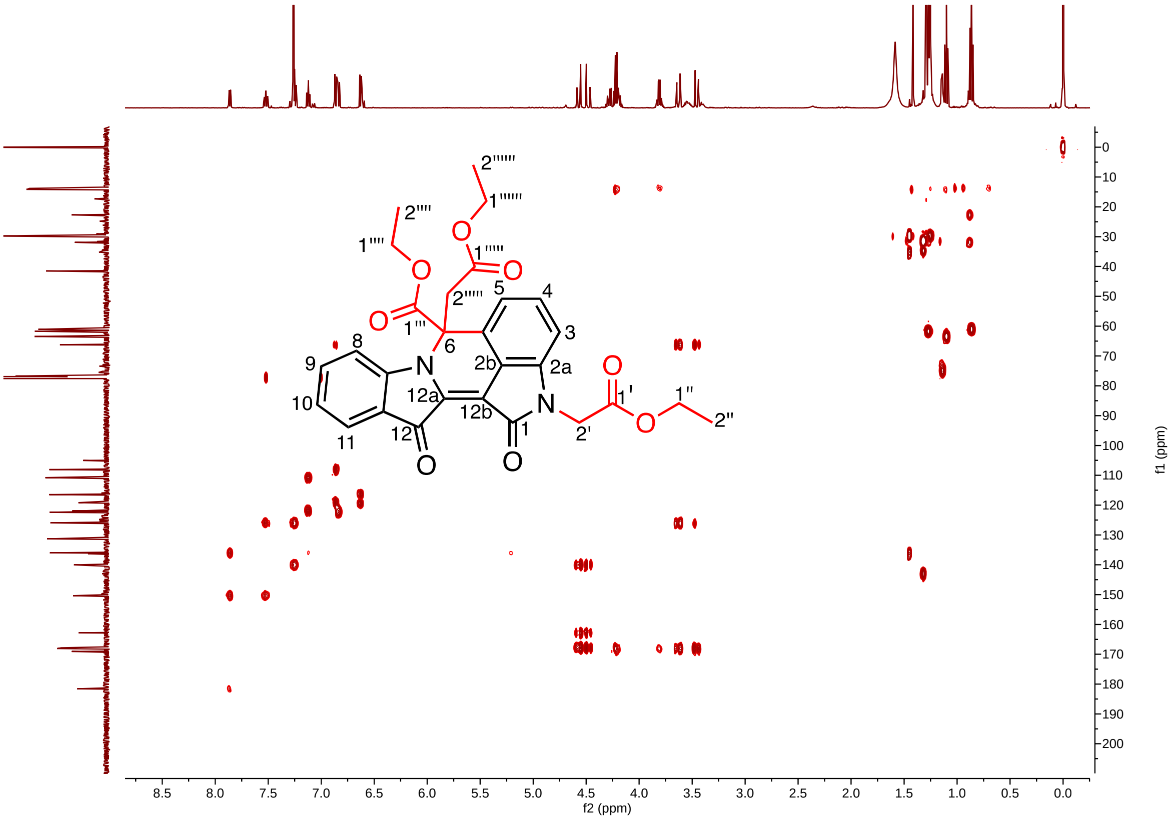

Figure S61: HMBC spectrum of the compound **29** recorded in $CDCl_3$ using 400 MHz NMR instrument.

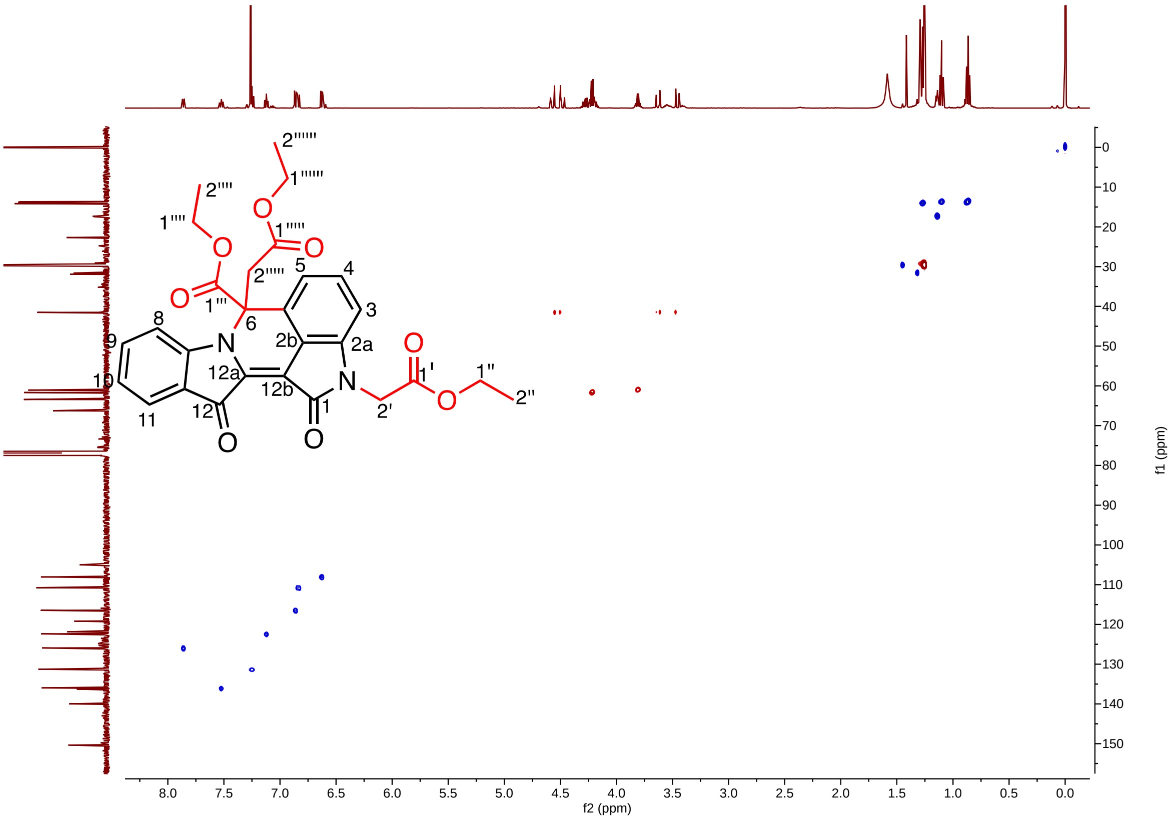

Figure S62: HSQC spectrum of the compound **29** recorded in $CDCl_3$ using 400 MHz NMR instrument.

X-ray Crystallography Data

Structure determination

Images were measured on an Agilent SuperNova diffractometer (Cu $K\alpha$ radiation, mirror monochromator, λ= 1.54184 Å) and data were extracted using the CrysAlis PRO package. Structure solution was by direct methods (SIR92). The structures were refined using the CRYSTALS program package. Note the numbering used in the X-ray ORTEP diagrams is different from the IUPAC systematic numbering of the structures.

Compound 18

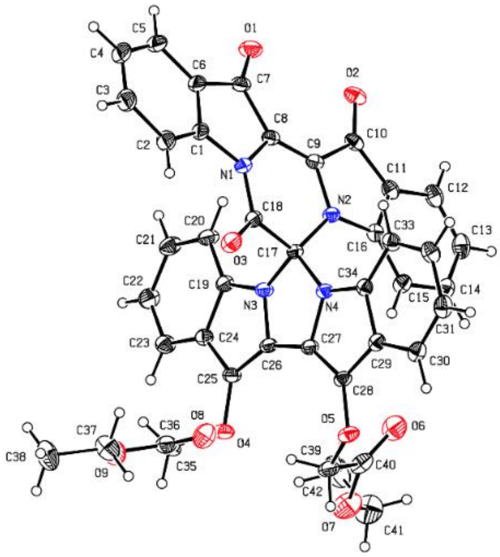

Figure S63: $C_{42}H_{30}N_4O_9$. M_r = 734.72, T = 150 K, monoclinic, space group $P2_1/n$ Z = 4, a = 15.4563 (2) Å, b = 10.2073 (1) Å, c = 22.5804 (4) Å, β = 105.8678 (16)°, V = 3426.70 (9) Å^3, D_x = 1.424 Mg m^{-3}, Cu $K\alpha$ radiation, λ = 1.54184 Å, 54601 reflections measured (Θ_{range} = 4.0-73.0°), merged to 6930 unique data, R = 0.052 [for 5800 data points with $I > 2.0\sigma(I)$], R_w = 0.141 [all data], S = 0.99. There was disorder in the packing of both –$C_4H_7O_2$ sidechains corresponding to different conformations of the chains: atoms C37 and C38 each have three sites, C41 and C42 each have two, Distances, angles and displacement parameters for these sites were restrained during refinement, and the occupancies were refined appropriately. Anisotropic displacement ellipsoids display 30% probability levels.
CCDC 2371741.

Compound 17

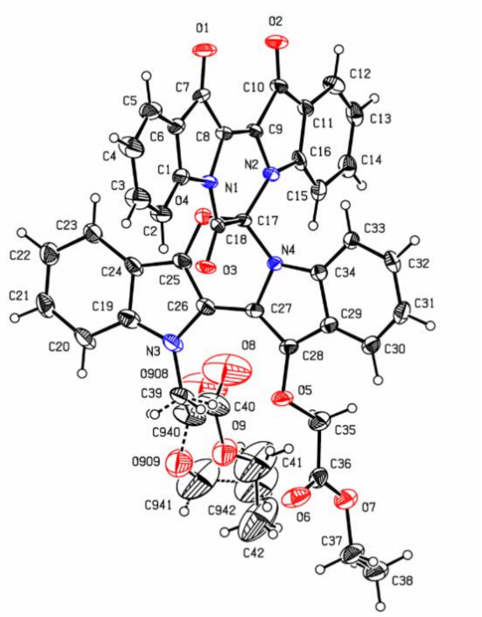

Figure S64: $C_{42}H_{30}N_4O_9$. $0.50CH_2Cl_2$.$0.282H_2O$ (solvent omitted from molecular illustration). M_r = 781.67, T =150 K, Monoclinic, space group $P2_1/c$, Z = 4, a = 15.1688 (17) Å, b = 10.0980 (9) Å, c = 24.637 (2) Å, β = 100.545 (10)°, V = 3710.0 (6) Å^3, D_x = 1.395 Mg m^{-3}, Mo $K\alpha$ radiation, λ = 0.71073 Å, 34640 reflections measured (Θ_{range} = 3.4-25.0°), merged to 6524 unique data, R = 0.107 [for 4116 data points with $I > 2.0\sigma(I)$], R_w = 0.324 [all data], S = 0.97. CCDC No. 2374005. Anisotropic displacement ellipsoids display 30% probability levels.

Compound 20

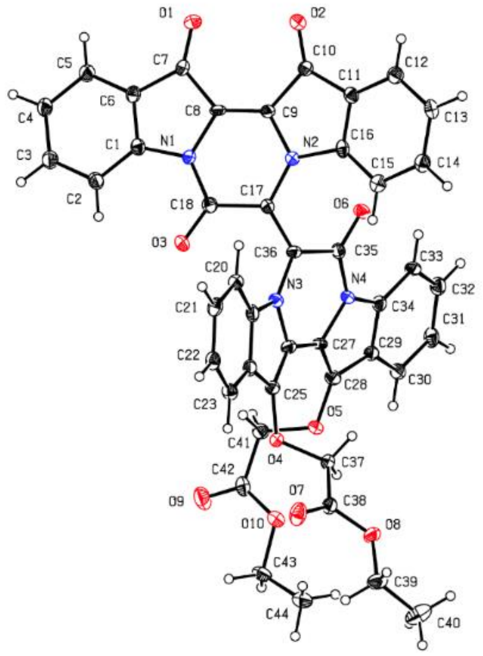

Figure S65: $C_{44}H_{30}N_4O_{10}$. $0.470CH_2Cl_2$.$0.282H_2O$ (solvent omitted from molecular illustration). M_r = 774.74, T = 150 K, monoclinic, space group $P2_1/c$, Z = 4, a = 13.1514 (8) Å, b = 16.2052 (6) Å, c = 16.8095 (10) Å, β = 105.826 (6)°, V = 3446.7 (3) Å^3, D_x =1.493 Mg m^{-3}, Cu $K\alpha$ radiation, λ = 1.54184 Å, 37436 reflections measured (Θ_{range} = 3.9-72.0°), merged to 6674 unique data, R = 0.103 [for 4675 data points with $I > 2.0\sigma(I)$], R_w = 0.219 [all data], S = 1.04. CCDC 2371743. Anisotropic displacement ellipsoids display 30% probability levels.

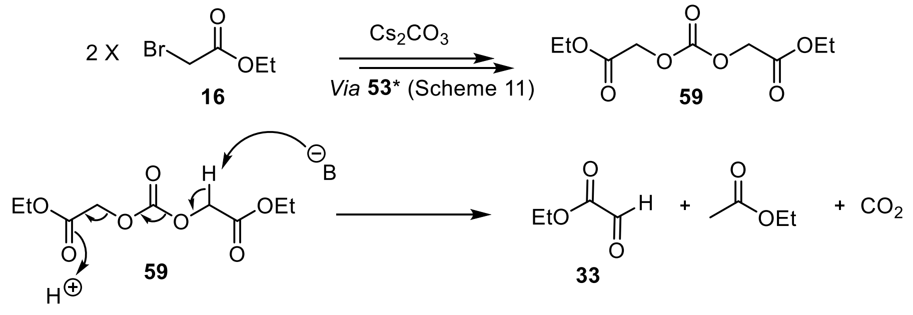

Scheme S1. Proposed synthesis of intermediates **59**, and **33**, involved in the cascade reaction indirubin **1a** and indigo **1**.
